# Improving microbial phylogeny with citizen science within a mass-market video game

**DOI:** 10.1038/s41587-024-02175-6

**Published:** 2024-04-15

**Authors:** Roman Sarrazin-Gendron, Parham Ghasemloo Gheidari, Alexander Butyaev, Timothy Keding, Eddie Cai, Jiayue Zheng, Renata Mutalova, Julien Mounthanyvong, Yuxue Zhu, Elena Nazarova, Chrisostomos Drogaris, Kornél Erhart, Borderlands Science Development Team, David Bélanger, Michael Bouffard, Joshua Davidson, Mathieu Falaise, Vincent Fiset, Steven Hebert, Dan Hewitt, Jonathan Huot, Seung Kim, Jonathan Moreau-Genest, David Najjab, Steve Prince, Ludger Saintélien, Borderlands Science players, Amélie Brouillette, Gabriel Richard, Randy Pitchford, Sébastien Caisse, Mathieu Blanchette, Daniel McDonald, Rob Knight, Attila Szantner, Jérôme Waldispühl

**Affiliations:** 1 School of Computer Science, McGill University, Montréal, QC Canada; 2 Massively Multiplayer Online Science, Gryon, Switzerland; 3 Gearbox Studio Québec, Québec, QC Canada; 4 Gearbox Entertainment Company, Frisco, TX USA; 5 Department of Pediatrics, University of California, San Diego, La Jolla, CA USA; 6 Department of Computer Science, University of California, San Diego, La Jolla, CA USA; 7 Department of Bioengineering, University of California, San Diego, La Jolla, CA USA; 8 Center for Microbiome Innovation, University of California, San Diego, La Jolla, CA USA

**Keywords:** Computational biology and bioinformatics, Microbiology

## Abstract

Citizen science video games are designed primarily for users already inclined to contribute to science, which severely limits their accessibility for an estimated community of 3 billion gamers worldwide. We created *Borderlands Science* (*BLS*), a citizen science activity that is seamlessly integrated within a popular commercial video game played by tens of millions of gamers. This integration is facilitated by a novel game-first design of citizen science games, in which the game design aspect has the highest priority, and a suitable task is then mapped to the game design. *BLS* crowdsources a multiple alignment task of 1 million 16S ribosomal RNA sequences obtained from human microbiome studies. Since its initial release on 7 April 2020, over 4 million players have solved more than 135 million science puzzles, a task unsolvable by a single individual. Leveraging these results, we show that our multiple sequence alignment simultaneously improves microbial phylogeny estimations and UniFrac effect sizes compared to state-of-the-art computational methods. This achievement demonstrates that hyper-gamified scientific tasks attract massive crowds of contributors and offers invaluable resources to the scientific community.

## Abstract (web summary)

Gamification of the multiple sequence alignment problem improves microbial phylogeny estimates.

## Main

In 2022, more than 3 billion people played video games worldwide^1^. The growth of this community is expected to continue in upcoming years. The size and diversity of the gamer community show that video games are a universal entertainment activity that transcends generations, gender and cultures^2^.

The ubiquity of video games created new opportunities to communicate and teach scientific concepts^3–7^, and one of the most remarkable applications of this media is the development of citizen science games (CSGs) that engage participants in the analysis of real scientific data. Introduced with *Foldit* in 2008, which recruited online citizen scientists to help with the prediction of the structure of proteins^8^, CSGs have been subsequently applied to comparative genomics^9^, RNA folding^10^, neuron segmentation^11^ and quantum physics^12,13^. In the last decade, CSGs had a dramatic impact on the practice of citizen science, lowering the barrier to entry of scientific activities and bringing hundreds of thousands of new participants into the community. However, successful implementation of this concept also comes with its own challenges, such as reaching potential participants and maintaining player engagement^14,15^.

The classical approach to design CSGs relies on gamification—that is, the introduction of game design elements that facilitate the acquisition of the expertise needed to complete the activity and increase engagement^16–18^. The design of these CSGs is strongly rooted in the canonical presentation of the scientific task, which may hinder its entertainment value and, therefore, participation of the general public. Hence, their target audience focuses on users with prior interest in science^15^, which eventually amplifies the demographic skew observed in citizen science projects^19^.

To answer to the challenge of user acquisition and engagement in citizen science, in 2015, Massively Multiplayer Online Science (MMOS) proposed to embed citizen science activities into commercial video games played by massive established gamer communities^20^, relying on the leadership of game designers to ensure a seamless integration of the citizen science activity in the host video game user experience. The first incarnation of this concept was *Project Discovery* in the massively multiplayer online game *EVE Online* in 2016 (ref. ^21^). *EVE Online* has a user base with a welcoming stance toward scientific narratives, and it had not yet been shown that citizen science can work in any type of video game, specifically gamer communities not familiar with scientific narratives.

In the present work, we reinforce the primacy of game design to overcome the challenges of integration of citizen science in commercial games. We also leverage our previous experience with *Phylo*^9,22,23^ to develop a CSG for microbiome data analysis that follows ‘game-first design’ principles. This approach aims to create a pure game that integrates scientific computation mechanisms in its core gameplay rather than adapting existing scientific research modus operandi (Supplementary Information, section 6). Eventually, this methodology can lead to fundamental alterations in the canonical presentation of the data that will prevent the complexity of the scientific task from obfuscating the accessibility or altering the user experience. We found that this approach of redesigning the original task with a fresh ‘untrained’ eye can bring valuable improvements from these other disciplines of game or narrative design.

This approach results in massive gains in public participation without sacrificing the relevance of the collected answers. More importantly, it provides access to new human computation resources capable of executing complex computational tasks on large scientific datasets, which would otherwise be unfeasible with classical CSGs.

**Fig. 1:**
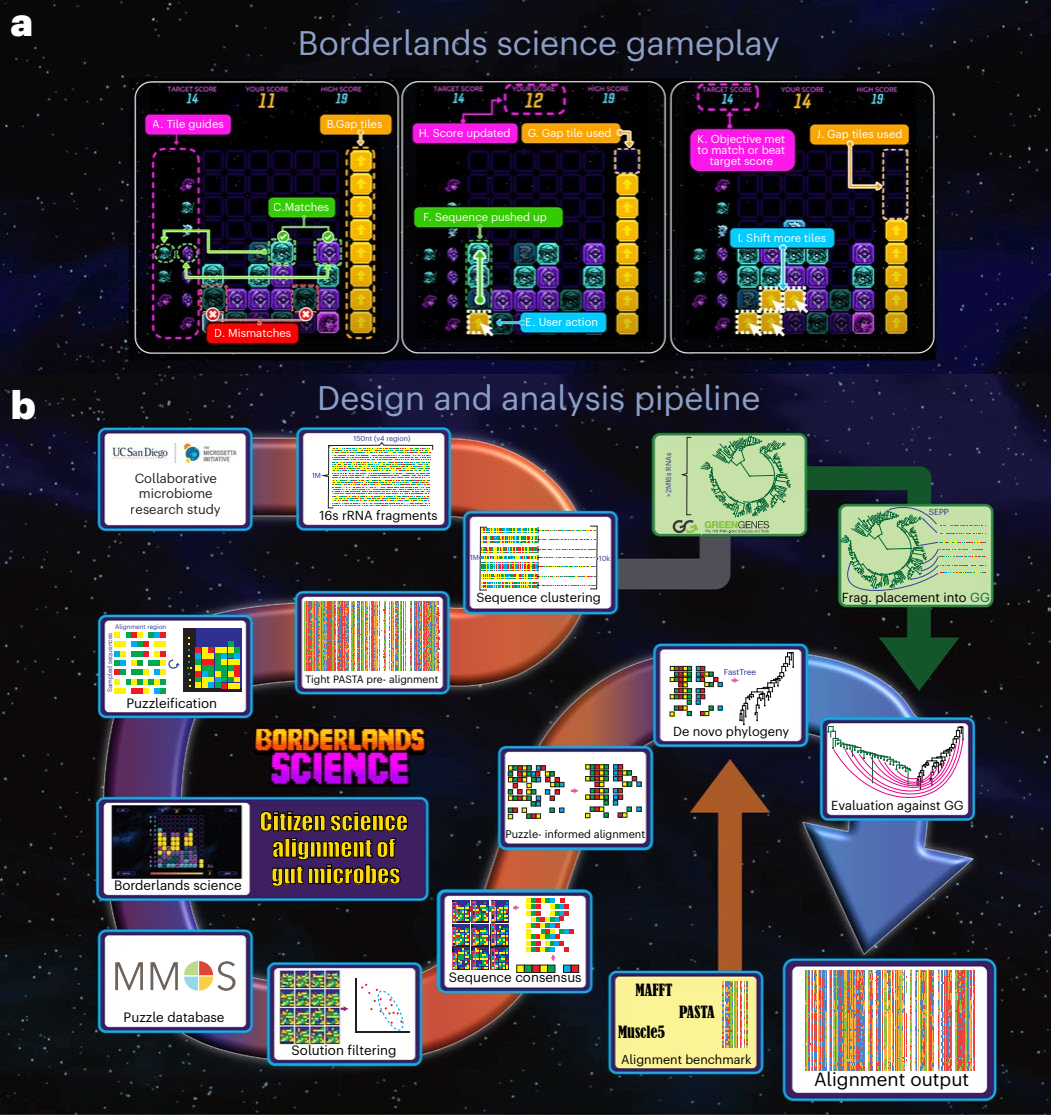
The *BLS* game. In **a**, we present the *BLS* gameplay. Players are tasked with aligning the colored bricks, representing nucleobases, to the guides on the left, by inserting yellow gap bricks. They receive a bonus for full rows and must reach the par score to progress to the next puzzle. In **b**, we show the *BLS* pipeline from data collection to alignment output, in particular how data flow from the initial alignment of 1 million sequences to the analysis results featured in this paper. The Supplementary Information provides details pertaining to each step, regarding game design, puzzle generation, filtering and realignment.

We showcase this concept with *Borderlands Science* (*BLS*; Fig. 1)^24^, a tile-matching mini game released on 7 April 2020 as a free downloadable content for *Borderlands 3* (the most recent release of a video game franchise that sold over 77 million copies worldwide). *BLS* is designed to improve a reference multiple alignment of 1 million 16S ribosomal RNA (rRNA) sequences from The Microsetta Initiative’s (TMI) American Gut Project (AGP)^25^, a citizen science initiative collecting gut microbial genomic data.

Since its launch, more than 4 million gamers have played *BLS* and solved more than 135 million puzzles. Our results show that the multiple sequence alignment (MSA) obtained from *BLS* (Fig. 1b and Methods) improves microbial phylogeny inference compared to other alignment methods and enables improved UniFrac^26^ effect size estimation for many variables known to be important to human digestive health. Beyond these results, *BLS* demonstrates that commercial video games can provide massive human processing resources for the manual curation and analysis of large-scale genomic datasets that could not be completed otherwise.

## Results

### Task and data

The data featured in this article come from a set of 953,000 rRNA fragments sequenced from stool samples submitted by participants in the AGP^25^, powered by TMI (https://microsetta.ucsd.edu/). The fragments are 150 nucleotides long and come from the V4 region of the 16S rRNA gene.

In the *BLS* mini game (Fig. 1a), the player sees 7–20 sequences of 4–10 nucleotides. Each sequence is displayed as a vertical pile of bricks, each color representing a nucleotide (through a random mapping between colors and nucleotides). The bricks are collapsed (all gaps are removed), and the player is asked to insert a finite number of gaps to improve a score determined by the number of bricks correctly aligned to the guides. These targets, located on the left, display the most common nucleotides in the corresponding alignment column. By inserting gap tokens, the player is using their natural knack for pattern matching to realign a region of the scaffold alignment. The limited number of tokens, set by a naive greedy artificial intelligence (AI) player based on how easily it could improve the alignment, forces the player to make tough choices. This greedy player also sets a target score that the player must beat to progress to the next puzzle, enforcing a minimal effort.

During the first year of the initiative, we collected approximately 75 million puzzle solutions (mean, 43 per puzzle). The solutions were used as ‘votes’ by players on potential errors in the scaffold alignment, and a corrected alignment was generated. Here we report results for our *BLS* alignment and benchmark alignments produced by several state-of-the-art de novo multiple alignment programs (PASTA, MUSCLE and MAFFT) as well as an alignment produced by a greedy algorithm (Methods and Supplementary Information, sections 12–15).

### Complex citizen science tasks can be embedded in video games

Integrating a scientific task in a commercial video game from a franchise that sells millions of copies constitutes a risk. The scientific mini game must not look out of place in the broader game as it could break immersion, an important component of role-playing games. This is especially true when integrating a puzzle game into a shooter–looter role-playing game such as *Borderlands 3*, in which the player expects to face fast-paced action and humor. *BLS* was specifically designed to address this potential issue by integrating dialogues with characters from the *Borderlands* universe. The virtual arcade booth was located in a virtual laboratory within the game universe and is presented as belonging to the resident scientist. The gameplay was simplified to make sure that the pace matched a shooter–looter game, and all the visuals were specifically designed to make sense in-universe.

As of May 2023, 3 years after launch, 4.45 million players had visited the arcade booth. Of these players, over 4 million completed the 10-min tutorial and at least one real task. This represents an engagement rate of 90%, which is a substantial improvement over *Phylo*, the previous sequence alignment CSG, which has reported an engagement rate of around 10%. This level of player engagement demonstrates that the ultra-gamification of the task worked and that its integration into the commercial game was seamless.

### *BLS* outputs a high-quality MSA

The evaluation of the *BLS* alignment output is complicated by the absence of a universal ground truth to benchmark against. Nevertheless, there are several qualities typically expected from a high-quality alignment that we can investigate. Indeed, a high-quality alignment of homologous genomic sequences tends to:

- Have a high sum-of-pairs score
- Have a gap frequency compatible with that RNA family’s indel frequency
- Allow the inference of phylogenetic trees that resemble the state of the art for that family
- Appropriately separate taxa associated with different illnesses, behaviors and profiles in the host
- Be compatible with the structural signature of the family

We assert that the *BLS* alignment satisfies all these criteria and, thus, constitutes a high-quality MSA.

In Table 1 (top), we show that *BLS* improves the sum-of-pairs score compared to all benchmarks when excluding heavily gapped columns from the scoring, thus satisfying **a**.

**Table 1:** Phylogeny and alignment information

Method	PASTA	Post-processsing PASTA	MUSCLE	MAFFT	Greedy algorithm	*BLS*
**Phylogeny and alignment information**						
Kendall–Colijn mean	2,193	1,521	1,772	1,298	1,246	1,115
Triplet distance mean (*k*)	86.6	73.4	101.4	80.9	80.2	52.2
Compound Kendall–Colijn and Triplet	87.2	67.1	86.1	66.4	65.0	48.4
Sum of pairs (billions)	2.09	2.18	2.05	2.11	2.16	2.17
Alignment width	193	188	260	270	198	196
**Proportion of bases lost in mapping to structure**						
Proportion of unmapped bases	0.001		0.149	0.100		0.003

**Fig. 2:**
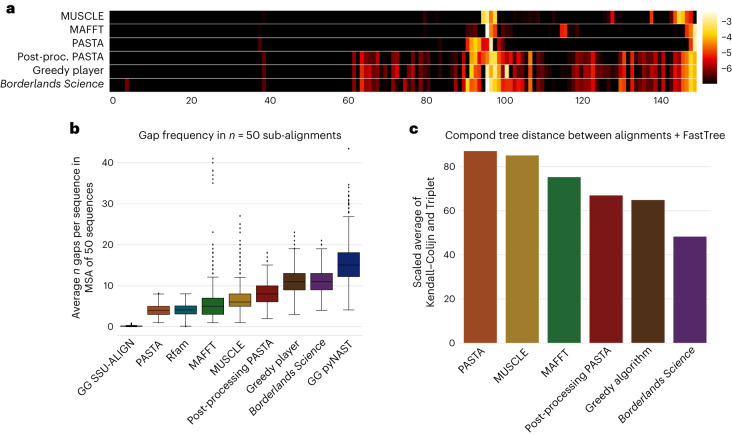
Evaluation of the alignment. **a**, Here the gap density by column is shown for six different alignment methods. The *x* axis corresponds to the alignment position, and the color corresponds to the log gap frequency at this position. Highly gapped columns were excluded so all alignments could be of the same length. **b**, Gap frequencies observed in *BLS* and other methods, averaged from sampled sub-alignments of 50 sequences. The box plot shows an overview of the distribution. The three horizontal lines in the box, from top to bottom, show, respectively, the boundaries for the upper quartile, the middle quartile and the lower quartile. The total height of the box represents the interquartile range (IQR). The whiskers are located 1.5 times the IQR from the ends of the box. The dots outside the whiskers are the outliers. Note: one outlier point for the right-most distribution, pyNAST, with a value of 124, is not shown on the plot but is considered in the statistics shown. **c**, Compound distance to the reference Greengenes tree. That compound metric was obtained as a scaled average of the Kendall–Colijn and Triplet distance. More detail is provided in Supplementary Information, sections 12a,b and 13c. GG, Greengenes.

Top: The reported distances are the mean of sampled distances from phylogenetic trees estimated with FastTree from alignments generated with various methods against our reference tree, the Greengenes-SEPP tree. The compound distance is a combination of Kendall–Colijn and Triplet. Because these are distances, optimal values are the lowest. For the sum of pairs, the optimal value is the highest. We also report alignment width for context. Bottom: Alignments were reduced to the 150 most populated columns (same as in Fig. 2a) to be mapped to the 16S V4 secondary structure. We report the proportion of bases in unmapped columns.

In Fig. 2b, we show the gap frequency sampled from sub-alignments (we sample sub-alignments to account for differences in number of sequences among Greengenes, Rfam and *BLS*) for *BLS* and benchmarks. These benchmarks include PASTA^27^, MUSCLE^28^ and MAFFT^29^, the pyNAST^30^ and SSU-ALIGN^31^ alignments from the Greengenes^32^ database and the structural Rfam^33^ alignment. We observed that all alignments had a gap frequency in the same order of magnitude as PASTA, Rfam and pyNAST. This low gap frequency is consistent with the strongly structured nature of the V4 region, which varies in length by only a few base pairs between microbes. Thus, the *BLS* alignment shows a gap frequency that is compatible with our state-of-the-art knowledge of the V4 region, and criterion **b** is satisfied.

Our results in Table 1 and Fig. 2 indicate that the additional gaps inserted by *BLS* compared to PASTA, MAFFT, MUSCLE and the greedy algorithm led to a improvement of the tree structure.

We present our investigation of criteria **c**, **d** and **e** in the following subsections.

### The *BLS* alignment improves de novo phylogeny

A central objective of improving alignments of microbial genomic sequences was to better understand their phylogeny. To assess whether this goal was achieved, we inferred phylogenetic trees from our alignments with FastTree^34^ and then assessed their similarity to a reference tree built by placing our sequences into the Greengenes 13.5 (ref. ^32^) phylogeny with SEPP^35^. Greengenes has been previously benchmarked for fragment insertion, and SEPP with Greengenes has been shown to outperform de novo phylogeny inference, when a high-quality tree and alignment are already available^36^.

Our phylogenetic similarity results, shown in Table 1 for two distance metrics, Kendall–Colijn^37^ and Triplet^38^ distance, indicate that the *BLS* phylogenies are closer to the reference than standard MSA approaches. This result shows that the *BLS* alignment outperforms alternatives for the task of inferring a reliable phylogeny, thus satisfying criterion **c**.

### The *BLS* phylogeny leads to improved UniFrac effect sizes

We also formulated the hypothesis that improved alignments (and, by extension, de novo phylogenies) would lead to some improvement in the separation of taxa associated with different behaviors, profiles and diseases. To confirm this, we measured effect sizes on UniFrac^26^ distances over 74 non-technical variables available in the AGP metadata associated with the samples used for sequencing. These variables relate to the host’s lifestyle, health condition, food or general profile.

**Fig. 3:**
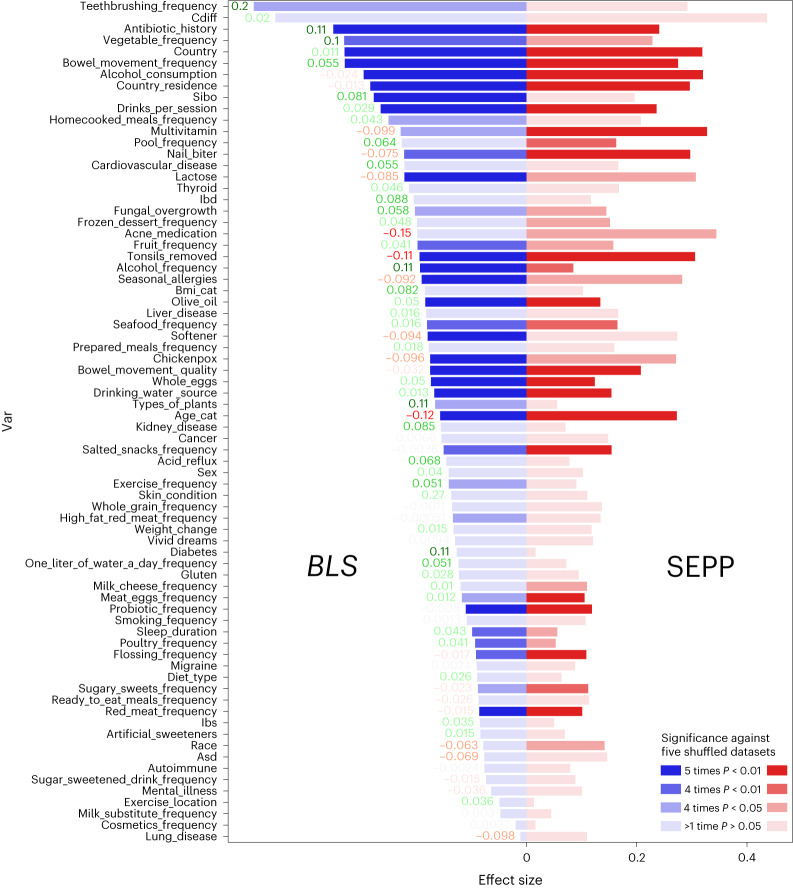
Effect sizes. Delta means pairwise effect sizes between *BLS* and SEPP for each variable. Significance, indicated with the transparency, refers to the *P* value obtained from a two-tailed Mann–Whitney *U*-test against shuffled metadata. More detail is provided in Supplementary Information, section 14.

The strongest effect sizes that we observed were for teeth brushing frequency and prior *Clostridium difficile* infection (full list in Fig. 3 and Supplementary Information, section 14). We report the average pairwise effect sizes between *BLS* and Greengenes + SEPP in Fig. 3. We observed that *BLS* outperforms SEPP on many variables, including several that have been linked to gut microbe diversity and human health^25,39–42^. The top five variables with highest delta are, respectively, teeth brushing frequency, diabetes, number of types of plants, antibiotic history and alcohol frequency.

We backed up this analysis by assessing whether the effect sizes obtained were significantly different than what could be observed in a random assignment of categories to samples. We annotated Fig. 3 with the *P* values associated with the null hypothesis that our results are compatible with a random outcome. We observed an enrichment of significant outcomes in the variables with high effect sizes and, in particular, 13 variables for which *BLS* achieves a higher significance category than SEPP, whereas the opposite occurs 10 times, generally on variables with lower effect sizes.

These improvements observed on most variables confirm that the previously reported improvements to phylogeny can be perceived in meta-analyses. Although overall improvement over SEPP is limited as SEPP still outperforms *BLS* on important variables, such as age category, *BLS* does outperform SEPP, and the two approaches lead to distinct solutions that are complementary in improving understanding of gut microbe phylogeny and its impact on human health, thus satisfying criterion **d**.

### The *BLS* alignment improves support for 16S rRNA structure

As stated previously, the structure is an important component of a high-quality MSA of a strongly structured RNA region. Given that the *BLS* alignment contains about 99% of bacterial sequences, it is possible to map its columns to the bases of the state-of-the-art structural model of the 16S bacterial rRNA defined on the Comparative RNA Web^43^.

To estimate the quality of such mapping, we report the proportion of non-gap nucleotides that cannot be mapped to the structure (see Table 1, bottom). It turns out that alignments such as *BLS* and PASTA map rather easily, whereas MUSCLE and MAFFT lose substantially more information.

**Fig. 4:**
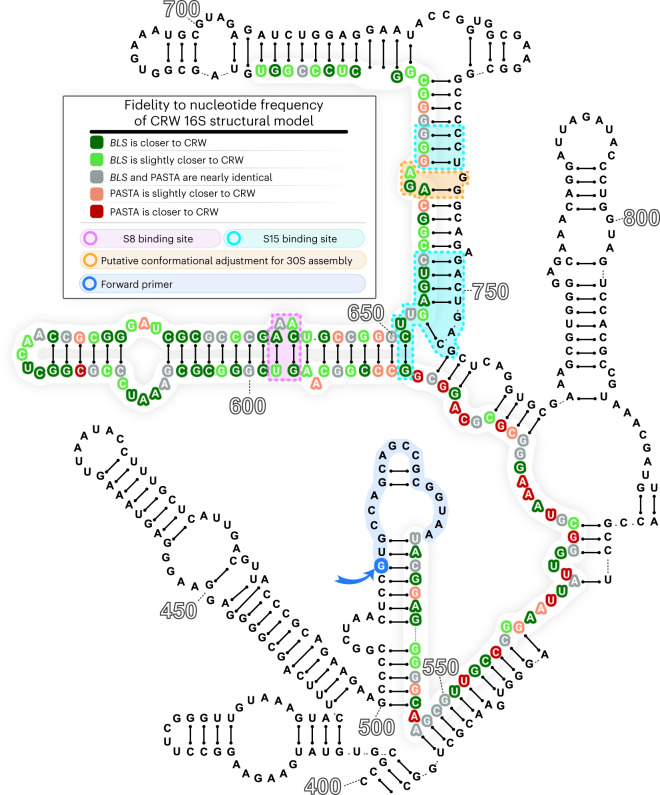
Agreement with 16S rRNA structural model. After mapping the *BLS* and PASTA alignments to the 16S structural model, we observed a higher conservation for *BLS*, especially near important functional sites. The figure shows an annotated 16S secondary structure where only the V4 region and its vicinity are shown. Colored bases form a continuous backbone. CRW, Comparative RNA Web.

To deepen our evaluation of the structural quality of the two alignments that map well, we investigated column by column whether the differences between *BLS* and PASTA alignments tend to agree or disagree with the model. In Fig. 4, we show that the changes added by human players tend to agree with the 16S structural model, especially in regions linked to important functional sites, such as S8 and S15 binding sites, and a region that undergoes conformational readjustment during 30S assembly^44–49^. This agreement is a strong argument in favor of the *BLS* alignment as the CRW model considers long-range base pairs, a context that is not available to the players solving small puzzles. This is important because, as shown in Fig. 2a, the region of the alignment with the most gaps and the most variation between methods is the top of the V4 stem, a region with considerable mapping improvements for *BLS* in this analysis.

This demonstrates that the modifications to the PASTA scaffold added by *BLS* led to an improvement of the structural and functional signal of the alignment, thus satisfying criterion **e**.

Additionally, in Fig. 2a, we show the gap frequency per column (excluding highly gapped columns) of different alignment methods, including *BLS* and our benchmarks PASTA^27^, MUSCLE^28^ and MAFFT^29^. These gap frequencies help reveal the differences between *BLS* alignments and alternatives, on top of satisfying criteria **a**–**e**.

## Discussion

**Fig. 5:**
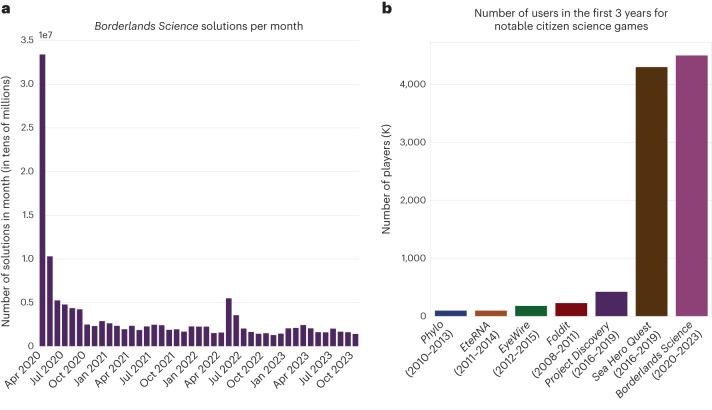
Player engagement. This figure provides context on player engagement. **a**, We show the number of solutions received from player per month after the release of the game, showing continuous engagement after the end of the initial rush. **b**, We compare the number of participants in *BLS* to some notable recent CSGs, over the first 3 years of existence, showing the impact of the integration into a mass-market game (*Sea Hero Quest* and *BLS*) compared to more traditional CSGs. See Supplementary Information, section 11, for sources for the numbers presented in **b**.

Since the release of *Foldit* in 2008, many high-impact CSGs have been released, such as *EteRNA*^10^, *Project Discovery*, *EyeWire*, *Phylo* and *Sea Hero Quest* (Fig. 5).

To this day, a CSG that would reach 500,000 players would be considered a massive success. This evaluation is made in context of the relative difficulty for a scientific team to reach people who are likely to be interested in playing a game for science.

*Galaxy Zoo*^50^ was the first initiative of this type to break 1 million participants after 10 years in activity. *Sea Hero Quest* was the first to achieve the feat in less than 1 year, by working with Deutsche Telecom, a company with 300 million customers^51^. *BLS* built on this idea of improving reach through collaboration with non-scientific companies and went to the next level by reaching gamers directly where they are, within the virtual universe of high-profile video games.

Now that we have shown that puzzle-style CSGs can be seamlessly integrated into a high-profile game and engage players, we expect other projects to take advantage of this opportunity to enter an era where million-players CSGs are no longer an exception.

*Phylo* struggled to retain players for more than a couple puzzles, with an average of 5.7 tasks completed by users, and a median of two. Thus, *Phylo* relies heavily on expert players. Indeed, in *Phylo*, the experts can collectively outperform casual gamers by up to 40% depending on the alignment, despite being a small group completing a tiny fraction of the tasks solved by casual players. On the other hand, *BLS* players complete an average of 35 tasks, with a median of 12. Unlike in *Phylo*, where experts achieving high scores form the backbone of the scientific contribution, *BLS* filters tasks based on consensus between players and provides rewards for just reaching a target score. Therefore, *BLS* does not rely on experts to the same degree. Indeed, removing all contributions of the 10% most active players represents only a 14% drop in evaluation metrics compared to removing the same amount of data at random.

This lack of reliance on experts marks a change of paradigm from lightly gamified CSGs that rely on experts to a new category of CSGs to which everyone can contribute.

Two core objectives guide the design of CSGs: maximizing player engagement and maximizing the scientific relevance of the game. Unfortunately, these two objectives often conflict with one another; a game with optimal scientific relevance would be a pure crowdsourced scientific task with no gaming elements, such as image annotation in Stardust@home. Gamification of the task, by design, will make mapping player solutions to scientific contributions more difficult. However, the more gamified the task, the higher the potential for it to play like a real game and be fun enough to stimulate player engagement and retention. In the present study, we pushed the envelope on gamification, and our observations match the conundrum: *BLS* achieved a very high level of player engagement, but the average scientific contribution of one task was lower than in *Phylo*, where a few hundreds of puzzles are sufficient to improve a small alignment. Here, millions of tasks are combined to improve alignments. Nevertheless, the ultra-gamification of *BLS* led to a much higher player engagement and retention than in *Phylo*, which strongly alleviates that downside.

Another aspect that we think indicates potential for growth in CSGs is the feedback that we received from the players. Through our exchanges with gamers in the form of quality assurance testing feedback, blog posts, email and social media (Supplementary Information, section 11.5), the most common response that we received was enthusiasm and curiosity about science. Although quantifying this attitude would be its own study and, thus, left for future work, our experience in this project leads us to think that player engagement was driven by a combination of game-first design and genuine interest in science from the public.

Moreover, beyond the optimization of the tradeoff described above, the inherent benefits of participating in a citizen science initiative, such as improvements in science literacy and increased connectedness of the public to the scientific world, further justifies the exploration of the extra gamification region of that tradeoff: reaching more participants is a valid objective in its own right (Supplementary Information, section 11.4).

In the present study, we focused on phase 1 of *BLS*, which includes data from the first year, and over two-thirds of the total amount of data. There are, thus, 2 years of data remaining to analyze. These data include different types of puzzles aimed at studying how sequence alignment CSGs can be made more efficient. After phase 1, we started producing puzzles with alternative scaffolds, and, as such, the analysis presented here does not apply to them, which is why they are not included in this study (Supplementary Information, section 5).

Additionally, we included a guide offset in some of the puzzles sent to users (Supplementary Information, section 5.2.6). Along with presenting more stimulating gameplay, these guide offsets allow the user to correct potential errors in the scaffold alignments at a larger scale. As a result, they are more noisy than regular puzzles (Supplementary Information, section 8.5.4). *BLS* alignments built with offset puzzles show potential, as they outperform *BLS* on some important effect size variables, such as age category. Nevertheless, because they require a different type of analysis, their investigation is left for future work.

Another consideration for future work is the integration of synthetic data in the game. Indeed, in the present study, we relied on the Greengenes phylogeny as a proxy for a ground truth. Although this phylogeny is well understood and state of the art in the field, it is not perfect. In future projects of this type, we will endeavor to integrate synthetic data into the design of the game, to complement our proxy for the ground truth. Nevertheless, it must be mentioned that synthetic data often embed assumptions that match the true evolutionary process poorly, especially under heterogeneous substitution models in different parts of the tree and when RNA secondary structure is present, both of which we know are the case in this situation.

Finally, we have been working in parallel on strategies to extend the impact of player participation by training reinforcement learning agents to reproduce player strategies, and, recently, we published two studies on the matter. In the first study^52^, we established that it is possible for reinforcement learning agents to learn player strategies for solving *BLS* puzzles, unlocking potential for propagating these player strategies to data outside of the game. In the second study^53^, we demonstrated that injecting human solutions into a Q-learning algorithm outperforms pure AI. Combined with the aggregation techniques presented in the present study, these results suggest promising avenues to design an automated pipeline for massive alignment of 16S rRNA sequences.

## Conclusion

In the present study, we present *BLS*, a sequence alignment game aiming to open a path for gaming-focused CSGs targeting large-scale problems with large-scale audiences. The project achieved player engagement unseen before for a game of its category and reached its goal of pooling gamers together to solve a large-scale collective task while also serving as a large-scale science outreach initiative. We showed that, despite being gamified to a degree beyond that of previous efforts, the contributions of the players led to clear improvements over state-of-the-art sequence alignment methods, unlocking the potential for a new generation of gaming-focused CSGs.

## Methods

The data featured in this article come from a set of 953,000 rRNA fragments sequenced from stool samples submitted by participants in the AGP^25^, powered by TMI (https://microsetta.ucsd.edu/). The fragments are 150 nucleotides long and come from the V4 region of the 16S rRNA gene.

The 953,000 150-nucleotide V4 fragments were first clustered with CD-HIT^54^ into 10,200 clusters. Very dissimilar clusters representing a small minority of sequences (about 5,000) were removed, and a final set of 9,667 cluster representative sequences was obtained, which accounts for 946,740 sequences of the initial dataset.

These 9,667 sequences were then aligned with PASTA 1.9 (ref. ^27^), generating a very tight (183 columns) initial alignment, which served as a scaffold for the creation of CSG puzzles.

### Game design

In the *BLS* mini game, the player sees 7–20 sequences of 4–10 nucleotides (the size of the grid changes with the difficulty level). Each sequence is displayed as a vertical pile of bricks, each color representing a random base. The bricks are collapsed (all gaps are removed), and the player is then asked to insert a finite number of gaps to improve a score determined by the number of bricks correctly aligned to the guides. These targets, located on the left, display the most common nucleotides in the corresponding alignment column. By inserting gap tokens, the player is using their natural knack for pattern matching to realign a region of the scaffold alignment. The limited number of tokens, set by a naive greedy AI player based on how easily it could improve the alignment, forces the player to make tough choices. This greedy player also sets a par score that the player must beat to progress to the next puzzle, enforcing a minimal effort. The game is displayed in Fig. 1, and more details on game design can be found in the Supplementary Information (section 6).

### Building puzzles

Puzzles were added to the game on a regular basis. The results presented in this paper use the solutions of the first 1.4 million puzzles (a total of about 75 million player tasks), submitted to the players between April 2020 and July 2021. To get a wide enough distribution of solutions, each puzzle was released in three different versions, each with a slightly different number of gap tokens available. Each different version was aimed to be played by about 15 players, for a total of 45 per puzzle.

For each alignment region (a sequence of alignment columns of variable length), sequences are sampled from the PASTA alignment. To compensate for the bias that comes from the vertical orientation, puzzles are built both from left to right and from right to left. Additionally, each sequence in each puzzle has a small probability (~0.15) to be shortened or elongated to show puzzle sequences of different lengths, allowing the player to simulate deletions.

Finally, an offset was sometimes introduced between the guides and the puzzle sequences. An offset of 1 would mean that puzzles using sequences from the alignment columns 10–14 would have the guides from columns 9–13, to enhance the user’s action space; they would then have to push up many bricks just to get back to the initial PASTA configuration. Through these variations in the puzzles, the players are given opportunities to re-evaluate alignment decisions made by the PASTA algorithm.

### Puzzle and solution filtering

Puzzles are discarded if they offer little incentive to move bricks because the starting gravity effect already yields a local optimum. This led to some alignment regions being overrepresented in the puzzles due to a higher acceptance rate, which we interpreted as these regions presenting more room for improvement via puzzles.

For each puzzle, the 20–60 (objective, 45; real mean, 43.4) user solutions were filtered by their similarity to the distribution of gap positions from all submitted solutions to that puzzle. The player solutions were filtered using two criteria: (1) how far from optimality the solution was and (2) how far from the player consensus the solution was. The distance from optimality in (1) was defined as the score difference against the solution with the highest score using the same number of gaps for that puzzle. The player consensus in (2) was defined as the centroid of the distribution of solutions. Solutions that were too far from this combined objective were excluded, and the exclusion threshold was set to exclude about two-thirds of the solutions, to keep a subset that was most representative of the ensemble of high-quality player solutions. This threshold was selected experimentally based on visual exploration of rejected solutions: we selected a threshold for which no obvious outliers were included, and all excluded solutions were clear outliers.

### Alignment improvement

The puzzle solutions are converted to positional sequence annotations, which are normalized to account for variance in coverage, and unannotated positions are filled with PASTA and/or Rfam version 14 (ref. ^33^) alignment information.

We consider each high-quality annotation from one puzzle solved by one player as a vote. Our algorithm leverages dynamic programming through a compromise between Needleman–Wunsch^55^ and the voting system, in which we align a sequence to the array of votes.

### Validation

Because the alignments produced by *BLS* are very different from the ones produced by state-of-the-art software, such as MUSCLE version 5.1 (ref. ^28^) or MAFFT version 7.490 (ref. ^29^), it is difficult to fairly compare them as alignments. Thus, the validation process for our methods is centered around the comparison of phylogenetic trees estimated from the alignment with FastTree 2.1 (ref. ^34^).

#### Small alignment benchmark

Along with the PASTA alignment that we computed as the scaffold for the puzzles, we ran MAFFT and MUSCLE on the same 9,667 sequences using both the standard and slowest settings. We then generated phylogenies for all with FastTree’s standard settings. These trees were then re-rooted using sequences classified by naive Bayes with QIIME version 2’s q2-feature-classifier^56^ against Greengenes 13.8 (ref. ^32^) with Archaea as the outgroup.

#### Comparison to SEPP

The state-of-the-art method for building phylogenetic trees from alignments has been shown to be outperformed on gut microbiome data by the placement of the sequences into an existing tree with SEPP^35,36^, which, when sufficient data are available, outperforms de novo phylogeny estimation from alignments^36^. To build a reference phylogeny, we placed 9,667 sequence cluster representatives into the Greengenes 13.5 phylogenetic tree with SEPP and then removed all the other tips and re-rooted the tree to Archaea. We refer to this tree as the Greengenes-SEPP reference tree.

We compare the de novo alignment methods to this Greengenes-SEPP reference tree by computing distances between randomly sampled subtrees of the de novo and reference trees, using two distance metrics:

Kendall–Colijn metric^37^, which compares the placement of the most recent common ancestor of each pair of tips, specifically designed for assessing similarity in evolutionary patterns and specifically designed for situations where the Robinson–Foulds distance is not sensitive enough.

Triplet distance^38^, a metric that measures the structural dissimilarity of two phylogenetic trees by counting the number of rooted trees with exactly three leaves that occur in one but not both trees.

When comparing large trees, because these distances are very computationally expensive, we sample 400 nodes for Kendall–Colijn and 100 nodes for Triplet. This process is repeated, respectively, 100 and five times. We performed these evaluations with the R treeDist^57^ library, version 2.7.0. Trees were resized and sheared in Python 3.8 with scikit-bio version 0.5.

#### Application to meta-analyses

To assess the impact of the *BLS* alignment on meta-analyses involving gut microbiome, the alignment results were propagated from cluster representatives to other sequences to build an alignment of 946,740 sequences, for which a phylogenetic tree was estimated with FastTree. To assess effect sizes, a feature table representing the AGP data was obtained from redbiom version 0.3.5 (ref. ^58^), filtered for blooms and rarefied to 1,000 sequences per sample, and unweighted UniFrac^26^ distances were computed for each tree (for example, the *BLS* trees, de novo trees and the SEPP fragment insertion tree). Pairwise effect sizes were then calculated with Evident version 0.4.0 (ref. ^59^) on the distance matrix using Cohen’s *d* distributions for each tree. The pairwise effect sizes of 74 non-technical variables were investigated (all variables available on QIIME 2 meeting a minimum of two categories including 50 samples). We reproduced these experiments with shuffled metadata to assess significance. We also examined two technical variables—the sample plates and the liquid handling robot—and observed no significant change.

It should be noted that the aggregation of player solutions and the production of an alignment were not optimized for performance on effect sizes. This choice aims to avoid overfitting by training to optimize for a phenotype, due to the relatively small size of those data. Furthermore, it was established in previous work that there is a relationship between the quality of the tree and the observed phenotype (effect sizes)^36^. Thus, alignments were optimized on tree metrics, and effect sizes were kept as an independent evaluation. As expected from previous work, the *BLS* alignment with the best tree metrics (listed in Table 1) also had the best effect sizes.

### Ethics statement

Participation in the project was done entirely virtually, through the *Borderlands 3* game. It was fully optional. No participants were recruited, and potential participants were informed of how and why the data would be used through a presentation video played before gameplay. Only gameplay data were collected. No identifying or personal information about the participants was collected, and participants were fully anonymous to the research team.

### Reporting summary

Further information on research design is available in the Nature Portfolio Reporting Summary linked to this article.

## Online content

Any methods, additional references, Nature Portfolio reporting summaries, source data, extended data, supplementary information, acknowledgements, peer review information; details of author contributions and competing interests; and statements of data and code availability are available at 10.1038/s41587-024-02175-6.

## Supplementary information

Supplementary Methods, Results and Discussion.

Reporting Summary

## Data Availability

All the data discussed in this paper are publicly available for scientists and individuals at https://games.cs.mcgill.ca/bls/. The data are also publicly available on mirror sites at https://gitlab.com/borderlands-science/BLS1 and 10.6084/m9.figshare.24962349 (ref. ^60^). These data include all the puzzles that players solved, all the solutions submitted by the players and related data, such as the order in which they made their moves to solve the puzzle. The players are identified by unique alphanumeric strings as it was not possible to obtain their informed consent to share their personal information, as these data were collected through a video game.

## Code availability

All the code used to generate data, process data and compute results presented in this paper is freely available as a GitLab repository linked on the project website: https://games.cs.mcgill.ca/bls/. The code is also publicly available on mirror sites at https://gitlab.com/borderlands-science/BLS1 (ref. ^61^) and 10.6084/m9.figshare.24962349 (ref. ^60^).
