# Supplementary material for: Improving microbial phylogeny with citizen science within a mass-market video game
Source: Nat Biotechnol. 2024 Apr 15;43(1):76–84. doi: 10.1038/s41587-024-02175-6 (PMC11738981; doi:10.1038/s41587-024-02175-6)
Supplement: Supplementary file 1 — Supplementary Methods, Results and Discussion. [file 41587_2024_2175_MOESM1_ESM.pdf]

nature biotechnology

Article

https://doi.org/10.1038/s41587-024-02175-6

# Improving microbial phylogeny with citizen science within a mass-market video game

In the format provided by the authors and unedited

# Borderlands Science - Supplementary material

This document contains a repository of the information about our methods that we were not able to fit in the main text. It is organized in the order of the main pipeline figure (Figure 2 in the main text); each section here corresponds to a step on the wide arrow.

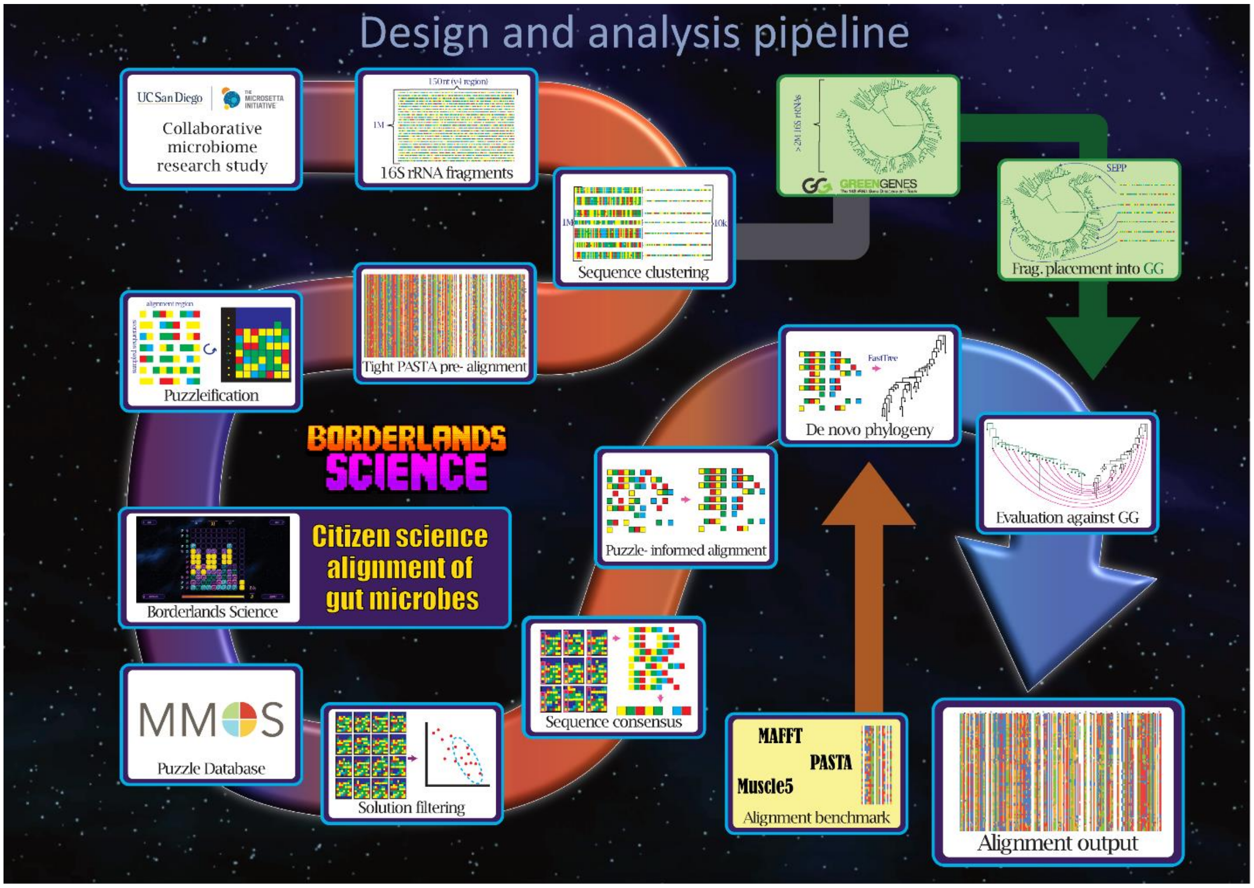


A detailed table of contents follows on the next page.

**Quick access:**

- Click here for the Borderlands Science publicly available data
- Click here for the Borderlands Science publicly available code
- Click here to learn more about Gearbox Entertainment
- Click here to learn more about the scientific team behind Borderlands Science at McGill University
- Click here to learn more about the Microsetta Initiative
- Click here to learn more about Massively Multiplayer Online Science

# Borderlands Science - Supplementary material

## PART 1: DATA

1. The American Gut Project

2. The data: 16S rRNA Fragments & metadata

## PART 2: METHODS

3. Sequence Clustering

4. Tight PASTA scaffold alignment

5. Puzzleification

6. The Borderlands Science Game

    6.1 Game Design: adapting Phylo to the Borderlands Universe

    6.2 The Borderlands Science gameplay

7. Puzzle Database

8. Solution Filtering

9. Sequence Consensus

## PART 3: VALIDATION & DISCUSSION

10. Puzzle-informed Realignment

11. A game changer for citizen science

12. How can we evaluate alignments?

13. Evaluating alignments via phylogeny

14. Evaluating alignments via effect sizes

15. Evaluating alignments via structure

16. Understanding effect size results

17. Other Borderlands Science publications

18. References

# 1. The American Gut Project

The data used in the project comes from genome fragments sequenced from stool samples submitted by participants to the American Gut Project (AGP) initiative (McDonald et al. 2018).

AGP is a citizen science initiative with the goal of advancing microbiome research. The human microbiome has a fundamental impact on human health. Aiming to support precision medicine and educate the general public about this crucial aspect of human health, this project was motivated by a lack of knowledge regarding the margins of bacterial diversity within the human population and the relative importance of lifestyle, health conditions, and food. Researchers have compared samples of the human microbiome, mostly from the US, UK, and Australia, with samples of the environment from the Earth Microbiome Project (Gilbert, Jansson, and Knight 2014) and samples submitted by more than 10,000 citizen scientists. The AGP revealed novel compounds and various types of molecular communities in the human stool metabolome and demonstrated the existence of a greater spectrum of beta diversity among human stool microbiome in comparison to environment samples. (McDonald et al. 2018)

The Microsetta Initiative (TMI), based at the Knight lab at the UC San Diego School of Medicine, was born as a result of the AGP. Along with the Center for Microbiome Innovation at UC San Diego, it is a highly collaborative, multinational effort. To further our understanding of the human microbiome, TMI's objective is to include citizen scientists in microbiome research, educate through outreach, and provide a significant resource for future scientific efforts.

# 2. The data: 16S rRNA Fragments & metadata

The genome fragment dataset presented in this project consists of the first 150 nucleotides of the V4 region in the 16S rRNA gene. This region in particular was selected because it has been previously established to be a good proxy for the evolutive behavior of the full 16S rRNA sequence for human microbiota (Van Der Pol et al. 2019). The raw 16S rRNA amplicons were denoised using Deblur in Qiita and expressed as amplicon sequence variants (ASVs)

The dataset is constituted of 951,445 such sequences, obtained from stool samples contributed by volunteers of the American Gut Project.

Along with the rRNA sequences, we have relative abundance data that quantifies the abundance of each observed ASVs in each sample.

Finally, for each sample, we have technical and non-technical metadata. The technical metadata describes information about the technical process of sequencing, such as the processing robot and plating, whereas non-technical metadata contains information about the user. This data comes from forms filled by the participants who produced the stool samples, and contains information about their profile (age category, race, geographical location, etc.), behavior (teeth brushing frequency, exercise frequency, etc.), health conditions (cancer, irritable bowel disorder, crohn’s disease, etc.) and alimentation (types of plants consumed, alcohol frequency, fruit frequency, etc.).

The main focus of the study is the 16S rRNA sequences, but the relative abundance and metadata are leveraged to compute effect sizes to evaluate phylogenies associated with the alignments produced through Borderlands Science.

For more information about the data collection and format, you can refer to the American Gut Project paper: [https://journals.asm.org/doi/10.1128/mSystems.00031-18](https://journals.asm.org/doi/10.1128/mSystems.00031-18)

For more information about sequence alignments and effect sizes results, see Table of Contents.

# 3. Sequence Clustering

Borderlands Science's goal is to align 951,445 RNA genome fragments, sequenced from human gut microbes in stool samples.

Given a dataset of one million sequences, each containing 150 nucleotides, and a puzzle with the dimensions 6x8, there are (1.41 * 10^38) possible puzzle combinations, which would make it extremely difficult to get sufficient coverage from player data to draw conclusions.

Moreover, due to the similarity between some of these sequences,treating them as one million individuals would probably not be the best use of the players' time and energy. To maximize efficiency, we used CD-Hit (Fu et al. 2012) to cluster the set of sequences by similarity, to obtain a set of cluster representatives to build an alignment for the game.

We lightly tuned the default parameters to reduce the size of the initial set by a factor of 50 to 200, to sufficiently reduce the problem to achieve high coverage of player solutions, without reducing it to the point of obtaining clusters that would be too heterogeneous. We obtained about 10,500 clusters.

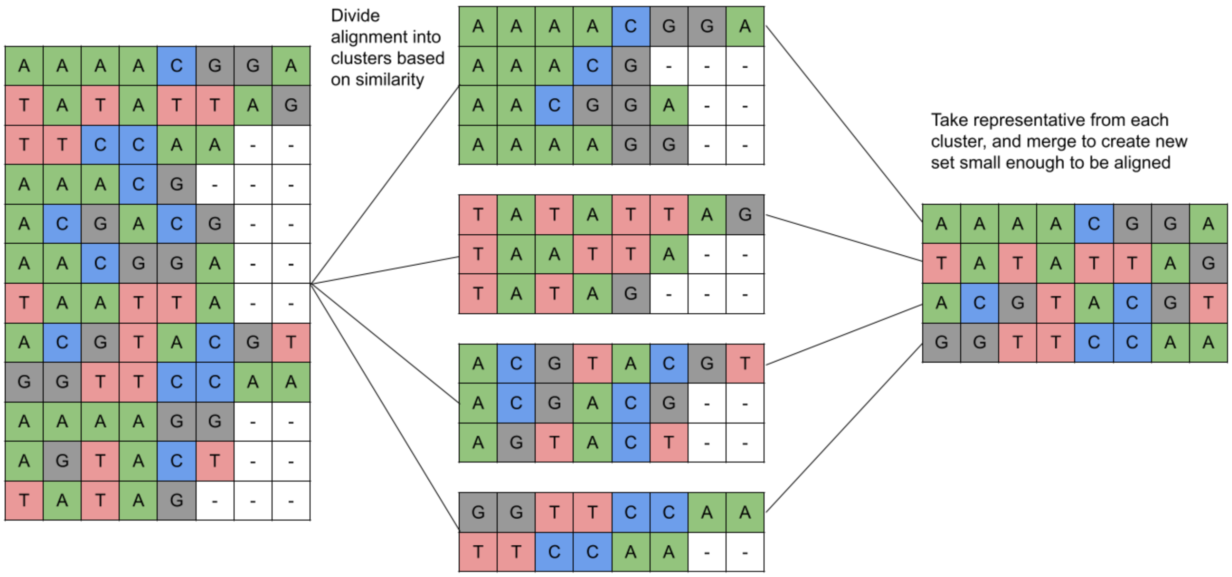


*A visualization of the sequence clustering process*

# 4. Tight PASTA scaffold alignment

Given a large set of sequences, it is unrealistic to expect humans working on small batches without insight on the larger picture to be able to produce a perfect alignment. Additionally, a lot of human time would then be invested in tasks that computers do not need help with (aligning identical subsequences, for instance). It is much simpler to submit an alignment to the human users and ask them to adapt it. Additionally, it has been shown that humans can solve NP-hard problems, but also improve computer solutions to these same problems. (Fu et al. 2012; Holzinger 2016). This concept is often referred to as a human-in-the-loop model. In summary, we use a computer alignment as a template for the puzzle rather than just the sequences to focus the human actions on the most important problem solving steps (the finishing of the alignment).

For game design reasons, it was a lot easier to build an interactive and fun game centered around inserting gaps than one centered around removing them (see Section 6). For that reason, we chose to use PASTA (Siavash Mirarab et al. 2015) to build a template for the alignment. This is because PASTA tends to build tight alignments (alignments with few gaps), even with a high number of sequences.

At the time of generating this PASTA alignment of about 10,500 cluster representatives, we observed that ~900 of these representatives did not align at all with the other sequences. Further investigation revealed these ~900 cluster representatives only accounted for a total of ~4,500 sequences out of the one million set, for an average cluster size of ~4.5, compared to an average cluster size of 95 for the entirety of the set. We concluded that about 4% of sequences that don't align with the rest of the set at all were about in line with the expected rate of sequencing errors, and we removed these cluster representatives from the set.

This left us with a PASTA alignment of 9,667 cluster representatives, representing a total of 946,740 sequences. This alignment was used as the scaffold to generate all the puzzles mentioned in this project.

We also considered alternative alignment methods, such as MAFFT, MUSCLE, or structural alignment software such as ssu-align. MAFFT and MUSCLE were rejected because they produced alignments that were too wide to easily create puzzles (which we later revisited for phase 2, see section 5). As for ssu-align, it could not be used off-the-shelf for our project since it aligns bacteria and archaea separately (as it uses a different structural model for each), and we do not have accurate taxonomic classifications for our sequences.

It should be noted that ssu-align, even when only applied to sequences that it identified as bacteria (so about 9100 of the 9667 cluster representatives), produced an alignment that was wider and achieved a worse compound metric than all other alignment methods listed in Table 1 in the main text. We hypothesize that this is due to the poor taxonomic annotation of the sequences, the shortness of our sequence fragments, and the poor structural signal of the first third and last third of the sequence fragments in our dataset, which make our data not ideally suited for a tool such as ssu-align.

# 5. Puzzleification

The puzzles were generated periodically every month from March 2020 to the end of phase 1 of the project, in August 2021, to make sure players always had new puzzles to play. In order to get a wide enough distribution of solutions, each puzzle was aimed to be played by 45 different players, in three different versions, each with a slightly different number of tokens available, to obtain a slightly wider sample of the score-gaps trade-off space.

The puzzle generation process for phase 1 used a supervised stochastic process: given a range of puzzle sizes required by the players, a list of column ranges is generated so that each column range is used to attempt to make puzzles. Then, for each column range, N sequences (depending on the size of the puzzle being generated) are sampled from the PASTA alignment. In order to compensate for the bias that comes from the gravity effect, puzzles are made both from left to right and from right to left. To make sure the user has room to add gaps, the top two rows of the puzzle are always empty. In practice, most puzzles occupy roughly half the screen at the start of the game. Additionally, in order to add dynamism, each sequence in each puzzle has some probability to be shortened or elongated. The probability of being elongated or shortened by 1 base is around 0.15 on average (depending on the version), and then the probability diminishes for each further base to be added or removed. Bases for elongation are taken from the next columns in the alignment. This lets the player see sequences of different lengths, which allows them reconsider alignment choices that were made in the initial PASTA alignment.

Puzzles are selected or discarded based on test play by a greedy solver, who mimics a low-skill player. The goal of the greedy player is to discard puzzles in which there is little incentive to move bricks because the starting gravity effect already yields a local optimum, determine how much room there is for improvement to assign a number of tokens, and determine the par score the player has to beat in order to get loot, which we can think of as the minimum effort score. Since puzzles that were static were rejected, this naturally led to regions with more room for improvement to be over-represented in puzzles.

Finally, in the objective of making the game more engaging and improving its replayability (and thus generating more data), an offset was introduced between the consensus guides and the sequence featured in some puzzles. An offset of 1 would mean puzzles using sequences from the alignment columns 10 to 14 would have the guides from columns 9 to 15 to enhance the action space for the user, who would then

have to push up many bricks by one to get back to the "par" and, in the process, would have to reevaluate alignment decisions made by the PASTA algorithm.

**Note:** because the puzzles are not in the same orientation as the alignment, the vocabulary can get confusing. For this reason, when we use terms like *rows* or *columns*, assume we are always talking about the game (where each row is an alignment position, and each column is a sequence. To describe elements of the alignments, we will use the terms *sequences*, *tips*, or *fragments* to refer to horizontal coordinates, and the term *position* for vertical coordinates.

*In the following sections, we cover the puzzle generation process step by step from one end of the pipeline to the other.*

## 5.1 Puzzle Overview

### 5.1.1 Rules and Objectives

When a player commences a puzzle they are provided a two-dimensional grid with a set number of columns and rows based on the difficulty. Each column in the grid is a subset of a 16SrRNA ASV. Columns contain an assortment of nucleotides represented by four coloured bricks. Colours are randomly assigned to the nucleotides adenine, cytosine, guanine, and thymine at the start of a puzzle to prevent bias towards a particular colour. A guide is presented alongside the grid, with one to two coloured bricks per row. If a brick in the grid matches one of the guide bricks in the associated row, the total score increases by one, otherwise it is worth zero points. If all bricks in the row match the guide, the score for the row is equal to the number of columns multiplied by 1.15. A player is given a set number of gap bricks, which they can insert in between bricks anywhere in the grid. Once a gap brick is placed, it pushes all proceeding bricks in the column upward by one. Likewise, removing a gap brick causes all proceeding bricks in the column to collapse by one. A brick cannot surpass the boundaries of the guide. The objective of the game is to insert gap bricks into the grid to align with the guide as well as possible and maximize the score.

These are the pre-established rules of the game determined during the initial design in partnership with Gearbox Software, and are not subject to change (see Section 6). The only methods which can be changed are how the puzzles are generated, and how the player solutions are handled.

### 5.1.2 Puzzle Generation Phases

Puzzle generation was broken down into two separate phases during its lifespan. The first phase focused on alignment coverage, attempting to produce puzzles for as many of the sequences and positions as possible. The resulting solutions are the primary focus of our realignment tests. This phase exclusively used PASTA as the base alignment, and incorporated the version one greedy solution algorithm with hard windows, and an acceptance parameter of three greater than the score of the collapsed puzzle. All these methods are discussed in depth in this chapter.

While phase one focused on coverage, the objective of phase two was experimentation. This phase consisted of various methods for puzzle generation, with the goal being to reduce the number of puzzles needed, while still improving final results. All methods listed in this chapter not used during phase one were utilized in phase two.

***Note: all the results mentioned in the main text and the other sections of the supplementary material were obtained from Phase 1 puzzles. Complementary information about Phase 2 is provided for context.***

## 5.2 Puzzle Construction

### 5.2.1 Puzzle Origins

Once a set of representative sequences has been generated, it can be subjected to an alignment algorithm. Due to the size of the alignment file it is unrealistic to expect humans to produce a perfect alignment given small batches without insight on the larger picture. As a result, it is more reasonable to run the set of sequences through an existing alignment algorithm, and utilize that output as the baseline for puzzle production. For our purposes, the alignment file was run through PASTA due to a dense alignment with compact regions of nucleotides ideal for our puzzles. From that point, puzzles are generated from the PASTA alignment, searching for small regions which our algorithms believe can be improved. Further testing also explored using Muscle alignment for comparison, versatility, and flexibility.

### 5.2.2 Puzzle Sizes

Prior to creating any puzzles, grid size must first be determined. Puzzles are defined by the number of sequences, displayed as columns, and number of alignment positions per sequence, displayed as rows. Puzzles also contain an additional empty two rows at the top of the puzzle containing guide information, but no sequence nucleotides. This

ensures that a certain number of gaps can be inserted without surpassing the guide. Borderlands Science utilizes ten difficulties, with a number of sequences between six to nineteen, and columns ranging from seven to twelve.

### 5.2.3 Window Type

When searching regions suitable for player puzzles, a number of consecutive alignment positions must be observed based on difficulty. In order to achieve this, we use a sliding window algorithm, going over every potential region. We developed two different methods to utilize this sliding window, The first being a simple hard window, while the other being a more flexible soft window.

**Hard Window**
The first approach for finding alignment regions for a puzzle is a hard window. Using a sliding window with a size equal to the number of puzzle rows specified, we can obtain a region with subsets of all sequences. While this method does have its benefits, it does have drawbacks.

First, this method is very fast, efficient, and reliable. It allows the puzzle generation to find small dense regions of nucleotides, and determine if they would benefit from inserting, shifting, or removing gaps. Second, this window may include positions containing primarily gaps, providing opportunities to condense the alignment.

The first potential drawback is the existing guide used for solving puzzles. The guide finds the two most common nucleotides (including gaps as nucleotides) and asks the player to attempt to align the puzzle to the guide as well as possible. If a position is 99% gaps, and 1% Adenine (A), the guide will display (-, A). Due to the simplicity, the puzzle will reward a player for aligning an A, yet will not give any incentive for inserting a gap. This causes many regions which the original alignment algorithm determined should be gaps, to collapse further. This is not necessarily bad, but it does impose a bias.

The other larger drawback is the restriction on dense clusters of nucleotides. This is not an issue using an already dense alignment such as PASTA as the base, but does cause difficulties using alignments like MUSCLE which is far more widespread, and may not have enough nucleotides within a defined window size. An array of positions consisting of primarily gaps may be the result of a single sequence, and by ignoring the sequence, those positions would be irrelevant and removed. Since we do not want to impose our own bias, the sequence remains, and the large array of gaps creates a divide preventing hard windows from creating certain puzzles.

**Soft Window**
The second method that has been implemented is a soft window. A sliding window method is used to determine the starting row of the puzzle, rather than the entire puzzle region. From that point the soft window retrieves the next x nucleotides per sequence, rather than the next x positions. This ensures each sequence has a subset of x nucleotides. Once a set of sequences is chosen for the puzzle, we align the sequences based on original alignment positions, and trim to retrieve the first x positions. The end result is a set of sequences which spanx positions, conforming to the requirements of the puzzle, but ignoring positions consisting of only gaps irrelevant to the given sequences. As with hard windows, this method has its perks and quirks.

The primary benefit is that soft windows are better suited for wide-spanning alignments such as MUSCLE, no longer restricted to dense regions of nucleotides, and able to produce puzzles using small clusters by ignoring arrays of gap positions.

This also addresses a feature of hard windows. Soft windows ignore the positions which the original alignment file determined should be filled with gaps. It does not provide incentive to collapse the alignment by removing those positions from the equation. This does impose a different bias, being unable to consider those gapped positions as part of the puzzle, so window type is important based on conditions.

The main drawback of this method is a more complicated and extensive process than the hard window. It does run slower than the hard window method, but still within an acceptable runtime for our purposes.

### 5.2.4 Sequence Selection

After determining a specific window, Borderlands Science randomly selects a single sequence as the core of the puzzle. The remaining sequences are then filtered based on similarity to the core using a rudimentary scoring schema. Matching nucleotides receive a -1, and mismatched nucleotides gain a +2. Sequences that are not identical, yet retain a certain degree of similarity are accepted. The core sequence, along with randomly selected sequences from the filtered results, are then used as the base for the puzzle. If filtering produces too few sequences, or some sequences contain an identical range of nucleotides, then the process restarts with another sequence as the core.

### 5.2.5 Guide

Borderlands Science's predecessor Phylo required players to align all sequences to each other as well as possible using a phylogenetic process. Unfortunately, our set of

microbial rRNA ASVs lack that phylogeny information. In order to overcome this lack of data, a guide is constructed. The guide calculates the two most common nucleotides per position, which is given to the player alongside the sequences. The player's goal is then to align each sequence as closely as possible to the guide.

### 5.2.6 Offset

Offset is a critical component in ensuring puzzle variety, and allowing different approaches to similar puzzles. Offset modifies the guide to not align properly with the corresponding nucleotides. An offset of one would require a gap at the start of every sequence to achieve the same starting point as an identical puzzle with an offset of zero. These types of puzzles result in greater insight of player methodology given two different instances of the same puzzle with a slight variation.

### 5.2.7 Direction

Puzzles generated heavily utilize gravity as part of the game mechanics. Gravity in the game acts as a constant force, pushing all nucleotides towards the bottom row. Puzzles start with all gaps removed. Adding a gap to a sequence pushes all proceeding nucleotides in the column upwards, and removing a gap allows gravity to push the affected nucleotides downwards. Furthermore, while the player scoring scheme does not penalize gaps, we want to minimize the number of gaps added, so the solution algorithms used for evaluation do impose a minor penalty per gap. The scoring scheme penalizes adding gaps at the start of the puzzle, but does not account for gaps after the last nucleotide. As a result, it takes far less effort to align all sequences near the bottom, rather than the top. In order to account for this bias, puzzles can be generated in either direction.

### 5.2.8 Variety

Players require stimulating games. Repeating similar puzzles can feel tedious over time, and result in less engagement. One step in rectifying this is adding a jagged edge to the top of the puzzle. If a puzzle was a simple rectangle every time, puzzles would be rather boring to look at over time, and give limited gaps per column. This is alleviated by implementing a small chance of variation. Each sequence has a 10% chance to shorten or elongate the sequence by a nucleotide, and an exponentially smaller chance for repetitive changes. The product is a slightly jagged edge at the top of the puzzle which provides more visual appeal, yet the sequences are not so different that it hinders the realignment process.

## 5.3 Puzzle Testing

### 5.3.1 Puzzle Base Point

Prior to a player even starting a puzzle, each sequence subset is collapsed, removing all existing gaps from the original alignment. This provides a player free reign to choose how the local alignment should look, whether it will resemble the original alignment, or something else entirely.

### 5.3.2 Puzzle Scoring Scheme

**Player Scoring**

Borderland Science's predecessor Phylo utilized an affine gap cost model for its scoring scheme. This scoring is often used in pairwise alignments, and scores nucleotides accordingly. Match = +1, mismatch = -1, gap opening = -4, gap extension = -1. In order to accommodate a faster paced game, the player scoring scheme was vastly simplified. If a nucleotide matches the provided guide, they receive a point. Otherwise it is worth zero points, and gaps are worth zero. If all nucleotides in a row matched the guide, they receive bonus points equal to 1.15 times the number of columns in the puzzles.

**Solution Algorithm Scoring**

As part of the puzzle generation process, a solution algorithm is run to mimic rudimentary player behaviour, and evaluate if a region can be improved. Despite solving the same puzzle, the puzzle solution algorithm can incorporate different scoring schemes to subject regions to greater criticism. This was done to ensure better puzzles are chosen, and player solutions will have a greater impact. In order to penalize adding gaps, while not straying too far from the player scheme, most solution algorithm scoring imposed a -0.6 for each gap added. Later experiments also used the affine gap penalty cost scoring scheme used in Phylo, due to its previous success.

### 5.3.3 Puzzle Types

In order to determine if a subset of sequences requires player analysis, each puzzle is subjected to a solution algorithm. Each algorithm follows a base logic in an attempt to achieve the best score possible. If the results surpass the acceptance parameters the puzzle is considered worth investigating, and is given to players who will explore the problem with a perspective an algorithm lacks.

**Version 1 Greedy Solution Algorithm**

Originally, puzzles were subjected to a greedy solution algorithm with a slightly different design due to game designs that were still unclear. The greedy algorithm attempts to place, remove, or shift a gap in a position which results in the greatest improvement to the overall score, and repeats this process until the score can no longer be improved. Early plans involved shifting nucleotides one at a time, rather than being able to shift an entire sequence by inserting a gap anywhere in the puzzles. This results in only being able to add a gap brick along the top row of the puzzle on the first turn, and sequential turns allow shifting the gap brick down if the score increases. This design allowed the runtime to be drastically reduced, only needing to consider a handful of possible positions. While this method produced millions of valid puzzles, the initial design deviated from the final plan, and resulted in several potentially beneficial puzzles skipped due to improvements only available within the puzzle, rather than at the top row.

**Version 2 Greedy Solution Algorithm**

While the handicapped greedy discovered many useful puzzles, it did hinder optimizing human contribution by neglecting regions which may prove more value. The revised greedy algorithm dismissed the handicap, observing every possible position to add, remove, or shift a gap providing the greatest increase to the score, until no further improvements can be made. In some cases, the solution found could not be improved upon, thus the objective score given to the players is set at half of what the greedy algorithm was able to achieve. This simple change resulted in puzzle solutions achieving far higher average objective scores than its handicapped version. These raised objective scores provide a greater challenge to the player, and force them to make more well thought out decisions beneficial to our realignment.

The primary hindrance of the revised greedy algorithm is that to evaluate each position in the puzzle for each step, generating thousands of puzzles becomes very costly. In order for this method to be efficient, certain shortcuts can be taken to minimize the necessary work.

The first condition is ignoring areas where adding a gap is impossible, or irrelevant. If a column has already reached the max height of the guide, no more gaps can be added, thus the column can be ignored. Furthermore, if a gap has been added, it is redundant to add a second gap both before and after the existing gap, as it will have the same outcome.

The second step is improving the time required to calculate the score of the puzzle. Rather than looking at each individual nucleotide and determining if it matches the

guide, we can retrieve the sum of all nucleotides in a row which match the guide, and if that sum is equal to the number of columns, we can add the row bonus.

Lastly, the most critical change is reducing the number of times needed to recalculate the score. By calculating the score of the puzzle prior to adding a new gap, called current_score, then adding a gap at the start of a column, recalculating, and calling that future_score, we already have all the information needed for the given column. If you add a gap to row three, you need to make minor adjustments for the individual row, but rows one and two will be equivalent to the rows in current_score, and all rows preceding row three will be equivalent to future_score. This results in only calculating the score once per column, and then each potential gap position in the given column is a simple equation of (current_score < row) + row + (future_score > row). This reduces the number of times calculating the score from n^2 to n+1.

**Profile Alignment Algorithm**

The improved greedy algorithm saw a significant increase in average score, yet it still has one glaring flaw due to the nature of greedy algorithms. Given a puzzle where multiple gaps are needed before seeing a large improvement, a greedy algorithm will ignore it. Thus a second solution algorithm was developed to account for most cases. The profile solution algorithm performs a modified profile alignment on each column against the guide using an affine gap cost model. Each column is aligned as best as possible with the guide, then the total puzzle score is calculated the same way as the greedy alignment. Due to processing each column individually, row bonus points are not factored into the column alignments, but the points are still applied should the final solution produce complete rows.

The modified profile alignment behaves as a standard profile alignment, with only two exceptions. The first being that the guide has up to two possible nucleotides rather than one, therefore it must consider both as valid options. Second, gaps beyond the column are not penalized. Once all nucleotides are matched with a position in the guide, the remaining gaps aligned with the final positions in the guide do not receive a gap penalty.

In order to remain consistent with the greedy algorithm, the pairwise algorithm utilized the same scoring scheme for most puzzles generated. Some puzzles were generated using Phylo's scoring scheme, as the -4 penalty for opening a gap made the greedy algorithm virtually impossible. This scoring schema generated far fewer puzzles, but achieved far higher average scores.

## 5.4 Acceptance Parameters

### 5.4.1 Minimum Score

Early generations of the puzzle generation accepted any puzzle whose solution score had improved by at least three in comparison to the collapsed puzzle score. This was determined as an acceptable degree of improvement for the lowest difficulty puzzles.

Improvements were later implemented to compare the solution score to the score prior to collapsing, as that is an accurate representation of the original alignment. Furthermore, the minimum improvement of three has a decreasing impact as difficulty increases, and average score improves. There is a high degree of correlation between the number of rows and average solution score. We introduced an incremental minimum score improvement, which could raise the minimum acceptance score by a set value, typically 0.5, for every column or row added per difficulty.

### 5.4.2 Minimum Moves

While minimum score ensures puzzles provide solutions with a greater score than their predecessor, a minimum number of moves was also implemented to ensure puzzles remain engaging to the players. If the puzzle is able to surpass the prior alignment score with a single move, it will be a rather short lived and boring experience for the player, and the puzzle itself will not provide much information for realignment. By implementing a minimum number of moves, the puzzles remain engaging, and open up the possibility of various good solutions.

### 5.4.3 Variety

To build upon the minimum moves requirement, a minimum variety was added to ensure a certain number of subsequences were used, rather than only adding gap bricks to a single column. This assures us more of the puzzle information will be relevant, rather than a single subsequence, and will further instill player engagement.

# 6. The Borderlands Science Game

Borderlands Science is a mini-game that is accessible from within the Massively Multiplayer Online game Borderlands 3. In Borderlands 3, players may walk to an arcade machine that allows them to play Borderlands Science. Borderlands Science is a casual arcade game in which the player attempts to align columns with each other to earn enough score to cross a threshold. Score earned by the player can be spent to unlock new rewards such as items and gameplay consumables within Borderlands 3.

In Borderlands Science, the player is shown 7 to 12 columns of bricks, depending on the difficulty of the level. Each column represents a homologous DNA sequence fragment (note that this representation is the transposed version of the typical multiple alignment representation where sequences constitute alignment rows). The number and length of sequences increases with the difficulty level. Each individual brick corresponds to one of the four types of nucleotide bases in DNA, differentiated by colour. Each puzzle in Borderlands Science is made of fragments of microbial 16S ribosomal RNA gene sequences provided by the American Gut Project. In the initial configuration of the puzzle, the bricks are piled up at the bottom of the screen, as though they were under the effect of gravity.

The player is provided with a limited number of gap tokens, which they can insert between bricks to maximize the alignment of bricks against the guides on the left. These guides provide insight about the overall context of the alignment region the sequence fragments come from.

The cost of adding a gap is conveyed to the player as the resistance to the gravity effect. The main challenge of the game is to beat the nominal score set by the naive greedy AI player in the face of limited gap tokens and move on to the next puzzle.

## 6.1 Game Design: adapting Phylo to the Borderlands Universe

Borderlands 3 is a high-intensity, action-packed video game focused on gunfighting, looting and questing. Players are pulled forward by the power growth of their characters and pushed by the storyline to explore new areas. This isn't a natural fit for a puzzle game such as Phylo (Kawrykow et al. 2012), a version of which was planned for Borderlands Science. We had to plan for the best place to lure players into their first taste of this slower game experience. The first question was: where do players learn about this initiative?

### 6.1.1 Discoverability

Borderlands 3 game structure is centered around a clearly identifiable cycle: players go out in the game world to live adventures, acquire new and powerful equipment, complete quests and follow different story arcs. This in-game phase is high-intensity and characterized by a lot of fighting, driving and jumping around. The next phase takes place in their home base which happens to be a spaceship called Sanctuary-3. They may visit their ship to sell unwanted items, upgrade their characters or visit new planets in the universe. There are also many story-driven moments taking players back to their ship. This second phase is low-intensity and allows for players to catch their breath, decide what to do next and jump back into action once they're ready.

It felt natural for us to include the Borderland Science experience as part of that second phase. To push things further, we decided that the minigame would had an actual representation in the game world as a vintage arcade booth. Players had to navigate their ship to reach that arcade machine and directly interact with it. This decision was taken for multiple reasons:

1. An arcade booth is a well-known trope which serve as a clear invitation to take a break and jump in a new experience.
2. Having a new visual element appear in their spaceship would draw the players attention much more than an option appearing solely in menus, it gives Borderlands Science a much greater sense of importance to them.
3. Borderlands 3 art direction is greatly inspired by the 80's playful relationship with technology, the arcade booth helped make Borderlands Science feel like a real part of the in-game world rather than clashing with it.

The arcade booth was positioned within the ship's medical bay headed by the peculiar Dr. Patricia Tannis. This was in part because the theme of the minigame revolved around biological concepts but also because Dr. Tannis were presented as the game's inventor to link it to the game's universe. Players were directed toward the Medical Bay through the usage of a special quest marker which would direct them to the arcade booth while explaining its relationship with the in-game world and its characters.

### 6.1.2 Player engagement

Once players found the arcade machine and played a few rounds of Borderlands Science, we had to convince them to come back and complete some more. Even with the best puzzle-game experience possible, Borderlands players didn't purchase the game to complete puzzles but to shoot and loot. The mini game had to provide enticing reasons for a vast diversity of players.

6.1.3 In-Game boosters

For players that are **achievers**, that care mostly about optimizing their character power and get the best possible equipment in the game, we introduced the notion of Boosters. Completing puzzles within our minigame awarded them with a currency that could be used to purchase in-game Boosters that would greatly speed up their progress. The effect of these boosters ranged from receiving bonus experience points to dealing more damage to enemies but the most popular one boosted the player's "luck", slightly tipping the scales so that they would get rarer and better equipment during regular gameplay. These boosters lasted for a few hours and would require players to return to the arcade booth to complete a few more puzzles.

### 6.1.4 In-Game customization

For social players, reaching milestones in the gameplay would unlock unique visual customization options. These eye-catching costumes served as a reward by themselves, but we also hoped to generate discussions between online players: "Hey! Nice costume, how can you get it?", increasing players' awareness of the minigame.

### 6.1.5 Narrative Integration

We designed the progression in our minigame as a sort of leaderboard populated with beloved Borderlands characters. As players would complete increasingly difficult puzzles, they would beat some of these characters who would then react to this. This was a clever way of further integrating the experience within the Borderlands universe, inject some much-needed humor and give some hints about which characters of this vast universe were the brightest (or dumbest…). Players enjoyed these interactions which motivated them to see who the next character was and how they would interact with them.

### 6.1.6 Love for science

One key driver for player engagement was the sheer novelty of helping science. In our user tests, once players were made aware that solving the puzzles helped science, they all became much more invested and interested. Some of them even said that they didn't even need any sort of rewards: helping science was enough! These findings helped us in the final dressing of the project, we included a lengthy video detailing how the game contributed to the scientific world. This video even became a marketing piece shared on YouTube and other social media.

### 6.1.7 Leveraging downtime

In Borderlands 3, players that join a multiplayer game (with friends or strangers) will often start in their ship before heading into action. Players that would wait for their friends to log-in or to complete their loadouts were given a convenient and fun way to pass time: solving some puzzles!
Now that we had plans for **discoverability** and made sure to have many ways to drive **player engagement**, we now had to focus on the actual gameplay experience.

## 6.2 The Borderlands Science gameplay

Now that we have covered discoverability and engagement drivers for our Borderlands Science minigame, we ought to look at how the minigame itself plays. The original idea came from McGill's own Phylo but it was clear that Phylo couldn't be taken as-is to be included within Borderlands. It needed to be adapted to its new medium.

### 6.2.1 Leveraging downtime

Following the retro Arcade cabinet we developed, the game's overall aesthetics needed to be completely redone to create a unified vision. The usage of vintage video games tropes such as pixel art, upbeat synthetized music and a simplified control scheme also made the game more approachable, make it feel like a toy to be experimented with by players. This hid the underlying complexities of DNA matching.

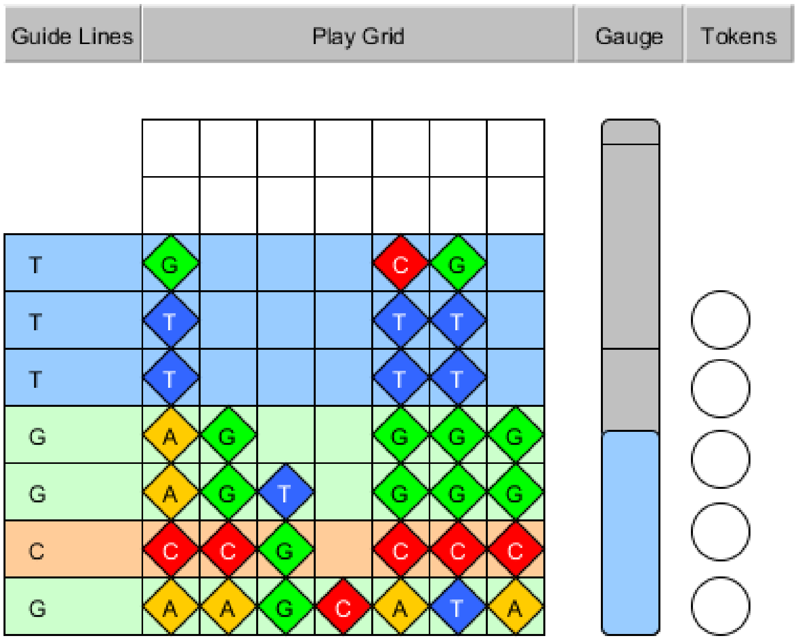


*The very first design mock-up of the game, showing the introduction of **verticality**, **tokens**, **guidelines** and more.*

### 6.2.2 Guidelines and Scoring

In Phylo, players need to align tiles against one another. We simplified this concept for Borderlands Science by providing players with guidelines on the side of the puzzle. Having a tile of the same color of the guide would be worth one point. Having a full line would award a multiplier bonus for the whole line.

### 6.2.3 Progress bar

We illustrated the player's progress with a progress bar. Good moves that aligned more tiles to the guidelines would move the bar forward and bad moves would empty it. If the bar was filled up, the player reached the par score and could move to the next puzzle. However, they were strongly incentivized to find ways of scoring extra points beyond the par-score. The par score was actually based on the imperfect alignment provided by the algorithm.

### 6.2.4 Gravity-enabled, token-based gameplay

One of the earliest changes we brought to Phylo was to rotate their puzzle orientation 90 degrees to make the gameplay vertical. This allowed us to add gravity: pieces would fall from the top of the screen and collapse on top of one another. This dynamic animation brought the minigame's to life, played on the common trope of falling pieces puzzle games (think Candy Crush, Bejeweled, Tetris, etc.) and simplified the control scheme.

Instead of having the rows of pieces left or right using their mouse, players now only had to manage a single direction: up. They did that by inserting a *token* in a column, pushing all the puzzle tiles under it upward. The player could then remove a token to take it back and the pieces would automatically fall back into their original position through gravity. This made the game easy to understand and fun to interact with. This also removed the need of using a mouse to drag and drop the rows sideways, allowing players to use a regular game controller.

### 6.2.5 Limited Tokens, Optimized Puzzles

Finding the best DNA alignment is a two-parameters optimization problem: how good is the alignment vs. How many gaps were added by the user. Balancing these two variables is tricky for a player. At first, we gave the same number of tokens for a given puzzle to all players and incentivized them to reach the best possible alignment by using the smaller number of tokens possible. However, we quickly found out that

players intuitively used all of their tokens, being focused on finding the best alignment possible, and didn't feel good about holding on tokens before submitting their solution.

We solved this in quite an elegant way. We presented the same puzzle to different players with a different number of tokens. This simplified the required task to what players intuitively wanted to do: maximize their score using all tokens at their disposal. Rather than thinking about a single player, we solved a game design issue by looking at the whole community of players. Lifting the complexity of a task from the shoulders of a single player and spreading it across all.

### 6.2.6 Puzzle size

In Phylo, puzzles could take up to 30 minutes to complete. For Borderlands Science, we aimed for a radically shorter time: less than a minute. We achieved this by scaling down the puzzle sizes. Individually, these bite-sized puzzles may not provide a big volume of data but Since we expected a much larger number of players, we felt confident that, in this case, quantity trumped quality.

### 6.2.7 Difficulty Progression

| Difficulty | | | | | | | | |
|---|---|---|---|---|---|---|---|---|
| **Difficulty Rating** | **Character** | **Target Solved Puzzle** | **Max Tiles Width** | **Max Tiles Height** | **Grid Width** | **Grid Height** | **Double Guide Lines** | **Inserted Tokens** |
| **0** | Clap-Trap | 1 | 7 | 6 | 7 | 7 | N/A | N/A |
| **1** | Brick | 10 | 7 | 6 | 7 | 7 | 0? | 0 |
| **2** | Mordecai | 15 | 7 | 7 | 7 | 8 | 0-2? | 0 |
| **3** | Torgue | 20 | 8 | 8 | 8 | 12 | No max | 0 |
| **4** | Marcus | 25 | 10 | 8 | 10 | 12 | No max | 0 |
| **5** | Ellie | 30 | 12 | 8 | 12 | 12 | No max | 0 |
| **6** | Lilith | 35 | 14 | 8 | 16 | 12 | No max | 0-5 |
| **7** | Mad Moxxie | 40 | 16 | 8 | 18 | 12 | No max | 0-5 |
| **8** | Tannis | 50 | 18 | 8 | 20 | 12 | No max | 0-5 |
| **9** | True Tannis | ∞ | 20 | 8 | 20 | 12 | No max | 0-8 |

*The original difficulty progression chart, illustrating the variation of puzzle size and number of double guidelines, these values are not the one that made it into the final product.*

Since we couldn't reliably estimate the difficulty of a puzzle by looking at its composition, we managed the difficulty progression by scaling up the puzzle sizes and number of double-colored guidelines.

### 6.2.8 Tutorial Levels

We hand-crafted five tutorial levels to teach the following skills:

1. Basic token usage and alignment
2. Scoring rules and stacked tiles
3. Optimizing score with imperfect solutions
4. Introducing the double-guide lines.
5. A test of all learned skills using a live puzzle of difficulty "0".

### 6.2.8 Bias elimination in visual elements

We introduced some mechanics to make sure to eliminate unconscious biases in the solutions:

- **Color bias**: every time a puzzle was presented to a player, the color of the tiles and their DNA nucleotide would be randomized.
- **Direction bias**: every time a puzzle was presented to a player, the direction it faced "up" was randomized.

# 7. Puzzle Database

Solutions that players have submitted are saved. Data that is stored includes each action the players make (where they place or remove their bricks) as well as their time stamp. Players also have unique identifiers. Massively Multiplayer Online Science (MMOS) hosts the database of all puzzles and solutions received from players of Borderlands Science, and act as an inbetween between the scientific team and the game. They work in collaboration with Gearbox to make sure the data in the game is always up to date and functional and monitor the technical side of the database, allowing the scientific team to focus on puzzle design and results analysis.

## 7.1 Data Storage and Data Management

The puzzle construction pipeline (see 5.2) produces a lot of data that needs to be delivered to the end user, the gamer. This process includes multiple stages and involves three organizations: McGill University, Massive Multiplayer Online Science (MMOS), and Gearbox Software. While McGill and Gearbox are responsible for the scientific and entertainment aspects of the project respectively, MMOS serves as an intermediate medium. It stores puzzles, efficiently distributes them to millions of players and collects player solutions.

First, a newly created puzzle originates in McGill servers, where it gets saved into the internal database along with a massive amount of metadata. Further in the pipeline, additional information will allow researchers to correlate puzzles and solutions with scientific data.

Next, essential information about puzzles is packed into JSON files that are thoroughly tested and validated. Then, they are transferred to the AWS S3  bucket, managed by MMOS.

On the transfer completion, the McGill data processing pipeline triggers the MMOS API. It parses and saves individual puzzles into its internal high-performance distributed database. To serve puzzles to thousands of Gearbox game clients (players) at a time, it uses caching techniques setting aside a limited number of puzzles to run through the players.

Finally, the Gearbox game client (Borderlands Science Arcade Booth) requests individual puzzle data from MMOS servers. In return, it provides a player solution with a player identifier (anonymized) that is posted back to MMOS servers. Player solutions

are saved into MMOS' internal database, aggregated into archives, and stored in AWS S3 service. In turn, the McGill data processing pipeline transfers them back to McGill servers, associates solutions with science data using metadata, and saves them in the internal database ready for further data analysis.

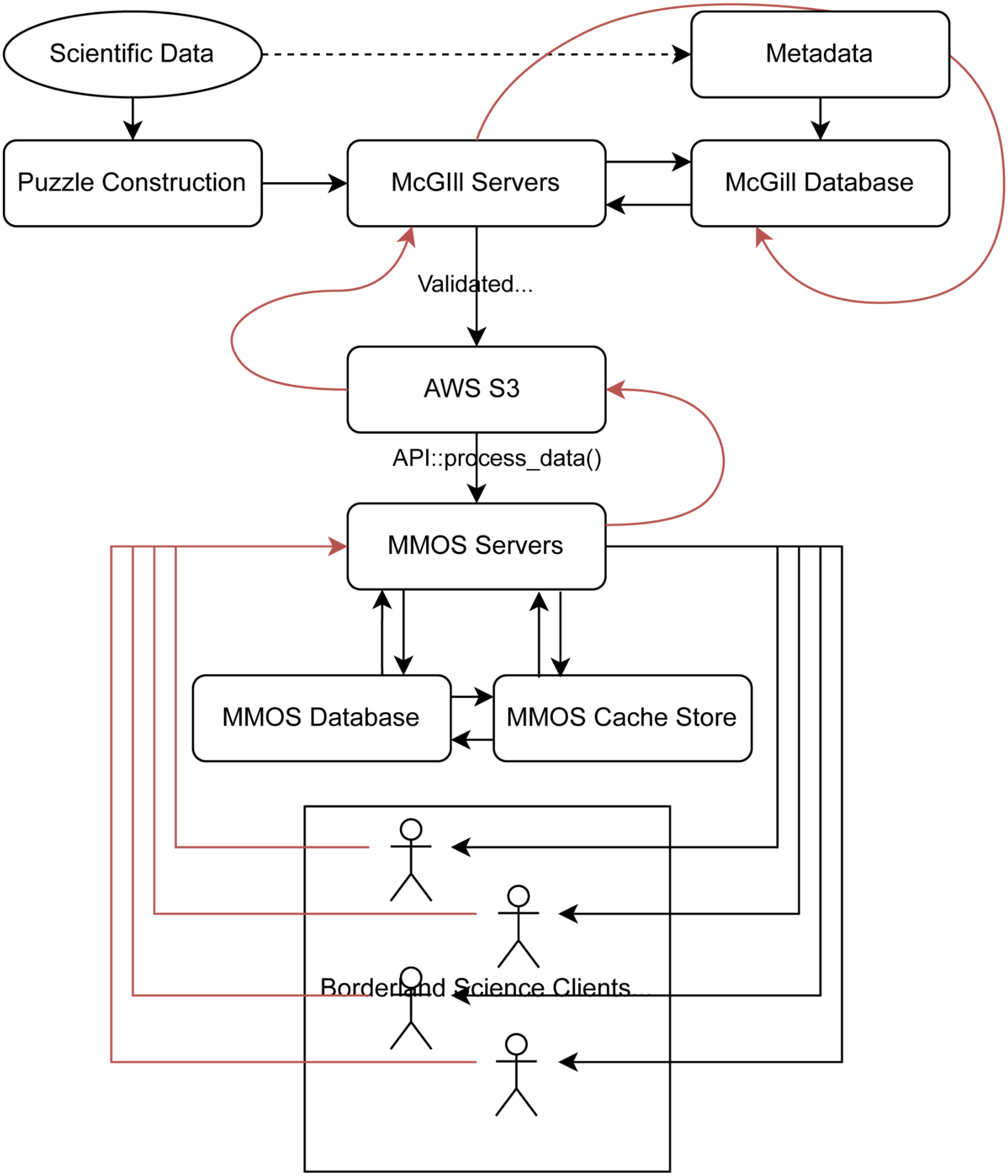


*A summary of the Borderlands Science database. Black lines represent a flow of puzzle data, red lines show a flow of solution data.*

# 8.Solution Filtering

Not all solutions are equal, even though they are all useful. As Borderlands Science puzzles are played by an average of 45 players each, some filtering is needed to remove the noise from the solutions.

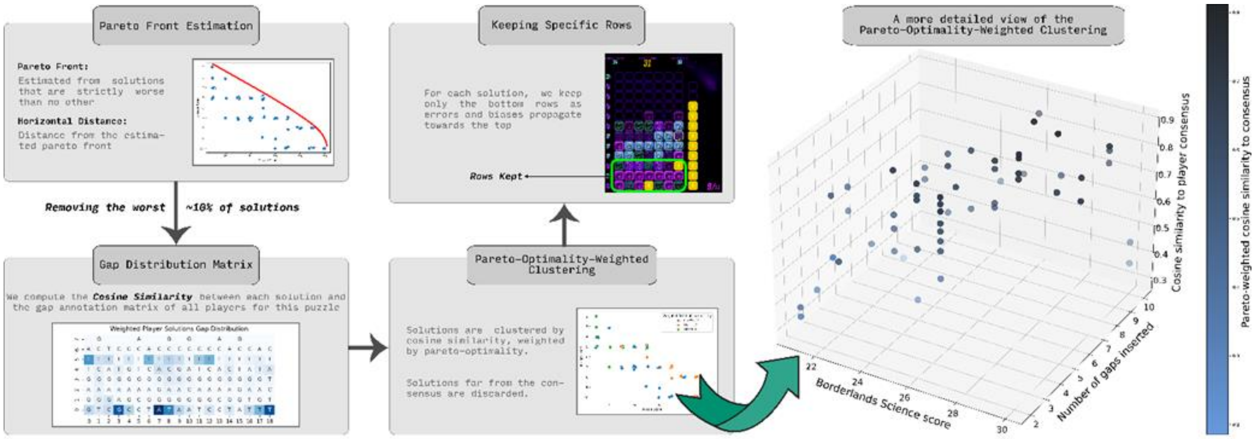


***Solution filtering***. *The player solutions are jointly filtered by pareto-optimality and consensus. The ~20-40% darkest dots on the three-dimensional plot would be selected as high-confidence puzzle solutions and used in the re-alignment pipeline*

## 8.1 Filtering by Pareto Optimality

There is an assumed Pareto optimality of the solutions of a puzzle where the solutions have the best puzzle score for the fewest amount of gaps added. We examine the effects of filtering using pareto optimality by keeping only the submitted solutions that are closest to an estimated pareto front created by all solutions of each unique puzzle.

### 8.1.1 Pareto Frontier Estimation

Estimation of the Pareto frontier is done by selecting 3 unique solutions at the following conditions: The solution with the best score with the least amount of gaps, the solution with the least amount of gaps with the best score, and the solution with a number of gaps equal to the median amount of gaps with the best score.

The amount of gaps used and score of these three solutions are used to solve and fit a parabola of the form y = a(x+b)^2) + c, which represents our pareto frontier in the positive-x and positive-y quadrant.

A proportional horizontal Pareto distance is described by the following equation:

(score - min_score) / (paretoFrontScore - min_score)

Where min_score is the minimum score of any solution within the family, and paretoFrontScore is the score of the quadratic representing our estimated pareto front with the current solution's amount of gaps. Therefore, the proportional horizontal Pareto distance represents a ratio of how close a solution is to the Pareto frontier in context of all other solutions.

## 8.2 Filtering by Gap Distribution Similarity

The variability of solutions may add noise to the potential distribution space and hinder the realignment process. In order to extract specific solutions and strategies, we create a distribution of the gap positions of all solutions and select the solutions that are most similar to that distribution - hence keeping only solutions that most conform to a consensus of gaps that are most often used by players.

### 8.2.1 Calculating Solution Gap Distribution

For each puzzle, each solution's gap positions are added to a distribution matrix, which is subsequently normalized between least amount of gaps added and most amount of gaps added. The distribution similarity score of each solution is calculated using cosine similarity to the distribution matrix.

### 8.2.2 Pareto - Weighted Gap Distribution Similarity

A distribution matrix weighted by each solution's distance to the pareto frontier is created to determine solutions that are similar to the consensus of gaps that decrease the distance to the pareto frontier the most. This is created by weighting the added gap positions to the distribution matrix by multiplying with the proportional horizontal pareto distance before normalizing the full distribution matrix. Filtering is similarly done by selecting ranges of similarity.

### 8.2.3 Removing Least Pareto-Optimal Solutions

To additionally incline the distribution of gap positions towards a consensus of gap positions used by solutions close to the pareto frontier, we remove an amount of solutions with the smallest proportional horizontal pareto distance before creating the weighted distribution matrix of gap positions. The main results remove the four least pareto-optimal solutions.

## 8.3 Minimum Solution Count Per Puzzle

Puzzles with fewer than 15 submitted player solutions are removed as they have too few solutions to generate a meaningful Pareto frontier and distribution for the remainder of the filtering and analysis.

## 8.4 Discarding Upper Rows

Only information about the bottom rows are kept when processing solutions for realignment (3 for puzzles with 8 rows, and 5 for puzzles with 12 rows). This is done by design of the puzzles due to the so-called “gravity effect” which encourages players to align the sequences from the bottom first.

## 8.5 Filtering Experiments and Parameter Tuning

Several experiments for filtering were done to confirm certain hypotheses about the dataset, and to search the parameter space and tune for the optimal set of parameters to generate the best dataset for our realignment pipeline. A compound metric was created to compare the score of the various experiments. This compound metric is calculated with the following equation:

$$(20*KC) + (0.5\ T) / 1000$$

Where KC is the Kendall-Colijn distance of the realignment, and T is the Triplet distance of the realignment. The compound metric represents a better alignment the closer it is to 0.

### 8.5.1 Weighted Gap Distribution Similarity Experiments

We tested a range of filters for the weighted distribution similarity ( 0.5, 0.6, 0.65, 0.7, 0.75, 0.9). The results suggest that a filter to discard solutions below 0.7 weighted distribution similarity yields the best compound metric score. This filter of the data also retains approximately 34% of all solutions, which is acceptable for our purposes. A larger filter will remove too many solutions and make the realignment too variable, thus increasing the compound metric score.

### 8.5.2 Removing X Least Pareto-Optimal Solutions

With the above filter for weighted distribution similarity, we tested removing several amounts of least pareto optimal solutions (2, 4, 6, 8, 10, 12). The compound metric for removing 4 least pareto optimal solutions per puzzle vastly improved upon other amounts. As such we have chosen to remove 4 for our main results.

### 8.5.3 Distribution Weights Experiment

Combined with the above two filters, we experimented with using different weights for creating the weighted distribution matrix - a realignment with the solutions filtered with the full weights of the proportional horizontal distance of each solution, a realignment with half-weighted distribution matrices, and one without any weighting. The hypothesis is that the more weighted the distribution is, the better the compound metric, since the filter will be discarding more solutions that are not pareto optimal. The results appear to support this statement, as the fully weighted distributions produce the best compound metric.

### 8.5.4 Offsets Experiments

Each puzzle was created with a few variations to help increase the variability of the player solutions. An offset was sometimes introduced between the consensus guides and the sequence featured in some puzzles. Adding solutions from the offset puzzles to the dataset decreases the compound metric of the realignment, due to increase of variability in the gap distribution. However, by increasing the filtering of offset-ed puzzles specifically, we can reach a middle point between extracting information from these solutions and the increase in variability. Our tests suggest that a filter of 0.8 weighted distribution for offset solutions only yields the best compound metric.

We observed that these offset puzzles showed an increased average number of moves played for eight of the nine difficulty levels, which demonstrates they add complexity compared to regular puzzles. However, when computing an improved alignment with the same parameters, we observed rather unimpressive phylogeny metrics, with a mean Kendall-Colijn distance of 1,617 and a mean Triplet distance of 69,964. Nevertheless, raising the weighted distribution similarity threshold to account for the increased heterogeneity of the data yields improved results, with a mean Kendall-Colijn distance of 1,240 and a mean Triplet distance of 59,750. While these results are slightly worse than those observed on the data excluding offset puzzles,they are quite close. They also show improved effect sizes on important variables.

For now, we are leaving the exploration of these offset puzzles to future work, because while we believe there is potential to be unearthed from them, we believe the most systematic approach is to clearly establish the contribution of “regular” puzzles and then build on these findings to integrate the more heterogeneous data.

### 8.5.5 Coverage experiments

A key idea in the Borderlands project is in the idea that the vast amount of player solutions will help improve the realignment process by generating stronger consequences of solutions. We have experimented with discarding certain percentages of puzzles at random from the dataset to determine if more solutions generates better realignments. (10%, 30%, 50%, 70%, 90%). This has been determined to be true as removing larger percentages of solutions corellates to a worse compound metric for realignment.

## 8.6 Borderlands Science main result filtering strategy

For each puzzle, the 20 to 60 (objective 45, real mean 43.4) user solutions were filtered by their similarity to the distribution of gap positions from all submitted solutions to that puzzle. This distribution was additionally weighted by Pareto-optimality . Solutions showing a cosine similarity less than 0.7 from this weighted distribution are discarded, with this floor raised to 0.8 for data that includes guide offsets, more heterogeneous. This respectively excludes approximately 62.8% and 77.3% of solutions which are the furthest from the player consensus and pareto optimality. We refer to the remainder as high-quality solutions.

## 8.7 Expert experiments

Not all solutions are created the same, some solutions are submitted by players that have just started playing, and others by players that have been playing hundreds of puzzles.The assumption is that experts that have played the game more often will become better at the game and submit solutions that contain learnt strategies that will be more beneficial for realignment. Expert solutions are defined as solutions that have been submitted by the top 10% of players who have submitted the most solutions. We test removing all expert solutions, as well as removing an equal amount of random solutions from the same puzzles as expert solutions. Removing expert solutions gives us a compound metric of 50.0, while removing random solutions yields a compound

metric of 43.9. By dividing the two, we get the 14% drop in evaluation metrics, supporting our hypothesis. It should be noted that despite this drop, BLS still outperforms all benchmarks.

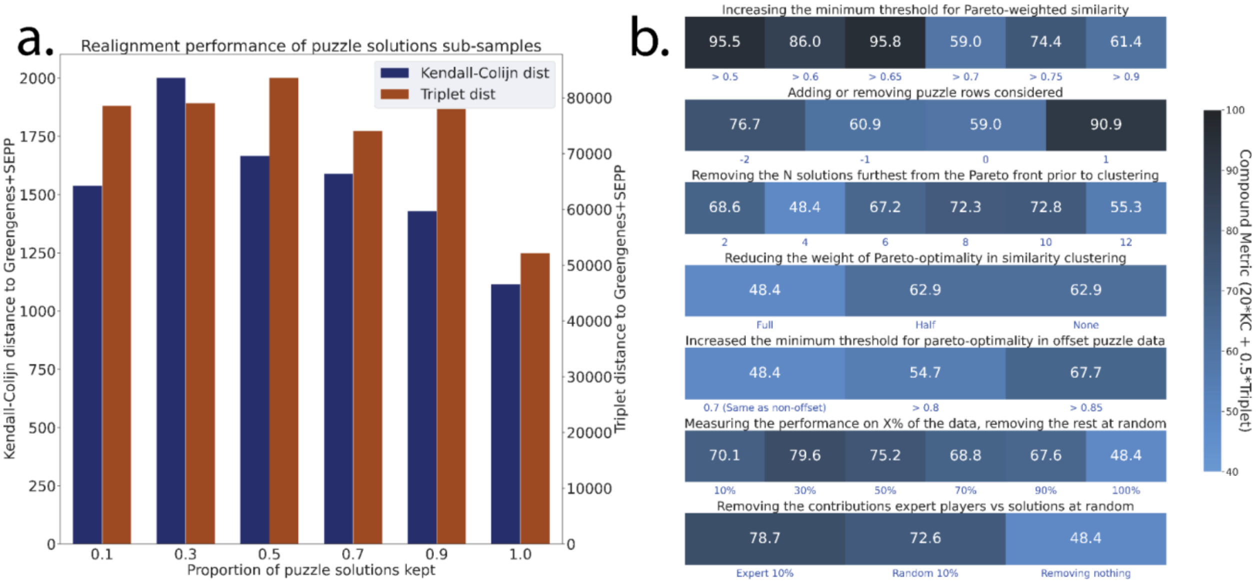


*Variations in the phylogeny performance for different filtering variations. In **a**, we show the results associated with less data, and in **b**, we show the impact of changing various hyperparameters in the filtering process. Note that due to the heavy computational cost of rerunning everything, the experiments presented in a were done with limited sampling.*

# 9. Sequence Consensus

## 9.1 Aggregation of puzzle solutions

Before being passed to the realignment pipeline, puzzle solutions are processed and inserted into a dataframe which we refer to as *mapped_guide*. The mapped guide's objective is to keep track of each nucleotide's positions for each sequence after being processed. It does not refer to the original sequence, or the guide. For the solutions corresponding to a puzzle with an offset, guide positions are adjusted - either by shifting left or right. If a single solution has an 'A' in column 50 of sequence 1234, it will record the occurrence. Thus, for every column, of every sequence, there is a dictionary resembling {A: 0, C: 0, G: 0, T: 0, -: 0} which keeps track of all occurrences across all solutions.

The *mapped_guide* is then processed and put in a 3-layer dictionary data structure. Each key of the first dictionary is a sequence ID, in the second dictionary the keys are positions, and in the third one the keys represent nucleotides, with final values being the count of the corresponding nucleotide in the corresponding position of the corresponding sequence. For each sequence, a range of positions corresponding to a substring of that sequence is used for the next steps; the range being from the smallest position with solution information up to the highest position with solution information (tails from both ends with no solution information are discarded). For positions inside this range that don't have puzzle solution information available, a consensus from the PASTA alignment is used. Additionally, at each position optional extra information based on the secondary-structure (RFAM alignment consensus) can be added too.

```
{sequence_id_1:
    {pos_1: {'A': 100, 'C': 50, 'G': 25, 'T': 75, '-': 150},
     pos_2: {'A': ..., 'C': ..., 'G': ..., 'T': ..., '-': ...},
     ...
     pos_n: {'A': ..., 'C': ..., 'G': ..., 'T': ..., '-': ...}
    },
sequence_id_2:
    {
    ...
    },
    ...
}
```

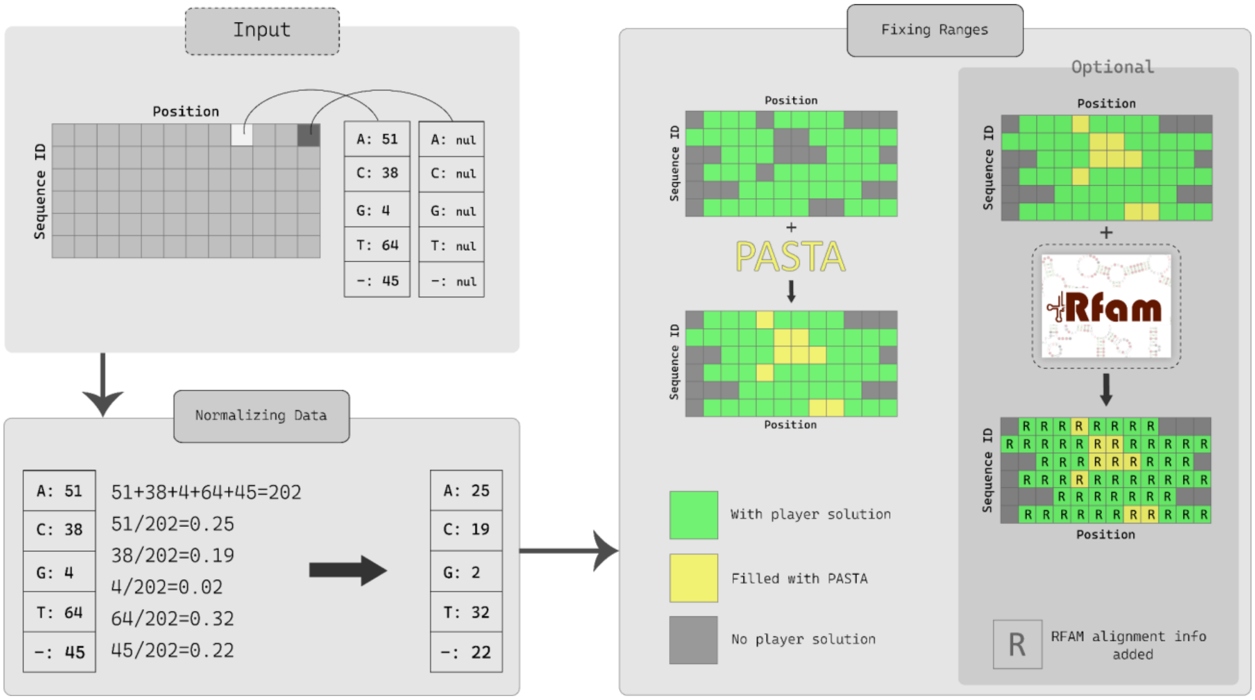


*Processed puzzle solutions are prepared for use in the realignment pipeline at the first step of it: nucleotide counts are normalized, consensus ranges are determined and slots with no information are filled with default (PASTA), and optionally structural information is added.*

### 9.1.1 Normalization

The count of nucleotides is normalized to prevent bias towards regions which received a higher number of solutions. For example, if two positions have the same dominant nucleotide A with 90% of the solutions contributing to it, but the number of solutions attributing the first position is 10,000 while the second one only gets a count of 100. In this situation, without normalization, the sequence alignment algorithms almost always favor matching a nucleotide with the first position (score = 10,000 vs score = 100) even if it creates a rippling effect of mismatches in the entire sequence. With normalized data however, both positions get the same score (score = 0.9): each position’s nucleotide

counts are divided by their sum, giving us a normalization of the count.

```
{sequence_id:
    {pos_1: {'A': 25.0, 'C': 12.5, 'G': 6.25, 'T': 18.75, '-': 37.5},
     pos_2: {'A': ..., 'C': ..., 'G': ..., 'T': ..., '-': ...},
     ...
     pos_n: {'A': ..., 'C': ..., 'G': ..., 'T': ..., '-': ...}
    },
sequence_id_2:
    {
    ...
    },
    ...
}
```

## 9.2 Recreating sequences from puzzle solutions

This step is performed on each sequence individually. Each sequence's nucleotide positions' information is separated and then used to align the sequence. The resulting alignment between the sequence and the information extracted from the puzzle solutions is referred to as the *realignment.*

### 9.2.1 Aligning the sequence with the puzzle information profile (votes array)

Our first approach was to compute a realignment using a voting system. We consider each annotation from each puzzle solved by each player as a vote. In our old method, we used a combination of dynamic programming and backtracking to choose the position of gaps and nucleotides in a sequence in a manner that would maximize the alignment score between the resulting sequence and the votes. One of the shortcomings of this approach was that due to its time complexity, it limited us to short segments of the sequences (of length <15). We then needed an extra step to merge these segments into full sequences. The short segment length and the merging step added unwanted biases into our pipeline that would lead to the final MSA not having the desired quality.

To improve upon the described method, the idea was to enable manipulation of the sequence at its full length. We achieved this with a compromise between Needleman-Wunsch and the voting system. In our new method, we first remove all of the gaps in a sequence. We then align the no-gap-sequence and a list of dictionaries - which we refer to as the votes array - together, optimizing the following function:

$$D(i,j) = \max \begin{cases} D(i-1, j-1) + votes(s_{i-1}, v_{j-1}) \\ D(i, j-1) + votes(-, v_{j-1}) \\ D(i-1, j) - 25 \end{cases}$$

where ***i*** and ***j*** are indexes in subsequence ***s*** and votes array ***v*** which is a vector of dictionaries of votes (for example: v_{47}= {A: 40, C:25, G: 10, T: 10, -: 15} where **-** is a gap). At each step, the algorithm makes a choice between:

- Assigning the current initial sequence position (held by the vector of votes) to the current realigned sequence position (which receives a score corresponding to the vote for that base at that position)
- Adding a gap to the sequence (a scenario which is already considered in the vote)
- Adding a gap into the vector of votes. A gap in this context indicates that we decide to not use the dictionary of votes in that position, because it would lead to a better alignment if matched with a farther position . We tried different penalties for this choice, and because votes are normalized to be out of 100, intuitively -25 was a good choice which was further solidified by our experiments.

The logic behind this algorithm is that the players are suggesting gaps to add, and we are determining how to adjust the original sequence in a way that is most compatible with player opinions.

(See figure below)

# 10. Puzzle-informed Realignment

We have tried to keep our approach towards realignment a simple yet effective one, in order to put more emphasis on the contextual meanings of the players' decisions. The annotations are irregular in coverage and include holes, and we start the realignment pipeline by addressing these issues in the context of preparing our input (See figure in 9.1). We proceed by using the annotations to modify each sequence, creating a MSA, and finally performing post processings to polish our produced alignment.

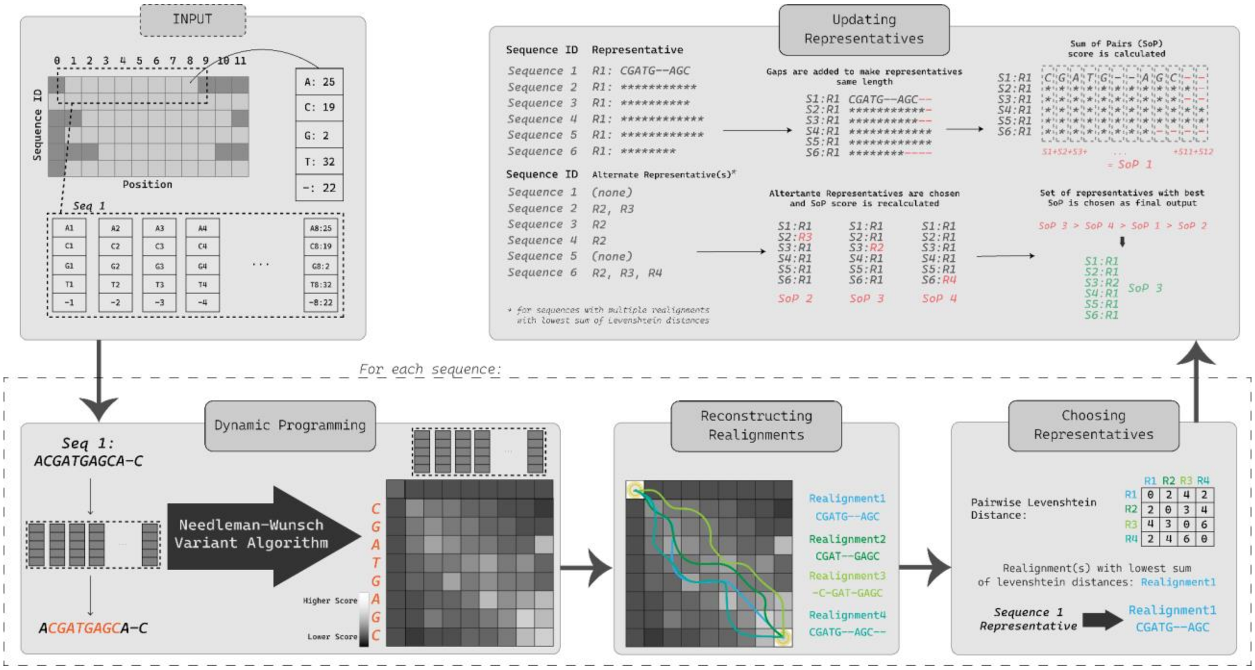


*Depiction of Puzzle informed realignment: each sequence is aligned with corresponding data. Among optimal alignments, the one closer to all others is chosen as the representative for that sequence; in case of ties, alternative representatives are weakly aligned with other sequences' representatives and a choice is made based on Sum of Pairs score of the weak alignment of representatives.*

## 10.1 Alignment Reconstruction

Backtracking is performed identically to the Needleman-Wunsch (NW) algorithm's backtracking (Needleman and Wunsch 1989). The pointers for the argmax of the above optimization are recorded inside a 2D array called *path* with the same shape and size as the dynamic programming table with a list for each entry. When backtracking, the realignment is reconstructed by looking at the pointer in the *path* array (or choosing one of them based on some criteria), starting from the last cell (usually the bottom right which represents the full alignment of the subsequence with the votes array).

In our experiments, a noticeable number of entries in the *path* array tend to have more than one argmax. We have observed that there are numerous possible reconstructions

of the realignment available when backtracking the dynamic programming table for alignment reconstruction. Therefore, we have explored 3 different backtracking ideas.

### 10.1.1 Path order preference

In this backtracking mode, a preference of the pointers to be chosen is provided as an input, and in the backtracking process if an entry of the path array has more than one direction pointers (equal scores in both directions), they are chosen based on the preference provided.

### 10.1.2 Sampling and Consensus

Our next idea for backtracking was to reconstruct all of the realignments. Initially, we had many possible realignments, but reconstruction of all possibilities was not feasible due to the very long run time of the related algorithms. Therefore, we decided to use sampling to reconstruct some of the possible realignments. To achieve this, we ensured each realignment has a uniform probability of being sampled. Realignments are then sampled based on a modified version of the original NW reconstruction, which randomly chooses one of the many possible paths.

After further investigations of the optimization objective, adding a penalty for gap insertions in the votes array, and filling in the empty positions with PASTA information, the number of possible realignments decreased to a degree that enabled us to reconstruct all of the possible realignments; to which we refer to as the *consensus* reconstruction method. The consensus method has been our standard approach for reconstruction since its conception.

### 10.1.3 Representative choices and adjustments

Given that a sequence can produce multiple valid realignments, we need to choose one of them to be the final representation. Therefore, we construct a *Levenshtein distance (Young, Faris, and Armogida 2021)* matrix from the list of realignments, calculating the relative distance of each realignment to others. A low Levenshtein distance indicates that two realignments are very similar, thus the realignment with the lowest matrix row sum acts as a representative which most resembles all other possible realignments. This process is repeated for each individual sequence.

Despite the further refinement by choosing representatives, we may confront cases where there are multiple viable representatives. If there are only two realignments, they

will share the same sum Levenshtein distance. Our final method for selecting realignments is optimizing sum-of-pairs, which is the sum of the alignment scores for each pair of sequences in the MSA. Taking a random representative from each sequence, we construct a new base alignment, and calculate the sum-of-pairs score. Proceeding this, a random sequence with more than one valid option replaces its current representative with another. Sum-of-pairs is calculated again, and if the score improves, the new representative is kept. This repeats for each sequence, and the full process is repeated 5 times. Ideally we would consider every possible combination of representatives for every sequence, yet if only a third of the sequences had a single alternative representative, that would yield 2^3333 combinations, which simply isn't feasible. Despite the seemingly random approach, this method proves to be very effective at getting consistent results.

Another idea we have applied to increase the coverage of the realignments is to allow for flexibility when choosing the representative. This is done by giving leniency to allow realignments with a sum Levenshtein distance within a certain range of optimality to also be included as representatives.

## 10.2 Aligning the sequences together to form a MSA

Because sequences are realigned individually, final realigned sequences have uneven lengths (which does not constitute an alignment), Therefore, to create a well defined Multiple Sequence Alignment, first we tried the Length-based MSA construction method, and later on it was replaced by the Progressive Alignment method.

### 10.2.1 Length-based MSA construction

Originally, we corrected the varying sequence lengths using a growing profile alignment. The sequences were sorted based on their length from longest to shortest, processed in order, and added to a single profile. Gaps were added to each sequence to ensure the sequence is the same length as the longest sequence, using a profile alignment. This would align the profile of the set of previously aligned sequences, with the target sequence, and the resulting aligned sequence would be added to the profile at the end. This process would repeat until all sequences were the same length. While a quick and effective method, we realized this caused an unhealthy bias towards the longest sequence, causing two similar sequences to possibly align poorly based on what was already dictated by the profile.

$$D(i,j) = \max \begin{cases} D(i-1, j-1) + votes(s_{i-1}, v_{j-1}) \\ D(i, j-1) + votes(-, v_{j-1}) \end{cases}$$

## 10.2.2 Progressive Alignment

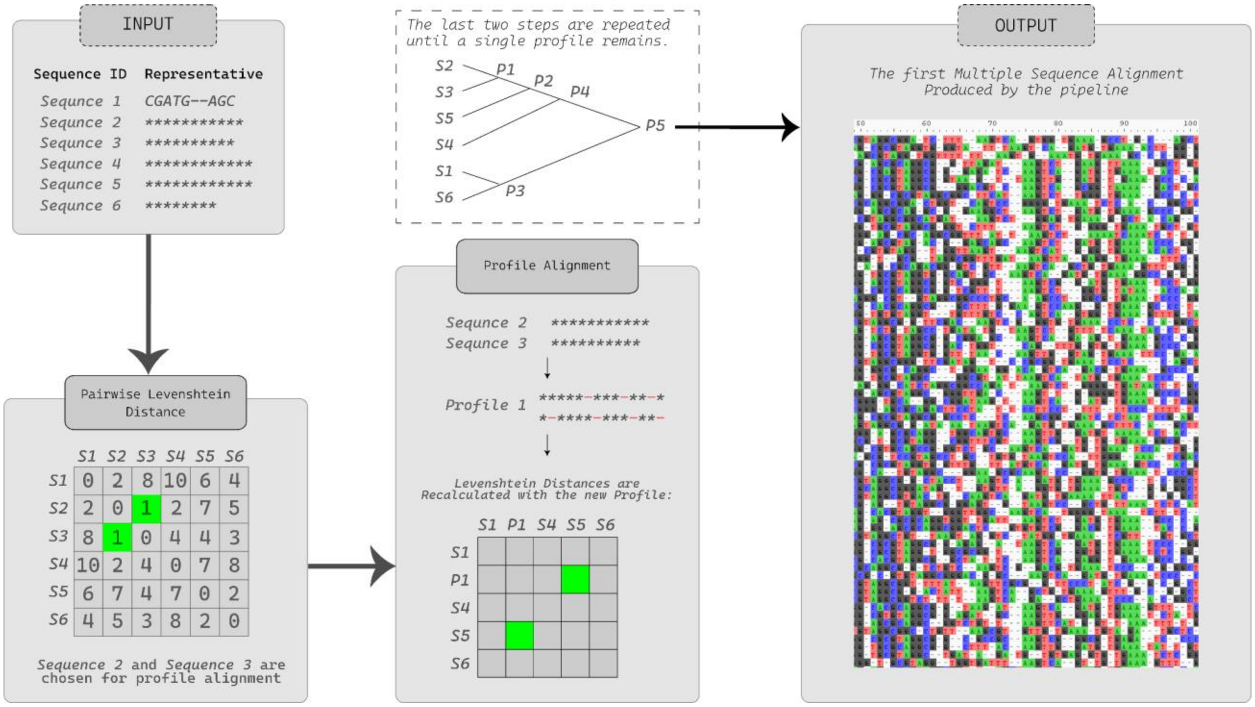


*Representatives are aligned together using a Profile Alignment Algorithm. The result is an unrefined Multiple Sequence Alignment.*

We introduced a progressive alignment to address the bias produced from the Length-based MSA construction, and to ensure gaps are placed in the ideal positions. The progressive alignment finds the two most similar sequences, and aligns them to ensure they are the same length. Once they are aligned, they create a profile which will be expanded on below. Decisions are made using a combination of standard Levenshtein distance, and an alternative Levenshtein distance designed for profiles.

The distance between every sequence is calculated using standard Levenshtein distance to construct a Levenshtein distance matrix. We also experimented with hamming distance with mixed results. The lowest point in the matrix reveals the two closest sequences, which are then aligned. If they are the same length, they are simply grouped together, if not, the shorter sequence is subjected to a profile alignment. Once

two sequences are the same length, they form a profile. The newly formed profile then calculates its distance from every other sequence. If neither sequence was changed, then the distance from the profile to another sequence is the sum of the distance from each individual sequence in the profile halved. However, if a sequence has been adjusted, then distance must be recalculated.

Typically, Levenshtein measures the distance between two strings, in this case, sequences. Normally if two characters are identical they are worth 0, otherwise 1, yet a profile contains two or more sequences which introduces more variables. Our modified Levenshtein calculation utilizes a profile alignment, attempting to optimize the following function:

$$D(i,j) = min \begin{cases} D(i-1, j-1) + (1 - \alpha_i \cdot \beta_j) \\ D(i-1, j) + 1 \end{cases}$$

where alpha_i and beta_j are the normalized vectors containing the count of nucleotides for the given column. A single sequence may have the nucleotide 'A' for a given column, while a profile may have three nucleotides ('A', 'C', 'C'). Thus, the distance for the individual column would be 1 - (1*0.33 + 0*0.66) = 1 - 0.33 = 0.66

Now that we have established a method for calculating the Levenshtein distance of a profile, we calculate the distance to every remaining sequence. We remove the two sequences used to create the profile from the Levenshtein distance matrix, and insert a single new instance for the profile. The process is then repeated, selecting the pair with the lowest Levenshtein distance, and combining them to create a profile. For each iteration the distance matrix is reduced by one, gradually grouping sequences based on similarity. Eventually profiles will be merged together. Should a profile be subjected to a profile alignment, adding a gap to a column adds a gap to every sequence in the profile. After several iterations, the final result is a single profile containing all sequences at the same length.

## 10.3 Postprocessing

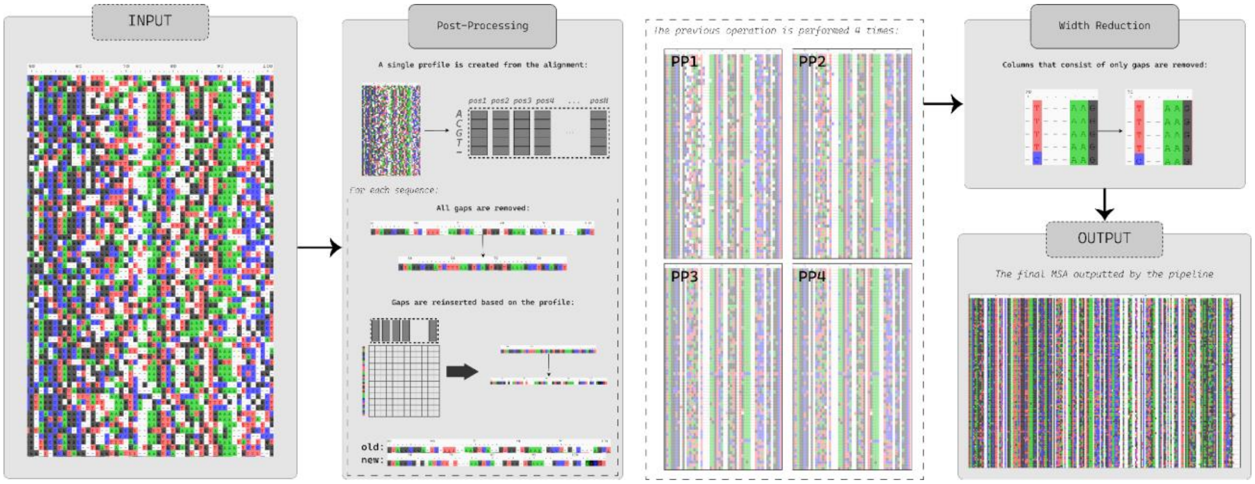


*The unrefined MSA at the end of the Progressive Alignment step is then subjected to 'Postprocessing': Each sequence is taken out of the alignment, has its gaps removed and reinserted based on the profile of the alignment without that sequence. After a few iterations, the full alignment becomes more homogenous and is outputted, and the realignment pipeline concludes.*

Now that we have a valid MSA, there are some undesired artifacts. These artifacts consist of mostly off-by-one errors where we see, for example, half the sequences with two consecutive characters being *gap, A*, and the other half having *A, gap* for the same two columns.

Our postprocessing algorithm calculates the normalized distribution of nucleotides per column to construct a consensus. Each individual sequence is then stripped of all gaps, and undergoes a profile alignment to the consensus which assists synchronizing sequences and aligning gap regions. This process cycles four times, as we saw results start to stabilize after that, and then columns that are only gaps are removed. This post-processing can also be performed on an alignment with no player solutions, which improved upon Pasta on every observed metric, and yielded a new objective for us to surpass with player solutions.

# 11. A game changer for citizen science

While Borderlands Science is building on the innovation and success of groundbreaking first generation citizen science games such as Foldit (Cooper et al. 2010), EteRNA (Lee et al. 2014), Eyewire (Raposo 2015), Phylo (Kawrykow et al. 2012), and standing on the shoulders of more recent game-embedded projects such as Project Discovery (Leifsson and Bjarkason 2015), it is fundamentally different from its predecessors. The number of participants were retrieved from online news articles, which tended to provide more recent/up to date numbers than academic articles. Please note that we tried to find the public number closest to three years after release, but we had to approximate in some cases.

- https://biobeat.nigms.nih.gov/2014/07/meet-rhiju-das/
- https://techcrunch.com/2015/05/01/eyewire-is-making-neuroscience-research-cool-again/
- https://www.redbull.com/int-en/eve-online-project-discovery-interview
- Digre, A. and C. Lindskog, *The Human Protein Atlas-Spatial localization of the human proteome in health and disease.* Protein Sci., 2021. **30**(1): p. 218-233.
- https://www.ucl.ac.uk/news/2019/apr/mobile-game-can-detect-alzheimers-risk

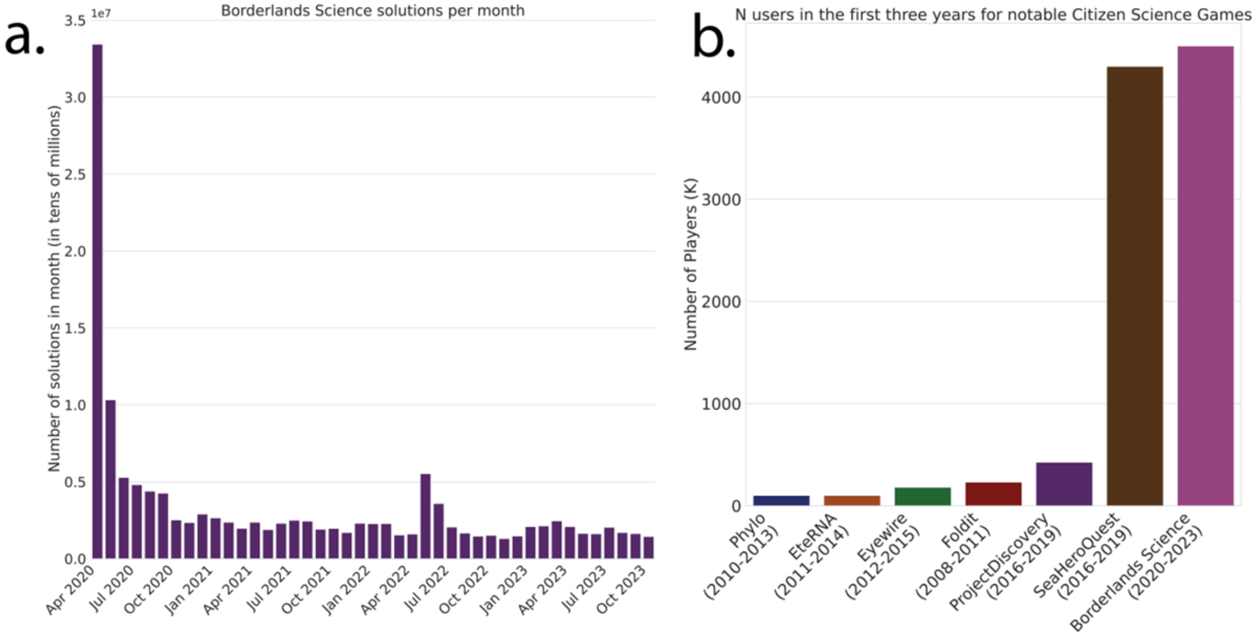


*Monthly participation in Borderlands Science (phase 1), and comparison of player engagement in the first three years of some well-known citizen science games.*

## 11.1 Finding citizen science participants in their natural habitat

The difference in number of players in the first three years between citizen science games (CSGs) that are either embedded in a commercial game (Project Discovery, Borderlands Science), or designed and marketed as a commercial game (SeaHeroQuest), is significant.

These numbers are evidence that it simply is much easier to find people who are interested in playing a citizen science game when interacting with the gaming world, than when interacting with the scientific world. And the more “gamers” a project is able to reach, the more players for the game.

This explains why projects such as SeaHeroQuest, a game-first game app with strong reach thanks to the high quality of its gameplay and the support from Deutsche Telekom, as massive telecommunication corporation, and Borderlands Science, a game-first mini-game embedded in a mass market game with millions of players, were able to reach about 40 times more players than Phylo in their first three years of existence.

## 11.2 Game-first design: A new paradigm for player attraction and retention

But being able to reach a large pool of potential players, while it definitely helps, is not sufficient. A game presented like a “real” mobile game like SeaHeroQuest creates expectations. By “real” here, we mean that the experience of the game is not distinguishable for the user from the experience they would get in a game purely designed for the enjoyment of the player. This is what we refer to as game-first design.

Similarly, a game element in a mass market game such as Borderlands 3, retailed at a retail price of $59.99 in the United States, comes with expectations.

While we have observed one big driver of player participation is excitement to help science, a failure to meet these expectations of game experience quality would likely affect the motivation of the player to keep playing.

It is difficult to compare player retention between different games, so we can hardly compare BLS to SeaHeroQuest on that front, but we can compare its retention against Phylo, a less gamified sequence alignment puzzle game (see main text discussion). It turns out that Borderlands Science displays much higher player retention, with players completing significantly more tasks than in Phylo.

We believe this is evidence that the ultra-gamification of Borderlands Science served its purpose, and that the mini-game fulfills the expectations created by its environment and does not break immersion for players.

In conclusion, we cannot simply copy and paste a scientific task inside a AAA game and expect success; it comes with a demanding gamification process, but correctly undergoing this process unlocks a reach ability that is not available to standard citizen science games.

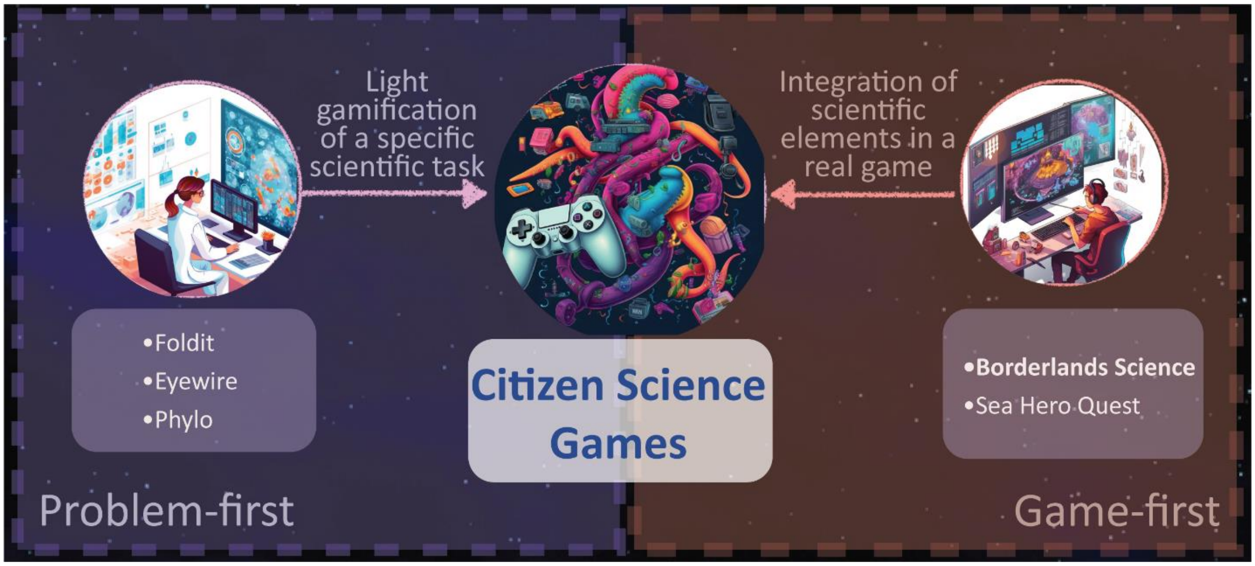


*Game-first design: we present the concept of game-first design for citizen science games. A game-first CSG is primarily a game with scientific elements added, as opposed to the typical problem-first CSG design in which a scientific task is gamified.*

## 11.3 On the resource intensiveness of Borderlands Science

It goes without saying that the methods Borderlands Science are compared to in this study are less resource intensive. The overhead cost to obtain Borderlands Science alignments is significant, between the talks with the game producer, the development of the game, the setup of the technical pipeline and the monitoring and processing of player solutions. However, the alignment presented here is not the only product of Borderlands Science. While the output alignment is the main result, the vast amounts of player data can be leveraged to train agents to mimic human strategies, such that these results can be useful in improving different alignments. Moreover, the large amount of data dedicated to this project allowed for the exploration of a paradigm change, by building a citizen science game as a collective quest to resolve one large problem through incremental tasks, rather than separate tasks as is typically done in CSG, It

also allowed a thorough investigation of the relationship between coverage and output alignment quality. Additionally, there is still a lot to unearth about how gamers play puzzle games, but there are few publicly available datasets of this type detailing the gameplay of millions of players. All the Borderlands Science player solutions are openly accessible and will be released as a Kaggle dataset for maximum accessibility.

But most importantly, demonstrating that a previously unseen level of gamification of the multiple alignment problem does not affect the relevance of player solutions to science, and demonstrating that embedding a citizen science game inside a fast paced mass market game can be done successfully, are two very significant achievements in the process of charting a course for the future of citizen science games..

## 11.4 On the impact of online citizen science games on players

With over 75% generation z, millennials and generation X engaging with mobile gaming, video games have reached the forefront of daily life for billions of people (Westcott and Arbanas 2021). The main enticing features of video games can be summarized in four components: the achievement aspect, interest in the rules and system, social connections, and the immersion aspect (Yee 2006). The achievement aspect can be interpreted as the desire to achieve flow, a concept from psychology describing a completely focused mental state achieved by a perfect level of challenge(Boyle, Connolly, and Hainey 2011).

This challenge aspect, which appears to be central in enjoyment of games, can be thought of as a problem solving task that can take various forms, from a brick puzzle on a mobile game to a maze in an adventure role-playing game. This rising yearning for problem solving challenges from a community of billions of gamers comes at a time when scientific data needing annotations are rising exponentially faster than the ability of scientists to annotate them, especially in fields such as genomics.

Citizen science games (CSGs) are initiatives that attempt to bridge the needs of the scientific community and those of the gaming community by producing games that provide an enjoyable challenge while providing useful scientific data. While the scientific impact of gamers on science through CSGs has been the focus of extensive study(Cooper et al. 2010; Lee et al. 2014; Waldispuhl and Blanchette 2014; Kim et al. 2014; Leifsson and Bjarkason 2015; Hyde et al. 2016; Mutalova et al. 2023), the impact of CSGs on their participants is potentially even more significant, albeit understudied.

Since CSGs constitute a relatively new field at 15 years old(Cooper et al. 2010), few studies have directly looked at the impact of CSGs on participants. However, that

impact has been extensively studied in more general games-with-a-purpose (GWAP), of which CSGs are a sub-category focused on science discovery, in online citizen science, and in gaming.

### 11.4.1 Impact of GWAP on participants

The study of the impact of playing a game with a purpose can be seen as a combination of the impact of gaming and the impact of the non-gaming component (the purpose). Healthy video game use has been shown to improve sub-second time perception, switching and attentional abilities(Nuyens et al. 2019) as well as helping reduce stress and anxiety. The social aspect was also seen as important by gamers(Yee 2006).

In parallel, numerous serious game studies have shown the potential of serious games as a learning tool for languages(Suh, Kim, and Kim 2010), sexual coercion awareness(Arnab et al. 2013), political science(Nishikawa and Jaeger 2011), nutrition(Peng 2009), history(Hess and Gunter 2013), and math(Ke 2008).

These successes highlight the potential of integrating serious elements leading to positive outcomes in gaming experiences.

### 11.4.2 Impact of citizen science projects on participants

The effects of participating in a citizen science project can be summarized in three categories: *learning*, *attitudes* and *other benefits*.

Many studies have identified clear impacts of participation in a citizen science project on participants' scientific knowledge, from increases in general scientific knowledge (such as understanding the nature of science) to project-specific information (Trumbull et al. 2000; Brossard, Lewenstein, and Bonney 2005; Bonney et al. 2009; Ceccaroni and Piera 2016; Cronje et al. 2011; Wiggins and Crowston 2011). Studies focusing on on school–based settings have also identified positive impacts on learning (Perelló et al. 2017).

Several studies have also identified clear improvements in attitude towards science from participation in citizen science projects. This improvement of attitude towards science usually materialized as participants finding science less intimidating and exhibiting more trust towards the scientific process and scientists after participation. (Toomey and Domroese 2013; Walker, Smigaj, and Tani 2021; Bruckermann et al. 2021; Brossard, Lewenstein, and Bonney 2005; Bonney et al. 2009; Haywood 2014)

Finally, other benefits include side-effects of participation in a project such as social interactions and health benefits (Vohland et al. 2021; Fitzpatrick et al. 2012), as-well as project-specific benefits such as improvement of physical health after participating in a project on physical activity (King et al. 2016).

As more and more citizen science projects transitioned to an online setting in the 2010s, questions were raised about whether these findings transferred to projects held entirely online. A review study by Aristeidou and Herodotou confirmed the increased positive attitude and better understanding of the nature of science were maintained for online project, but project-specific knowledge was not clearly improved (Aristeidou and Herodotou 2020). Other studies further investigated attitude changes and confirmed improvements for online projects (Santori et al. 2021), one study even identifying this change as the most significant one in an astronomy-focused citizen science project (C. A. Price and Lee 2013). The positive impact on attitude towards science was also confirmed in classroom projects involving purely online citizen science (Doyle et al. 2019).

As for other benefits, they were still present in online project, but different in nature. Instead of physical health benefits, improvements to digital literacy were observed (Aristeidou and Herodotou 2020). Additionally, the sense of community outcome identified in citizen science projects was still present online, but in a different form more similar to traditional gaming communities (Curtis n.d.)

### 11.4.3 What this means for citizen science games

We hypothesize that citizen science games have the potential to combine the positive impacts of games with regard to community and entertainment to the positive impacts of serious games centered around learning, and those of online citizen science centered such as attitude towards science. While there are few studies directly focusing on these effects, recent studies document circumstantial evidence of such a convergence.

In her book *Online Citizen Science and the Widening of Academia*, Vickie Curtis describes the strength of the community that built around Foldit, where 80% of participants report reading or posting on the Foldit forum, and many participants report having built new friendships through the game.

Moreover, several studies describe the motivations of CSG participants, and highlight motivation to help science as a central motivating factor (Curtis 2015; Díaz et al. 2020; Eveleigh et al. 2014; Iacovides et al. 2013; Jennett et al. 2016; Miller, Gandhi, and

Gander, n.d.). A consequent hypothesis is that this strong common aspect of motivation between gaming and non-gaming online citizen science projects indicates the positive consequence on attitude towards science identified in non-gaming online citizen science is likely to apply to CSGs, especially since informal science education in general has been shown to improve attitude towards science (Wulf et al. 2010; Sasson 2014; Nadkarni and Morris 2018)

Nevertheless, it should be mentioned that the transition to gaming focused projects likely comes with caveats such as expectations of players regarding visual design, gameplay enjoyability and storytelling. Indeed, Tang and Prestopnik (2022) have found that CSG participants may stop playing due to disappointing visual design or subpar gameplay (Tang and Prestopnik 2023). Miller et al also report that CSG players have reported losing interest in some games that provided insufficient gamification, and recommend tailoring tasks to gamer preferences through working with professional game designers (Miller, Gandhi, and Gander, n.d.)

### 11.4.4 Why are attitudes towards science so important?

A recurring aspect of studies on the impact of participating in a citizen science project is the improvement of attitude towards science. We insist on this aspect because it has been identified as central in determining vulnerability to scientific disinformation. Indeed, studies have found trust in science to be best predicted by positivistic attitudes towards science (Wintterlin et al. 2022; Rofieq and Fauzi 2022), and a quantitative study by Roberts, Reid and Norris found this relationship to be causal (Roberts et al. 2013).

Recently, a new generation of game-first citizen science games designed as mass-market games such as Sea Hero Quest (Hyde et al. 2016) or embedded in mass-market games such as Borderlands Science, in Borderlands 3 (Waldispühl et al. 2020) have brought a new paradigm in reach for citizen science projects by enlisting millions of participants within a year of their release.

Bringing online citizen science, which has been documented to improve attitudes towards science, to millions of people could be a game changer in the fight against disinformation.

## 11.5 Exchanges with the players

Through the Borderlands Science projects, we have had several opportunities to exchange with the players. Here are some links documenting these exchanges.

**Video trailer, played to players upon reaching the arcade machine in-game:**
https://youtu.be/L_mH6Ak_Ny0?si=_O0qYA__LYGHBMb1

**Blog posts (57,000 unique visitors in total), in chronological order:**

- Borderlands Science! https://dnapuzzles.org/2020/04/07/blog-post-1/
- Am I really helping by playing Borderlands Science? https://dnapuzzles.org/2020/04/13/am-i-really-helping-by-playing-borderlands-science/
- Borderlands Science: a peek at the data: https://dnapuzzles.org/2020/05/07/borderlands-science-update-a-peek-at-the-data/
- What we've been up to and what's coming for Borderlands Science: https://dnapuzzles.org/2020/06/07/what-weve-been-up-to-and-what-is-coming-for-borderlands-science/
- Decoding player submissions: identifying the best answers without knowing the right answer https://dnapuzzles.org/2020/07/08/decoding-player-submissions-identifying-the-best-answers-without-knowing-the-right-answer/
- From Phylo to Borderlands Science: https://dnapuzzles.org/2020/08/17/from-phylo-to-borderlands-science/
- Not all mutations are equal https://dnapuzzles.org/2020/09/01/not-all-mutations-are-equal/
- Towards a first takeaway from Borderlands Science - Phase 1 https://dnapuzzles.org/2020/12/15/towards-a-first-takeaway-from-borderlands-science-phase-1/
- Using puzzle solutions to aerate alignments https://dnapuzzles.org/2021/02/09/using-puzzle-solutions-to-aerate-alignments/
- Would you like to know more? https://dnapuzzles.org/2021/05/25/would-you-like-to-know-more/
- How to know these puzzles are right for you? https://dnapuzzles.org/2021/09/29/how-to-know-these-puzzles-are-right-for-you/
- Insight from how humans play Borderlands Science https://dnapuzzles.org/2021/12/09/insights-from-how-humans-play-borderlands-science/
- Borrowing successful strategies from participants https://dnapuzzles.org/2022/04/07/insights-from-how-humans-play-borderlands-science-2/
- When Borderlands Science meets Project Discovery: https://dnapuzzles.org/2023/11/13/when-borderlands-science-meets-project-discovery/

**Some Reddit AMAs (Ask Me Anything) presenting the first few blog posts:**

- https://www.reddit.com/r/borderlands3/comments/g0mh5n/faq_about_the_science_behind_borderlands_science/

- https://www.reddit.com/r/Borderlands/comments/lgawpa/borderlands_science_update_blog_a_peek_at_the/
- https://www.reddit.com/r/Borderlands/comments/gf9o2s/borderlands_science_update_a_peek_at_the_data/

**Conference talks discussing game design and feedback from players:**

- Challenges in designing scientific games, Gabriel Richard (Lead Designer), ISMB 2020, https://youtu.be/z6JsNUhc3xg?si=D1CBGsJ10zDNqCYH
- The making of Borderlands Science, Gabriel Richard (Lead Designer), GLBIO 2023, https://youtu.be/rP0P2s2biuQ?si=hg14cnyLWe8iaAM1

# 12. How can we evaluate alignments?

One of the central challenges of the Borderlands Science project was to evaluate the results. While building, delivering and analyzing solutions to millions of DNA puzzles is difficult, we were able to rely on solid assumptions based on the previous work to guide us.

However, when it comes to assessing the quality of the resulting alignment, things are suddenly a lot more complex due to the absence of an objective, universal method of determining the optimality of a multiple sequence alignment. This is especially true when the alignments we have to compare are extremely heterogeneous in their gap proportions, ranging from 175 to 800 columns

## 12.1 What is a good alignment?

Nevertheless, while we do not have access to a perfect evaluation method, we know what a high-quality alignment generally looks like.

A high-quality multiple sequence alignment of homologous genomic sequences from the gut microbiome will tend to:

- Have a high sum of pairs score
- Be similar to manually curated state-of-the-art alignments of that family
- Allow the inference of phylogenetic trees that resemble the state-of-the-art phylogeny of that family.
- Appropriately separate taxa associated with different illnesses, behavior and profiles in the host.
- Be compatible with the structural signature of the family, allowing the formation of the base pairs, RNA-protein and RNA-RNA interactions that are required for the RNA to fulfill its function

It is based on this set of assumptions about what defines a high-quality multiple sequence alignment that we selected the evidence presented in the results section of the main text.

## 12.2 A simple evaluation tool: sum of pairs

We computed and reported the sum of pairs scores excluding heavily gapped columns for the main result and benchmark alignments. Gaps are excluded because we are

comparing alignments of the same sequences with widths ranging from the 100s to the 900s.

| Method | PASTA | Post-proc PASTA | MUSCLE | MAFFT | Greedy | Borderlands Science |
|---|---|---|---|---|---|---|
| KC | 2193 | 1521 | 1772 | 1298 | 1246 | 1115 |
| Triplet (k) | 86.6 | 73.4 | 101.4 | 80.9 | 80.2 | 52.2 |
| Compound | 87.2 | 67.1 | 86.1 | 66.4 | 65.0 | 48.4 |
| Sum of pairs (B) | 2.09 | 2.18 | 2.05 | 2.11 | 2.16 | 2.17 |
| Width | 193 | 188 | 260 | 270 | 198 | 196 |

*Table 1: main results for sum of pairs and phylogeny, including sum of pairs scores and results for the BLS alignment*

## 12.3 Another simple evaluation: comparing to state-of-the-art alignments

In the absence of a perfect metric, a good approach to quickly evaluate whether a multiple sequence alignment is reasonable is to refer to previous work. There are several well-maintained alignments of 16S rRNAs with different focuses. The Greengenes database focuses on gut microbiome sequences, and maintains two alignments: one built with ssu-align (Eric Paul Nawrocki 2009), and one built with PyNAST (Caporaso et al. 2010). The Rfam database (Kalvari et al. 2021) focuses on high-quality structural alignments built with infeRNAl (Eric P. Nawrocki and Eddy 2013) and maintains a high quality seed alignment of the 16S rRNA.

Since the Rfam, Greengenes (Huber et al. 2006), and Borderlands Science alignments leverage different sequences, it is difficult to compare them directly, but one characteristic that can be assessed is the gap frequency. Indeed, in theory, the gap frequency in a multiple sequence alignment of homologous sequences should be somewhat representative of the insertion and deletion rate for that RNA region. In particular, since the V4 region of the 16S rRNA we are working with is strongly structured, the number of gaps in a perfect alignment should be relatively low. Thus, if Borderlands Science alignments are high-quality, we can expect its gap frequency will resemble those of state-of-the-art alignments.

To verify this, for each of the Greengenes-pynast, Greengenes-ssu-align, Rfam, Borderlands Science, PASTA, MUSCLE and MAFFT alignments, we sampled 50

sequences, removed fully gapped columns, and computed the mean number of gaps per sequence, repeating 1000 times. We obtain the following distributions:

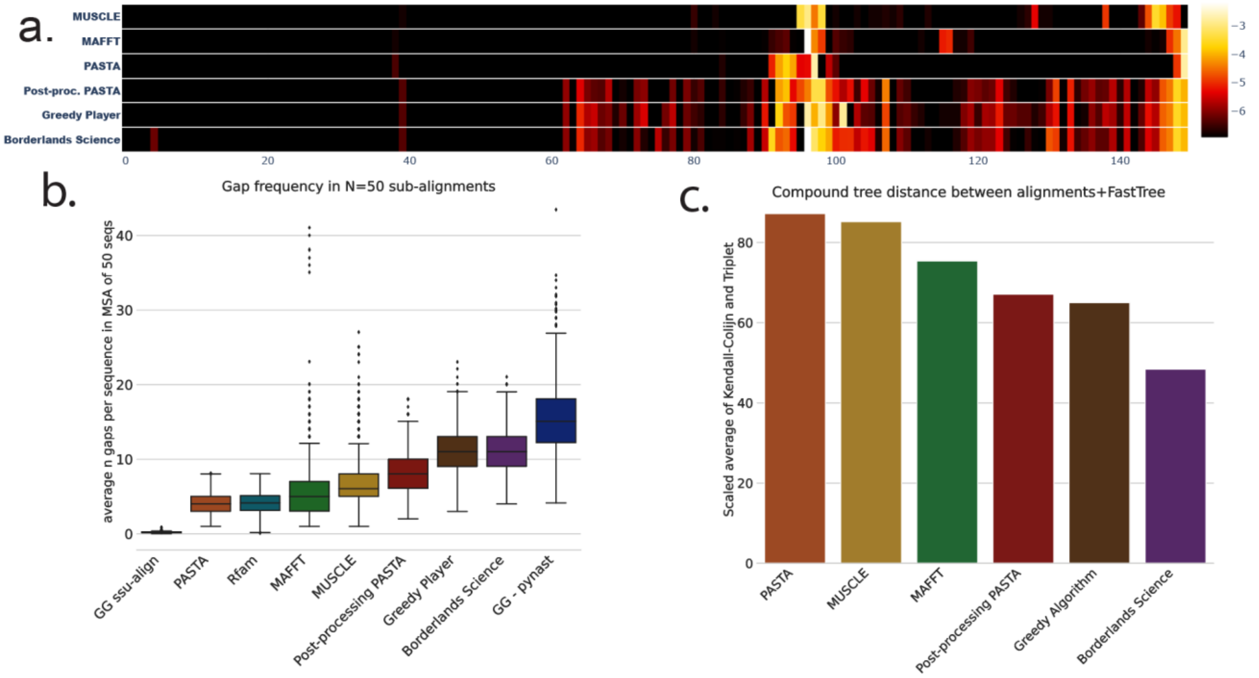


***Figure 3: Evaluation of the alignment.***

***a**. Here, the gap density by column is shown for six different alignment methods. The x axis corresponds to the alignment position, and the color to log gap frequency at this position. Highly gapped columns were excluded so all alignments could be of the same length.*

***b**. Gap frequencies observed in BLS and other methods, averaged from sampled sub-alignments of 50 sequences. The box plot shows an overview of the distribution. The three horizontal lines in the box, from top to bottom, show respectively the boundaries for the upper quartile, the middle quartile, and the lower quartile. The total height of the box represent the inter quartile range (IQR). The whiskers are located 1.5 times the IQR from the ends of the box. The dots outside the whiskers are the outliers. Note: one outlier point for the rightmost distribution, pynast, with a value of 124, is not shown on the plot but considered in the statistics shown.*

***c**. Compound distance to the reference Greengenes tree. That compound metric is obtained as a scaled average of the Kendall-Colijn and Triplet distance. More detail in Supp Mat sections 12(ab), 13(c)*

## 12.4 Evaluation via phylogeny

A central application of multiple sequence alignments in the context of the microbiome is for phylogeny-related applications. Indeed, a key factor for identifying associations with the gut microbiome is diversity, which requires an accurate phylogeny.

We detail how we evaluate the accuracy of Borderlands Science phylogeny in section 13.

## 12.5 Evaluation via effect sizes

The data presented in BLS puzzles comes from the American Gut Project, a collection of stool samples associated with extensive metadata (see section 1). A main objective of a deep analysis of this data should be the ability to accurately separate samples between donors with different metadata, such as young people and old people, people with diabetes from others, etc. Being able to accurately differentiate samples associated with behaviors and illnesses can enable the microbiome as a personalized medicine diagnosis down the road.

This association is tested by computing effect sizes of specific phylogenies on uniFrac distances, which measure how separated different sets of taxa are within a phylogeny. We report the specifics of the methods and the results of this computation in section 14.

## 12.6 Structure analysis

Finally, the last piece of evidence we include in the study is the structural aspect of the alignment. Indeed, an alignment of a strongly structured RNA region should be able to form the required base pairs and intermolecular interactions. We assess whether that is the case for our alignments by mapping them to the Rfam structural alignment, assessing how many nucleotides are excluded from mapping the full alignment to a 150-column alignment, and observing how well BLS alignments conform to structural models of the 16S rRNA. We detail this investigation in section 15.

# 13. Evaluating alignments via phylogeny

We use phylogeny as a proxy to evaluate the quality of our alignments compared to various benchmarks. We then look at similarity to a state of the art built with SEPP and Greengenes. To make sure we identify the main features of the relationships between alignments and trees, this analysis is performed on the 9,667 cluster representatives used in the puzzles, rather than the full dataset of nearly one million sequences. The full dataset is however used in the effect size computations in section 14.

For each alignment output generated by our pipeline, we use FastTree (Young, Faris, and Armogida 2021; M. N. Price, Dehal, and Arkin 2010) to build a phylogeny that we can then compare to state of the art phylogeny such as Greengenes (Huber et al. 2006).

We used FastTree with the *-fastest*, *-gamma* and *-spr 4* arguments for all our experiments.

## 13.1 Similarity to Greengenes Phylogeny

First, a reference tree is built by placing all the cluster representative sequences into a Greengenes 13.8 (Huber et al. 2006) phylogenetic tree using SEPP(Janssen et al. 2018), (Huber et al. 2006; S. Mirarab, Nguyen, and Warnow 2012) , then removing all other tips, and rerooting the tree for archaea. SEPP provides us with the most accurate estimation of what the phylogeny tree should look like given exterior knowledge, as rather than building a *de novo* phylogeny, we are placing our cluster representatives (9,667 tips) into an existing phylogeny of full length 16S rRNA sequences. We use the similarity to this tree as information about the quality of *de novo* phylogenies generated by our alignments: a *de novo* phylogeny estimated from an alignment that would be very similar to this reference tree would indicate that the alignment does a better job than others at organizing the sequences in a way that is more representative of what we know of their evolutionary relationship.

### 13.1.1 Kendall-Colijn Distance

Kendall-Colijn distance (Kendall and Colijn 2016) is a metric-based method for comparing trees which extracts distinct alternative evolutionary relationships embedded in data. It was designed specifically for situations where trees were too distinct for Robinson-Foulds distances (Robinson and Foulds 1981) to work as intended. This algorithm takes as input two rooted trees with identical sets of tip labels, and compares

the placement of the most recent common ancestor of each pair of tips. This placement is calculated using a combination of both distance from the root, as well as the number of edges between the most recent common ancestor and the root. Unfortunately, at this time previously published implementations of Kendall-Colijn in Python and R only support trees with up to 400 tips, therefore we sampled 400 random tips from both phylogenetic trees, removed all others, and calculated the Kendall-Colijn distance. This process was repeated 100 times to obtain an average, for each test.

### 13.1.2 Triplet Distance

The rooted triplet distance (Ranwez and Gascuel 2002) calculates the structural dissimilarity of two phylogenetic trees by counting the number of rooted phylogenetic trees with exactly three leaves that occur as embedded subtrees in one, but not both of them. Similar to Kendall-Colijn, the triplet distance is not designed to handle a tree with 9667 leaves. Due to its high time complexity, we randomly sample 100 tips from both phylogeny trees, remove the others, and use the resulting smaller tree for our triplet calculation. This is performed five times to obtain an average score.

### 13.1.2 Compound metric

Because the Kendall-Colijn and Triplet distances are on different scales, we designed a compound distance that combines the two in a simple-to-compute but fair average. Its formula is $(KC * 40 + Triplet)/2000$ , and it yields a value roughly between the 40s (very good) and the 90s (very bad). This formula was obtained by computing the ratio between distributions of Kendall-Colijn distance values and distributions of Triplet distance values.

| Method | PASTA | Post-proc PASTA | MUSCLE | MAFFT | Greedy | Borderlands Science |
|---|---|---|---|---|---|---|
| KC | 2193 | 1521 | 1772 | 1298 | 1246 | 1115 |
| Triplet (k) | 86.6 | 73.4 | 101.4 | 80.9 | 80.2 | 52.2 |
| Compound | 87.2 | 67.1 | 86.1 | 66.4 | 65.0 | 48.4 |
| Sum of pairs (B) | 2.09 | 2.18 | 2.05 | 2.11 | 2.16 | 2.17 |
| Width | 193 | 188 | 260 | 270 | 198 | 196 |

*Table 1: Main results for phylogeny, with kendall-colijn and triplet distance means*

## 13.3 Unused common metrics

In the process of identifying the best metrics to compare our methods to off-the-shelf software, we examined several state-of-the-art methods for comparing trees. In this section, we describe two methods that we ended up not including in the main text, despite them being common in literature.

### 13.4.1 Robinson-Foulds distance

The Robinson-Foulds distance (Robinson and Foulds 1981) is defined as the sum of splits between two trees. In other words, it is the sum of the data partitions that are in tree A but not in tree B, and the partitions that are in tree B but not in tree A. The Robinson-Foulds metric is useful for trees that are fundamentally similar near the root and diverge in their leaves. However, when comparing trees that are not very similar, it will tend to saturate quickly with relatively similar trees being quickly assigned near-maximal distances. This is what happens with the data used in this project; nearly all comparisons yield normalized RF distances over 0.9. We have thus decided not to include it in the results shown in the main text.

### 13.4.2 Mantel test

The Mantel test (Mantel 1967),(Diniz-Filho et al. 2013) is commonly used in comparative biology, namely to assess the correlation between the relative positioning of sets of tips in two trees. This test reports the correlation between two matrices of tip-to-tip distances for N tips present in two trees, and intends to assess how correlated the placement of the tips is between the two trees.

However, its use for this purpose has been contested (Harmon and Glor 2010), and we have personally observed over the duration of the process that it seemed very noisy; the correlations observed were reacting very strongly to small changes in the tested alignments. It is possible that the combination of alignments, FastTree and the Mantel test added an extra layer of bias. We elected not to report Mantel test results for these two reasons.

## 13.4 Are the phylogenetic trees different?

To complement our quantitative investigation of phylogenetic distances, we also explored the overall appearance of the full trees generated from our alignments. These are extremely large trees, with over 900,000 tips, so they cannot be shown with precision, but QIIME2's EMPRESS (Cantrell et al. 2021) tool is designed for displaying such large trees as well as it can be done For comparison, we show in this section the

EMPRESS output for Borderlands Science, MUSCLE, MAFFT, and the Greengenes+SEPP tree (see section 14) we use as a state-of-the-art benchmark.

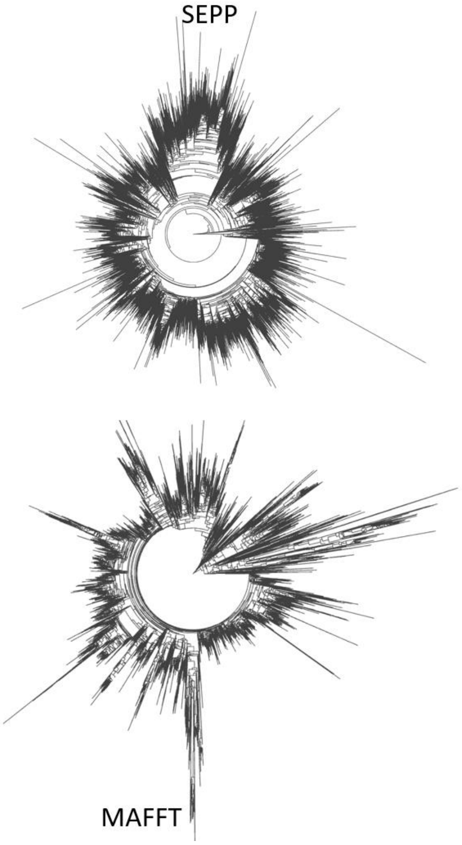


## 13.5 Phylogenetic distance as a primary optimization target

We distinguish three different levels of analysis of alignments: the alignment itself, the phylogenies inferred from the alignment, and the phenotypes associated with the phylogeny. We discussed the alignment itself in the previous section, and discuss the phenotype (effect sizes) in the next section.

Out of these three domains of evaluation, we chose to optimize on the phylogeny, because the alignments themselves had similar sum of pairs, and other evaluation metrics would have been too arbitrary, and because despite the American Gut Project data being the best dataset for this type of investigation, it remains relatively limited in size, with about 20 thousand data points, and quite noisy from its nature of coming from forms filled by human participants, often remotely. As a consequence, we believe that using the phylogenetic signal, generated on plentiful data, and then evaluating on phenotypic data, is the best way to avoid overfitting. We are confident in this decision because we know there is a well-established relationship between the quality of the tree and the effect sizes (Janssen et al. 2018), but by optimizing on tree metrics we

avoid overfitting to the effect sizes. Additionally, out of all the trees we have tested, the one that performs the best on tree metrics is also the one that performs the best on effect sizes.

## 13.6 Conclusion

In summary, we have shown here that the Borderlands Science alignment generates well-behaved phylogeny that are closer to the state of the art than trees built with alternative *de novo* methods for phylogeny inference, shown that phylogenies obtained from different alignment methods are significantly different, and explained why we focused on distance from Greengenes to drive our optimization process.

# 14. Evaluating alignments via effect sizes

## 14.1 Effect sizes computation

The second method we used to test the biological outcomes linked to Borderlands Science alignment was to apply this information to large scale datasets of observations about patients the genomes featured in the project were sequenced from. To achieve this, we propagated the alignment of cluster representatives to the sequences of their respective clusters with a standard profile alignment, computed phylogenies for these large alignments of about 950,000 sequences with FastTree(M. N. Price, Dehal, and Arkin 2010). To assess effect sizes,  a feature table representing the American Gut Project data was obtained from redbiom(McDonald et al. 2019), filtered for blooms, rarefied to 1000 sequences per sample, and unweighted *UniFrac* distances(Lozupone and Knight 2005),(Pilles 2018) were computed for each tree (e.g., the BLS tree, alignment benchmark trees, and the SEPP fragment insertion tree. Pairwise effect sizes were then calculated with Evident (Rahman et al., n.d.) on the distance matrix using Cohen's *d* distributions (Pilles 2018; Lewontin and Cohen 1969) for each tree.

Our benchmark for an upper bound performance of effect sizes for this dataset is obtained with a SEPP-placed tree, once again obtained from placing sequences into existing phylogenies from Greengenes. It should be noted that this tree is different from the other reference tree discussed in section 13. The tree in section 13 only contains 9,667 cluster representative tips, as the comparison methods fail on very large trees. Since effect sizes scale very well with data size, for the tests in this section we use a reference phylogenetic tree with all tips overlapping between the Borderlands Science dataset (945,000) and the American Gut project data we measured the effect sizes on.

We report effect sizes for all variables available in our dataset with at least two categories associated with minimum 50 samples, which adds up to 74 variables. We report absolute effect sizes, and delta effect sizes against SEPP.

## 14.2 Effect size results

The effect sizes for all variables are displayed on the next page. In the average of effect sizes over all variables, BLS slightly outperforms the rest of the field. The highest effect sizes for Borderlands Science are:

- Teeth brushing frequency
- Cdiff (prior *C. difficile* infection)
- Antibiotic history

- Vegetable frequency
- Country

The highest effect sizes for SEPP are
- Cdiff (prior *C. difficile* infection)
- Acne medication
- Multivitamin
- Alcohol consumption
- Country

For more information about the variables, see Section 1.

## 14.3 Effect size benchmark against SEPP

The variable-by-variable comparison between Borderlands Science and SEPP is shown two pages down. Borderlands Science outperforms SEPP on 49 of the 75 variables studied. The variables that are most improved are teeth brushing frequency, diabetes, types of plants, antibiotic history, alcohol frequency, vegetable frequency, irritable bowel disorder, kidney disease, bmi category, and small intestinal bacterial overgrowth. Out of these 10 variables, four are illnesses, and the others have previously been directly linked to human digestive health.

## 14.4 Significance

To back up our effect size results by checking if they could be the result of the distribution of categories for a given variable within our samples, we ran a Mann-Whitney U test between our results and results obtained from shuffled metadata. We repeated this process four times and presented results in the figure on page 66.

## 14.5 A note about effect sizes on MAFFT and MUSCLE

Computing effect sizes is computationally expensive: the whole process takes up to 3 days even on a powerful computer, and it occupies 50 to 100 GB of memory. For this reason, we were not able to compute effect sizes on dozens upon dozens of alignments. Thus, while we have a good understanding for how effect size results for BLS vary with changes to the alignment, and how SEPP effect sizes vary with the input (Janssen et al. 2018), our understanding of the impact of variations in parameters of MAFFT and MUSCLE on effect size results is imperfect. In this context, we erred on the side of caution by indicating the best results for effect sizes from benchmarks MAFFT and MUSCLE among the different versions of the alignment we have tested, in order to avoid under-representing the true performance of these software.

| variable | BLS | SEPP | offsets | greedy | pasta | ppp | mafft | muscle |
|---|---|---|---|---|---|---|---|---|
| acid_reflux | 0.146 | 0.078 | 0.143 | 0.136 | 0.135 | 0.142 | 0.126 | 0.044 |
| acne_medication | 0.198 | 0.344 | 0.184 | 0.218 | 0.196 | 0.212 | 0.252 | 0.381 |
| age_cat | 0.157 | 0.273 | 0.132 | 0.114 | 0.129 | 0.126 | 0.150 | 0.253 |
| alcohol_consumption | 0.296 | 0.320 | 0.299 | 0.314 | 0.297 | 0.301 | 0.295 | 0.314 |
| alcohol_frequency | 0.193 | 0.085 | 0.207 | 0.193 | 0.202 | 0.193 | 0.164 | 0.057 |
| antibiotic_history | 0.351 | 0.241 | 0.360 | 0.336 | 0.358 | 0.337 | 0.298 | 0.163 |
| artificial_sweeteners | 0.084 | 0.069 | 0.068 | 0.082 | 0.058 | 0.080 | 0.059 | 0.065 |
| asd | 0.077 | 0.146 | 0.086 | 0.112 | 0.074 | 0.064 | 0.062 | 0.123 |
| autoimmune | 0.077 | 0.079 | 0.074 | 0.130 | 0.061 | 0.071 | 0.046 | 0.117 |
| bmi_cat | 0.185 | 0.103 | 0.205 | 0.212 | 0.198 | 0.182 | 0.190 | 0.113 |
| bowel_movement_frequency | 0.330 | 0.275 | 0.360 | 0.370 | 0.345 | 0.336 | 0.363 | 0.221 |
| bowel_movement_quality | 0.175 | 0.207 | 0.167 | 0.174 | 0.164 | 0.173 | 0.180 | 0.181 |
| cancer | 0.155 | 0.148 | 0.137 | 0.136 | 0.122 | 0.117 | 0.137 | 0.143 |
| cardiovascular_disease | 0.222 | 0.167 | 0.207 | 0.183 | 0.187 | 0.214 | 0.227 | 0.150 |
| cdiff | 0.456 | 0.437 | 0.396 | 0.343 | 0.436 | 0.413 | 0.420 | 0.391 |
| chickenpox | 0.175 | 0.271 | 0.159 | 0.207 | 0.167 | 0.191 | 0.223 | 0.278 |
| cosmetics_frequency | 0.020 | 0.016 | 0.015 | 0.008 | 0.014 | 0.014 | 0.015 | 0.015 |
| country | 0.330 | 0.319 | 0.310 | 0.316 | 0.309 | 0.330 | 0.299 | 0.277 |
| country_residence | 0.284 | 0.296 | 0.259 | 0.251 | 0.258 | 0.278 | 0.263 | 0.299 |
| diabetes | 0.127 | 0.016 | 0.137 | 0.096 | 0.128 | 0.114 | 0.034 | 0.053 |
| diet_type | 0.090 | 0.063 | 0.102 | 0.091 | 0.099 | 0.100 | 0.099 | 0.078 |
| drinking_water_source | 0.167 | 0.154 | 0.168 | 0.164 | 0.159 | 0.168 | 0.148 | 0.113 |
| drinks_per_session | 0.265 | 0.236 | 0.256 | 0.252 | 0.238 | 0.253 | 0.243 | 0.229 |
| exercise_frequency | 0.141 | 0.090 | 0.155 | 0.154 | 0.144 | 0.145 | 0.140 | 0.104 |
| exercise_location | 0.049 | 0.013 | 0.046 | 0.044 | 0.042 | 0.048 | 0.021 | 0.027 |
| flossing_frequency | 0.092 | 0.108 | 0.094 | 0.087 | 0.094 | 0.089 | 0.096 | 0.084 |
| frozen_dessert_frequency | 0.199 | 0.151 | 0.199 | 0.180 | 0.208 | 0.196 | 0.219 | 0.164 |
| fruit_frequency | 0.198 | 0.157 | 0.194 | 0.182 | 0.183 | 0.192 | 0.187 | 0.130 |
| fungal_overgrowth | 0.203 | 0.145 | 0.199 | 0.197 | 0.196 | 0.185 | 0.177 | 0.098 |
| gluten | 0.123 | 0.094 | 0.140 | 0.153 | 0.137 | 0.124 | 0.130 | 0.091 |
| high_fat_red_meat_frequency | 0.134 | 0.134 | 0.150 | 0.106 | 0.145 | 0.131 | 0.147 | 0.079 |
| homecooked_meals_frequency | 0.251 | 0.207 | 0.266 | 0.283 | 0.276 | 0.276 | 0.250 | 0.194 |
| ibd | 0.205 | 0.117 | 0.209 | 0.130 | 0.179 | 0.183 | 0.235 | 0.118 |
| ibs | 0.085 | 0.050 | 0.083 | 0.066 | 0.075 | 0.064 | 0.057 | 0.021 |
| kidney_disease | 0.156 | 0.071 | 0.137 | 0.105 | 0.137 | 0.102 | 0.133 | 0.064 |
| lactose | 0.222 | 0.307 | 0.196 | 0.231 | 0.205 | 0.233 | 0.268 | 0.295 |
| liver_disease | 0.182 | 0.166 | 0.190 | 0.171 | 0.197 | 0.206 | 0.178 | 0.189 |
| lung_disease | 0.011 | 0.110 | 0.007 | 0.031 | 0.003 | 0.011 | 0.041 | 0.136 |
| meat_eggs_frequency | 0.117 | 0.105 | 0.141 | 0.131 | 0.145 | 0.131 | 0.120 | 0.090 |
| mental_illness | 0.064 | 0.100 | 0.066 | 0.068 | 0.055 | 0.058 | 0.057 | 0.106 |
| migraine | 0.091 | 0.088 | 0.068 | 0.078 | 0.081 | 0.093 | 0.101 | 0.094 |
| milk_cheese_frequency | 0.120 | 0.109 | 0.124 | 0.118 | 0.121 | 0.115 | 0.106 | 0.080 |
| milk_substitute_frequency | 0.048 | 0.045 | 0.039 | 0.041 | 0.038 | 0.037 | 0.037 | 0.054 |
| multivitamin | 0.229 | 0.328 | 0.204 | 0.234 | 0.207 | 0.231 | 0.269 | 0.310 |
| nail_biter | 0.222 | 0.297 | 0.193 | 0.223 | 0.203 | 0.221 | 0.257 | 0.287 |
| olive_oil | 0.184 | 0.134 | 0.193 | 0.188 | 0.187 | 0.189 | 0.150 | 0.118 |
| one_liter_of_water_a_day_frequency | 0.124 | 0.072 | 0.135 | 0.150 | 0.142 | 0.123 | 0.123 | 0.061 |
| pool_frequency | 0.227 | 0.163 | 0.243 | 0.232 | 0.221 | 0.241 | 0.222 | 0.184 |
| poultry_frequency | 0.093 | 0.053 | 0.096 | 0.092 | 0.081 | 0.085 | 0.088 | 0.069 |
| prepared_meals_frequency | 0.177 | 0.159 | 0.189 | 0.175 | 0.171 | 0.173 | 0.170 | 0.158 |
| probiotic_frequency | 0.110 | 0.118 | 0.107 | 0.105 | 0.098 | 0.099 | 0.103 | 0.090 |
| race | 0.079 | 0.141 | 0.076 | 0.098 | 0.076 | 0.079 | 0.084 | 0.128 |
| ready_to_eat_meals_frequency | 0.087 | 0.114 | 0.082 | 0.080 | 0.088 | 0.088 | 0.079 | 0.104 |
| red_meat_frequency | 0.086 | 0.101 | 0.100 | 0.091 | 0.086 | 0.089 | 0.098 | 0.063 |
| salted_snacks_frequency | 0.151 | 0.154 | 0.151 | 0.153 | 0.145 | 0.141 | 0.150 | 0.142 |
| seafood_frequency | 0.181 | 0.165 | 0.181 | 0.181 | 0.162 | 0.178 | 0.165 | 0.143 |
| seasonal_allergies | 0.191 | 0.282 | 0.159 | 0.197 | 0.174 | 0.200 | 0.226 | 0.279 |
| sex | 0.142 | 0.102 | 0.120 | 0.103 | 0.114 | 0.103 | 0.088 | 0.070 |
| sibo | 0.277 | 0.196 | 0.277 | 0.258 | 0.273 | 0.257 | 0.238 | 0.130 |
| skin_condition | 0.137 | 0.110 | 0.134 | 0.154 | 0.143 | 0.130 | 0.100 | 0.141 |
| sleep_duration | 0.099 | 0.056 | 0.108 | 0.108 | 0.102 | 0.091 | 0.091 | 0.058 |
| smoking_frequency | 0.109 | 0.107 | 0.112 | 0.106 | 0.098 | 0.112 | 0.088 | 0.103 |
| softener | 0.180 | 0.274 | 0.150 | 0.188 | 0.164 | 0.189 | 0.218 | 0.271 |
| sugar_sweetened_drink_frequency | 0.074 | 0.089 | 0.079 | 0.063 | 0.052 | 0.084 | 0.078 | 0.110 |
| sugary_sweets_frequency | 0.088 | 0.112 | 0.083 | 0.078 | 0.073 | 0.078 | 0.076 | 0.087 |
| teethbrushing_frequency | 0.495 | 0.292 | 0.525 | 0.592 | 0.489 | 0.489 | 0.620 | 0.333 |
| thyroid | 0.214 | 0.168 | 0.170 | 0.178 | 0.179 | 0.176 | 0.148 | 0.187 |
| tonsils_removed | 0.194 | 0.306 | 0.160 | 0.199 | 0.174 | 0.194 | 0.242 | 0.296 |
| types_of_plants | 0.166 | 0.055 | 0.179 | 0.173 | 0.170 | 0.177 | 0.139 | 0.036 |
| vegetable_frequency | 0.331 | 0.228 | 0.346 | 0.371 | 0.344 | 0.351 | 0.327 | 0.232 |
| vivid_dreams | 0.130 | 0.121 | 0.144 | 0.118 | 0.133 | 0.110 | 0.121 | 0.113 |
| weight_change | 0.133 | 0.118 | 0.133 | 0.123 | 0.124 | 0.128 | 0.095 | 0.089 |
| whole_eggs | 0.174 | 0.124 | 0.178 | 0.166 | 0.170 | 0.167 | 0.152 | 0.091 |
| whole_grain_frequency | 0.135 | 0.136 | 0.124 | 0.127 | 0.111 | 0.127 | 0.125 | 0.118 |
| mean | 0.168 | 0.157 | 0.166 | 0.166 | 0.161 | 0.164 | 0.164 | 0.147 |

*Pairwise UniFrac effect size results on Borderlands Science (**BLS**), **SEPP** + Greengenes, BLS with offset guide data (**offsets**), **greedy** player simulated game data, **PASTA**, PASTA with BLS post-processing **(ppp)**, **MAFFT**, and **MUSCLE**, on 74 metadata variables.*

*The colors are there for easier visualization, and correspond to a heat map of the values. White represents the average of the table, blue lower than average, and red higher than average.*

| variable | delta |
|---|---|
| teethbrushing_frequency | 0.203 |
| diabetes | 0.111 |
| types_of_plants | 0.111 |
| antibiotic_history | 0.110 |
| alcohol_frequency | 0.109 |
| vegetable_frequency | 0.103 |
| ibd | 0.088 |
| kidney_disease | 0.085 |
| bmi_cat | 0.082 |
| sibo | 0.081 |
| acid_reflux | 0.068 |
| pool_frequency | 0.064 |
| fungal_overgrowth | 0.058 |
| cardiovascular_disease | 0.055 |
| bowel_movement_frequency | 0.055 |
| one_liter_of_water_a_day_frequency | 0.051 |
| exercise_frequency | 0.051 |
| olive_oil | 0.050 |
| whole_eggs | 0.050 |
| frozen_dessert_frequency | 0.048 |
| thyroid | 0.046 |
| homecooked_meals_frequency | 0.043 |
| sleep_duration | 0.043 |
| poultry_frequency | 0.041 |
| fruit_frequency | 0.041 |
| sex | 0.040 |
| exercise_location | 0.036 |
| ibs | 0.035 |
| drinks_per_session | 0.029 |
| gluten | 0.028 |
| skin_condition | 0.027 |
| diet_type | 0.026 |
| cdiff | 0.020 |
| prepared_meals_frequency | 0.018 |
| liver_disease | 0.016 |
| seafood_frequency | 0.016 |
| weight_change | 0.015 |
| artificial_sweeteners | 0.015 |
| drinking_water_source | 0.013 |
| meat_eggs_frequency | 0.012 |
| country | 0.011 |
| milk_cheese_frequency | 0.010 |
| vivid_dreams | 0.009 |
| cancer | 0.007 |
| cosmetics_frequency | 0.004 |
| milk_substitute_frequency | 0.003 |
| migraine | 0.002 |
| smoking_frequency | 0.001 |
| high_fat_red_meat_frequency | -0.001 |
| whole_grain_frequency | -0.001 |
| autoimmune | -0.002 |
| salted_snacks_frequency | -0.004 |
| probiotic_frequency | -0.008 |
| country_residence | -0.013 |
| red_meat_frequency | -0.015 |
| sugar_sweetened_drink_frequency | -0.015 |
| flossing_frequency | -0.017 |
| sugary_sweets_frequency | -0.023 |
| alcohol_consumption | -0.024 |
| ready_to_eat_meals_frequency | -0.026 |
| bowel_movement_quality | -0.032 |
| mental_illness | -0.036 |
| race | -0.063 |
| asd | -0.069 |
| nail_biter | -0.075 |
| lactose | -0.085 |
| seasonal_allergies | -0.092 |
| softener | -0.094 |
| chickenpox | -0.096 |
| lung_disease | -0.098 |
| multivitamin | -0.099 |
| tonsils_removed | -0.112 |
| age_cat | -0.116 |
| acne_medication | -0.146 |
| mean | 0.055 |

*Per-variable difference between Pairwise UniFrac effect size results on Borderlands Science and SEPP+Greengenes, on 74 metadata variables.*

*The result shown represents BLS_effect_size - SEPP_effect_size, for each variable.*

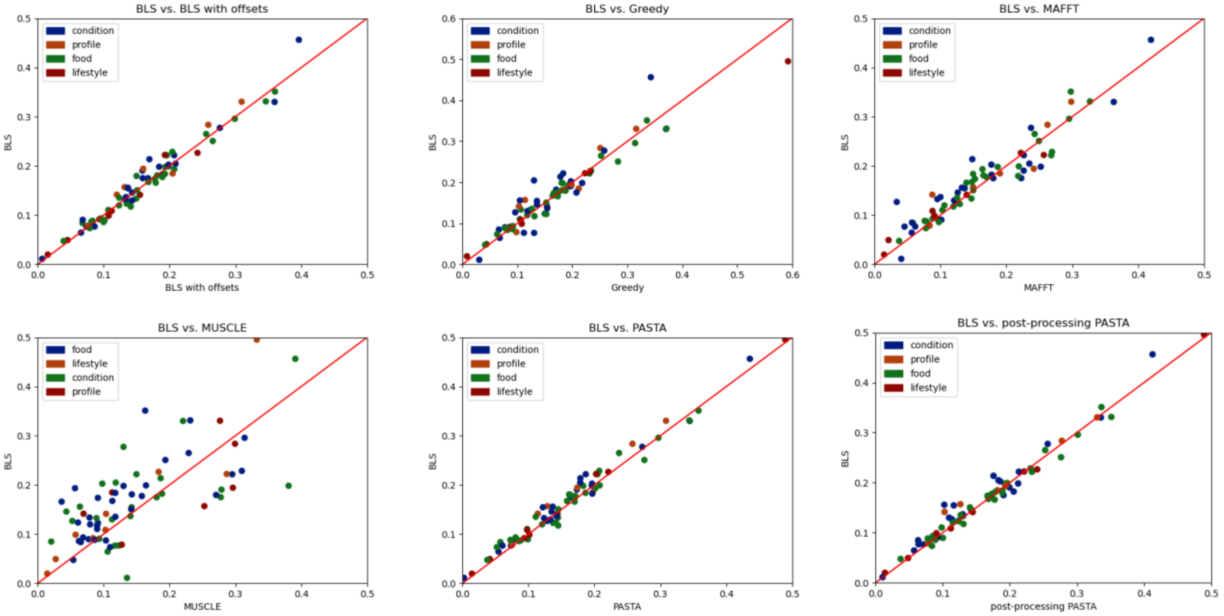


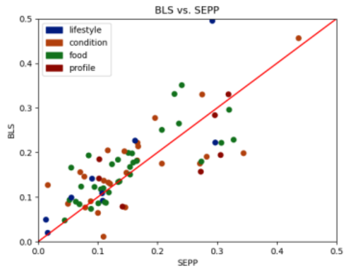


## Alignment-to-alignment comparison of effect size distribution

On this figure, we present comparisons of effect sizes distributions between BLS, SEPP, greedy player, PASTA, post-processing PASTA, MAFFT and MUSCLE. Each point represents a variable. The diagonal indicates that both methods are equal for that variable, and the distance towards one axis indicates how superior that method is for that variable. Colors correspond to the category of the effect size variable.

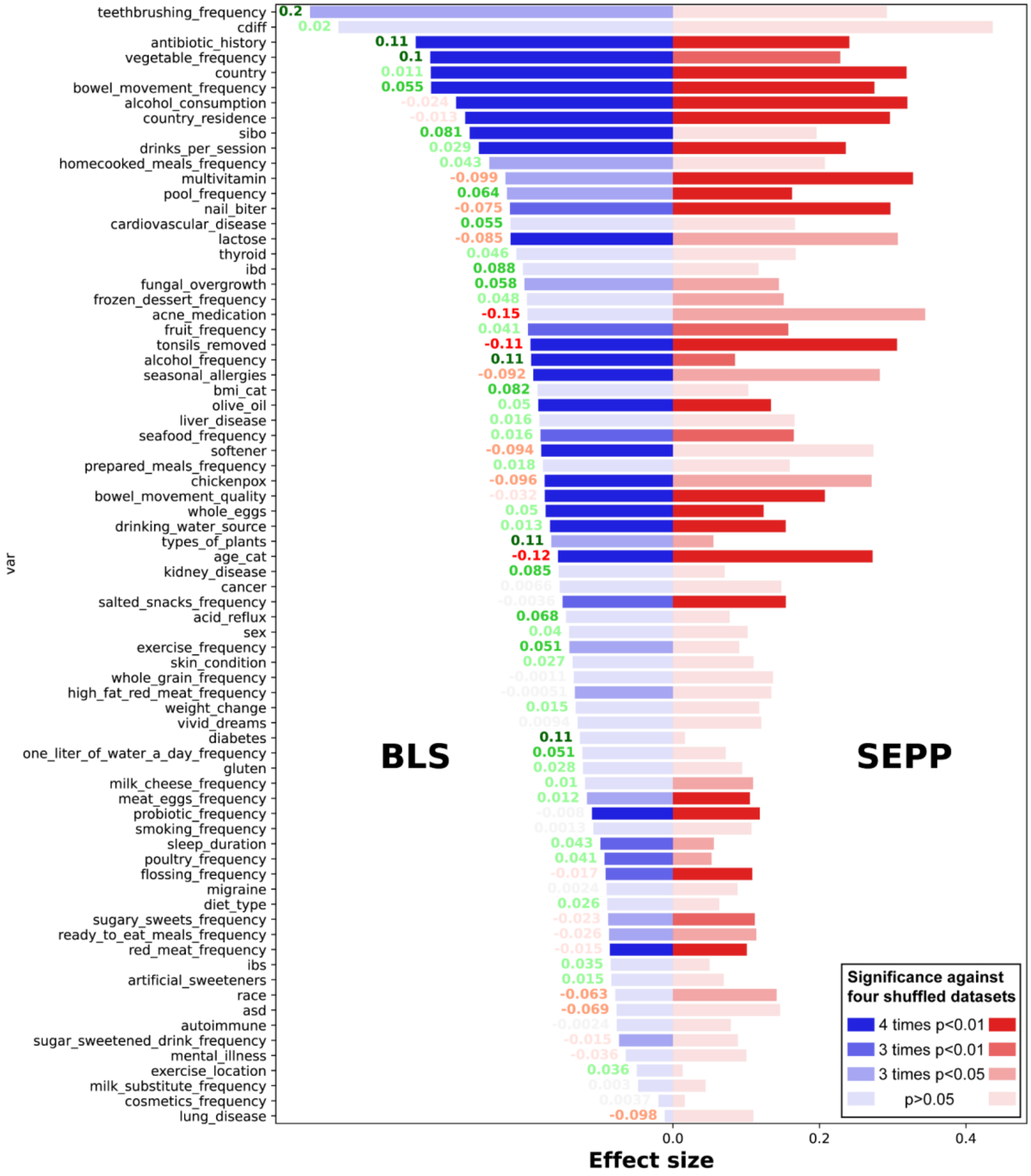


*Effect size results as presented in the main text, with significance (see 14.4)*

## 14.5 Conclusion

In this section, we have shown that the improvements identified in the alignments and phylogenetic trees from Borderlands Science over all benchmarks transfer to slight improvements in UniFrac effect sizes, especially compared to SEPP, despite the latter having been previously shown to outperform de novo phylogeny inference.

# 15. Evaluating alignments via structure

## 15.1 The 16S V4 region structure

The V4 region of the 16S rRNA is a hypervariable region that has been shown to provide resolution at the phylum level as accurately as the full 16S gene (Yang, Wang, and Qian 2016).

This region is well-conserved and tends to have few indels, as its structure is important for RNA-RNA and RNA-protein interactions, namely with the proteins S8 and S15.

This means we would expect a high quality alignment of genomic sequences from this region to have relatively few indels, but also many substitutions, due to the hypervariable nature of the region. (Van Der Pol et al. 2019)

The rest of this section is dedicated to the investigation of how well our multiple sequence alignments satisfy these expectations.

## 15.2 A ribosomal structure model

The ribosome has been at the forefront of RNA structure modeling for nearly 50 years, with the first secondary structure models determined in the late 1970s. With decades of ribosome structure data, we now have access to large sequence alignments of bacterial rRNAs, and precise sequence conservation annotations for each position in the structure.

In this section, we present a structural mapping of the Borderlands Science and benchmark alignments to the Rfam structure. This Rfam structure is itself mapped to the structural model on the Comparative RNA Web (Gutell Lab)(Woese et al. 1980; Gutell, Lee, and Cannone 2002), and we thus have expected nucleotide frequencies for each position of the structure: [https://crw2-comparative-rna-web.org/crw1_legacy/SAE/2A/nt_Frequency/index.htm](https://crw2-comparative-rna-web.org/crw1_legacy/SAE/2A/nt_Frequency/index.htm)

## 15.3 Mapping our alignments to structures

We mapped four alignments to the structural model: BLS, PASTA(Siavash Mirarab et al. 2015), MUSCLE(Edgar 2004) and MAFFT(Katoh 2002). Due to their narrowness, the BLS and PASTA alignments were easy to map, as only having to reduce from around 190 columns to 151 meant nearly every single conserved Rfam column was also present in BLS and PASTA. Thus, this mapping was done by hand and is easy to reproduce. However, the same cannot be said for the MAFFT and MUSCLE alignments, which have over 800 columns. To avoid an arbitrary manual mapping, we adopted a process similar to the one we use in the BLS realignment pipeline (section 10), to map MUSCLE and MAFFT alignments to a version of the PASTA alignment filtered to remove the approx 40 columns that were not mapped to the structure. We opted for this since, unlike Rfam, this PASTA alignment has the exact same nucleotides as those from MUSCLE and MAFFT, potentially making the mapping easier.

In order to map columns between two alignments, we created a profile from each alignment, and then used a Dynamic Programing algorithm based on a modification of the Needleman-Wunsch algorithm to align the two profiles together (see section 10). The DP method uses a SoP scoring scheme, and its difference with classic NW is that instead of two nucleotides at a time, it looks at two sets of nucleotides and calculates a match/mismatch score based on how common the nucleotides in the two sets are. While some columns might end up not being mapped to a column from the other alignment, it is possible to force all columns in the shorter alignment to be mapped to a column in the wider one.

As a result of this mapping, we were able to compute the amount of information lost in the mapping, i.e. the number of non-gap nucleotides that could not be mapped to the structure. As a reminder, these alignments have 9667 sequences of length 150.

| Method | PASTA | BLS | MUSCLE | MAFFT |
|---|---|---|---|---|
| Proportion unmapped bases | 0.001 | 0.003 | 0.149 | 0.100 |

*Proportion of non-gap nucleotides lost in the mapping from the BLS and benchmark alignments to the structural Rfam alignment of the 16S v4 region*

What we can see from this table is that the mapping for both BLS and PASTA preserves over 99.8% of the information. It is not obvious whether it is *better*, here, to have 600 unmapped nucleotides rather than 3200, as a nearly total absence of gaps is not necessarily a quality of a good alignment. One could argue that this table shows that the

Borderlands Science players may have identified about 3000 bases that were poorly aligned and moved them out of the columns that ended up being mapped to the structure. We investigate this hypothesis in section 15.4.

However, in the case of MUSCLE and MAFFT, we can clearly see that the alignments are simply not successful for this analysis. Removing 15% of the bases in the structural mapping assumes a 15% indel rate which is not consistent with the literature that suggests a very low indel rate.

## 15.4 A closer look: BLS and PASTA fidelity to model

To investigate whether the changes brought to the PASTA scaffold by Borderlands Science players can be considered error corrections rather than errors, we extracted the position-wise difference between the two alignments. In other words, for each column of the structural alignment (excluding columns that were not mapped to the structure), we identified each sequence that was different between BLS and PASTA.

Then, we assessed the frequency of the new nucleotide based on the structural model from the Comparative RNA Web(Woese et al. 1980; Gutell, Lee, and Cannone 2002). The objective was to assess whether the changes from PASTA to BLS bring the alignment closer to agreement with the structural model. We report the results on the following figure, on which green nucleotides mean that BLS changes are closer to the structural model, whereas red nucleotides mean PASTA is closer at this position. We observe that BLS is closer for most nodes, especially around regions associated with protein binding. (Yang, Wang, and Qian 2016; Serganov et al. 1996; Adilakshmi, Bellur, and Woodson 2008; Li, Ma, and Shapiro 2003; Nikulin et al. 2000)

## 15.5 Conclusion

In this section, we showed that the Borderlands Science and PASTA alignments were more compatible with the structural reality of the 16S rRNA V4 region than MAFFT and MUSCLE alignments, and that the changes between BLS and PASTA are generally in agreement with the CRW structural model.

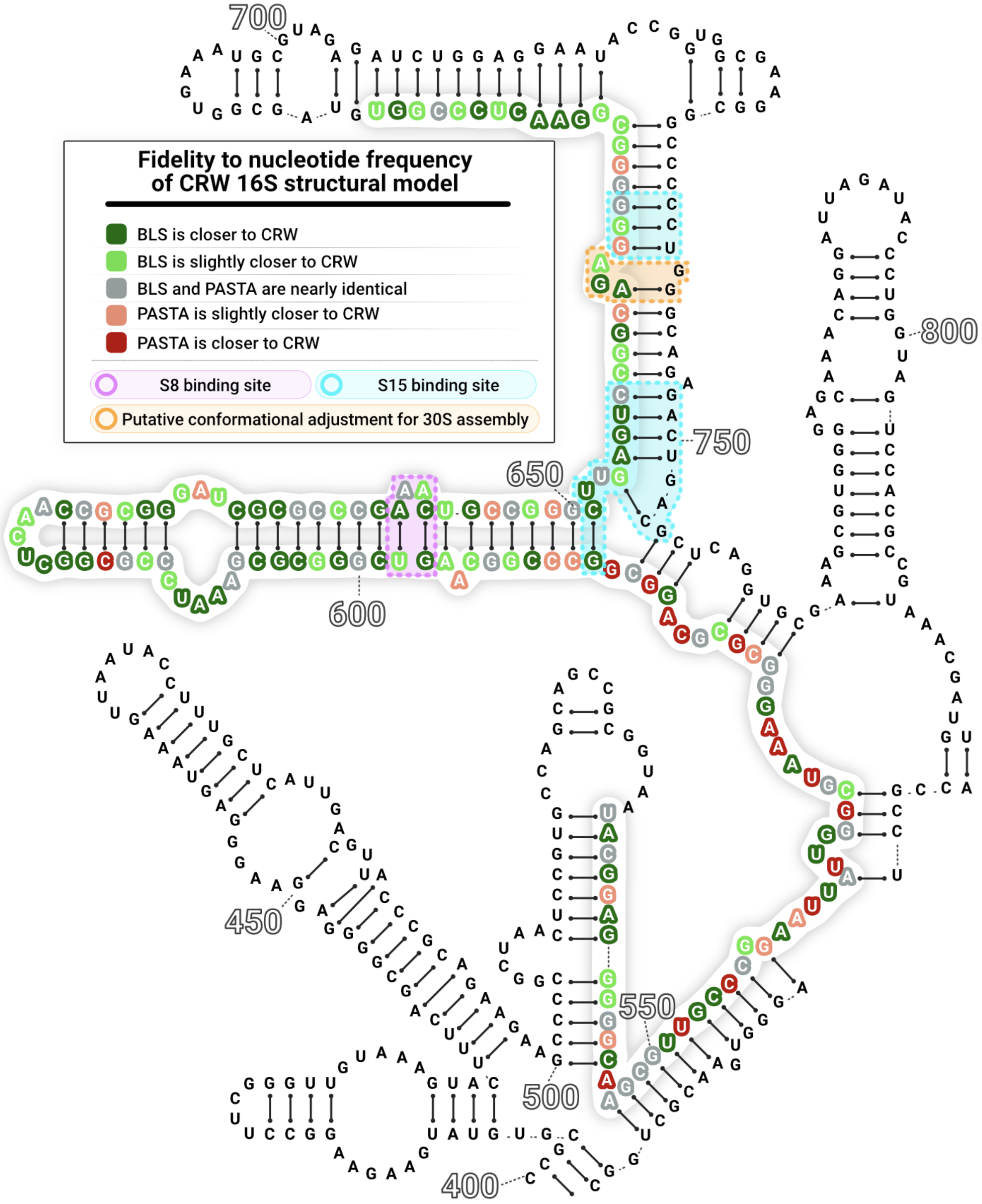


*Position-wise fidelity of the difference between BLS and PASTA. For each position, the nucleotides that differ between BLS and PASTA were compared to the nucleotide frequency of the CRW model. Positions identified to be significant functional sites are highlighted.*

# 16. Understanding effect size results

While UniFrac(Lozupone and Knight 2005) effect sizes provide valuable information about the usefulness of a phylogeny for the classification of human microbiome samples, its results are not always the most interpretable in the context of the phylogeny.

In this section, we propose some avenues of analysis to better understand how the differences identified in effect size results can be identified in the tree architecture.

## 16.1 Phylogenetic factorization of BLS and SEPP phylogenies

To better understand which clades were contributing the most to the effect size results, we leveraged PhyloFactor(Washburne et al. 2019), a software that identifies greatest contributing factors to metadata from a phylogenetic tree and a table with abundance data. Due to the limitations of Phylofactor, we collapsed the trees from 174k tips to about 24k tips (the maximal capacity of the software), and performed phylofactorization on the BLS and SEPP trees, identifying the top 4 factors for 10 variables with the highest performance difference between BLS and SEPP, with 5 (in green) in favor of BLS, and 5 (in red) in favor of SEPP. More detail on these results can be found in section 14.

We report the size and dominant phylogeny of each highlighted clade. Two cells of the same color within one table represent identical clades.

| Clade | BLS clade 1 | BLS clade 2 | BLS clade 3 | BLS clade 4 | SEPP clade 1 | SEPP clade 2 | SEPP clade 3 | SEPP clade 4 |
|---|---|---|---|---|---|---|---|---|
| Teethbrush freq | 796 prausnitzii | 67 Bifidobacter. | 266 Bacteroides | 70 Clostridiales | 279 Bifidobacter. | 1 prausnitzii | 277 Clostridiales | 416 Bacteroides |
| Types of plants | 70 Clostridiales | 220 prausnitzii | 88 Oscillospora | 147 Oscillospora | 277 Clostridiales | 1 prausnitzii | 745 Clostridiales | 416 Bacteroides |
| Diabetes | 796 prausnitzii | 70 Clostridiales | 266 Bacteroides | 33 Lachnospira | 1 prausnitzii | 155 prauznitzii | 277 Clostridiales | 745 Clostridiales |
| Alcohol freq | 35 Human Gut metagenome | 220 prausnitzii | 147 Oscillospora | 195 Parabacteroides | 102 massiliensis | 1 prausnitzii | 745 Clostridiales | 416 Bacteroides |
| Antibiotics | 70 Clostridiales | 220 prausnitzii | 88 Oscillospora | 97 Erysipelotrich. | 745 Clostridiales | 277 Clostridiales | 1 prausnitzii | 250 Ruminococc. |
| Age category | 67 Bifidobacter. | 102 muciniphila | 147 Oscillospora | 80 Bacteroides | 279 Bifidobacter. | 416 Bacteroides | 252 Oscillospora | 184 muciniphila |
| Acne medic. | 233 Odoribacter | 97 Erysipelotrich. | 266 Bacteroides | 168 P. copri | 328 Odoribacter | 505 Butyricimon. | 925 Erysipelotric. | 483 Clostridiales |
| Multivitamin | 233 Odoribacter | 97 Erysipelotrich | 220 prausnitzii | 70 Clostridiales | 328 Odoribacter | 505 Butyricimon. | 426 Erysipelotric. | 1 Prausnitzii |
| Tonsils rem. | 233 Odoribacter | 97 Erysipelotrich | 67 Bifidobacter | 168 P. copri | 328 Odoribacter | 505 Butryricimon | 925 Erysipelotric. | 279 Bifidobacter |
| Lung disease | 220 prauznitzii | 266 Bacteroides | 164 A putrenidis | 70 Clostridiales | 745 Clostridiales | 1 prausnitzii | 416 Bacteroides | 119 Ruminococc |

We observe that the dominant clades seem to be quite similar for the red variables (the ones SEPP dominates on), whereas they are more distinct for the green variables.

## 16.2 Comparison of effect size profiles

We presented in section 14 detailed effect size results over 74 variables for 8 alignments. The heatmaps help identify some similarity between the overall effect size profiles, but in order to clarify this similarity, we performed Principal Component Analysis on the 74-dimension effect size contribution and summarized it in three components to visually display the relative similarity between effect size profiles.

On the following plot, two alignments are displayed in proximity when they tend to respectively perform well and poorly on the same variables.

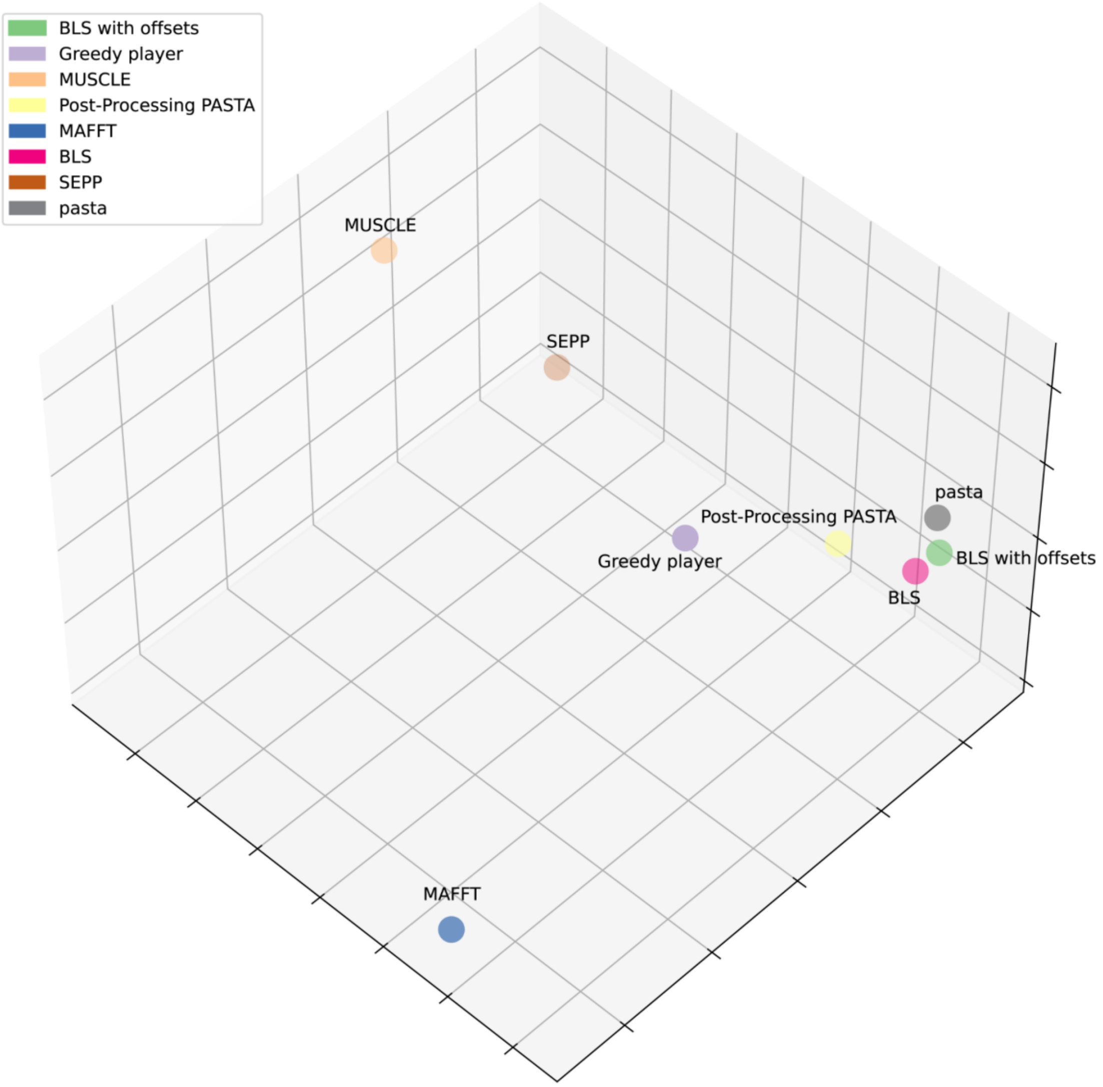


*3D visualization of the Principal Component Analysis of the effect size performance profile for BLS alignments and benchmarks.*

We observe here that BLS, BLS with offsets, and PASTA are close-by. This suggests that the improvements made to PASTA by BLS players improved the performance of

the alignment without changing it massively, whereas the changes brought by the simulated greedy player were significant.

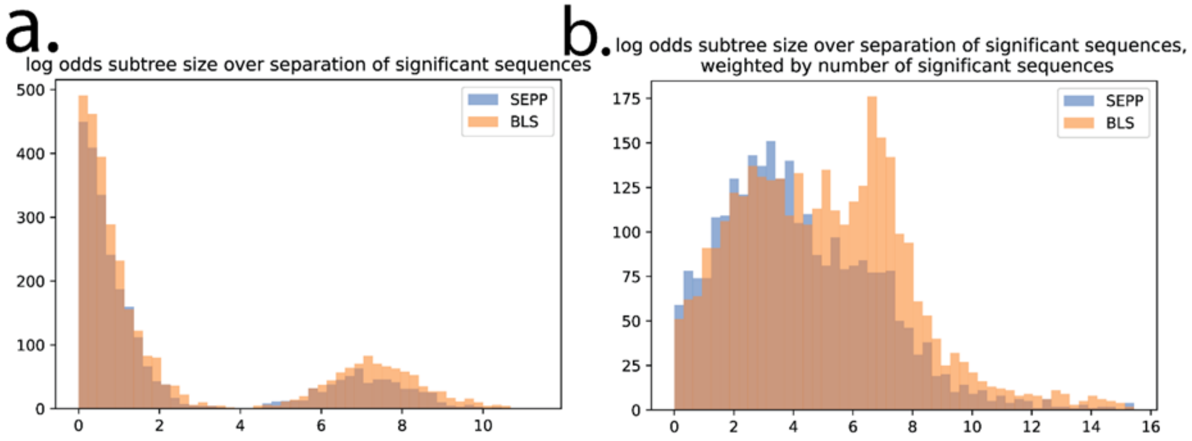


*Distribution of separation of tips in the BL and SEPP tree. In a, we show the distribution of log odds of the ratio of the splitting of tips between the two children, over the splitting of sequences overrepresented in samples showing daily teeth brushing. In b, we show the same distributions, but multiplied by the number of teeth-brushing relevant sequences affected, showing that while the two distributions are highly similar, the most surprising tips in BLS affect more sequences than their homologs in SEPP.*

## 16.3 Reflection of effect sizes in tree architecture (or vice-versa)

The results presented in section 14 show an effect size difference of 0.2 between Borderlands Science and SEPP on teeth brushing frequency. A difference of this magnitude is surprising, as it more than doubles the values of the 73 other variables examined.

To better understand how this difference in effect sizes reflects in the phylogeny, we highlighted ~9000 sequences that were vastly overrepresented (>10 standard deviations above per-sample mean) in samples from participants who brush their teeth daily.

To understand how the sequences relevant to teeth brushing were distributed in both trees, we computed, for each internal node, the log odds of the ratio of the splitting of tips between the two children, over the splitting of the sequences overrepresented with daily teeth brushing. The distribution we obtained was similar between BLS and SEPP (See left panel of figure). However, multiplying the ratio by the number of teeth brushing-associated sequences at each node shows different distributions (see right panel of figure).

This appears to indicate the BLS phylogeny has higher effect sizes because the internal nodes where the separation of teeth brushing-relevant sequence is most different from the separation of tips in general affect a higher number of such sequences.

In other words, the UniFrac effect size result that shows Borderlands Science distinguishes better between taxa related to teeth brushing is backed up by the architecture of the tree.

# 17. Other Borderlands Science publications

Borderlands Science is a multi-year project involving multiple labs and dozens of authors. This amount of work cannot possibly be contained to a single paper. In this section, we provide an overview of the work done in Borderlands Science that is not described in this study.

## 17.1 AI and reinforcement learning

A core part of the Borderlands Science project is to use the game as an opportunity to build an open-source database of game solutions for the purpose of training AI agents. Indeed, it goes without saying that a game of this scale cannot be set up every time a new dataset needs to be aligned.

We have been working with behaviour cloning to characterize the strategies employed by humans, their heterogeneity and their reproducibility, with positive results so far. We have published some of these results at CHI 2023 (Mutalova et al. 2023) and Collective Intelligence 2023 (Mutalova et al. 2023). The first paper focuses on showing that player strategies can be learned by behaviour cloning methods, and the second one on showing that these methods constitute improvements over pure AI. We are actively working on follow-ups to these publications.

We are still actively working on identifying the potential of reproducing human strategies for the sequence alignment task, and on finding the best AI method to harness the wisdom of the crowd.

## 17.2 Human-computer interactions and games

Another major focus of the project is the human-computer interaction (HCI) aspect of the game. Indeed, it is one thing to formulate the hypothesis that humans are good at solving this type of problem, and it's another thing to show that human solutions improve multiple sequence alignments compared to off-the-shelf computational methods, but this does not tell us why, or how, this is the case.

This is why another focus of our work has been to better understand how players play the game. This includes looking at the differences in performance and strategies between beginners and expert players, but also analyzing the contribution of different aspects of the game and reflecting on how the game could be improved in its playability and in its scientific contribution.

## 17.3 Future work

Along with the AI and HCI aspects of the project, we will keep working on processing more data, diving deeper on how to leverage this heterogeneous data to extract biologically-relevant information, and on new citizen science game projects. You can follow our papers, preprints and games at https://games.cs.mcgill.ca/bls/

# 18. References


Adilakshmi, Tadepalli, Deepti L. Bellur, and Sarah A. Woodson. 2008. “Concurrent Nucleation of 16S Folding and Induced Fit in 30S Ribosome Assembly.” *Nature* 455 (7217): 1268–72.

Aristeidou, Maria, and Christothea Herodotou. 2020. “Online Citizen Science: A Systematic Review of Effects on Learning and Scientific Literacy.” *Citizen Science: Theory and Practice* 5 (1): 1–12.

Arnab, Sylvester, Katherine Brown, Samantha Clarke, Ian Dunwell, Theodore Lim, Neil Suttie, Sandy Louchart, Maurice Hendrix, and Sara de Freitas. 2013. “The Development Approach of a Pedagogically-Driven Serious Game to Support Relationship and Sex Education (RSE) within a Classroom Setting.” *Computers & Education* 69 (November): 15–30.

Bonney, Rick, Caren B. Cooper, Janis Dickinson, Steve Kelling, Tina Phillips, Kenneth V. Rosenberg, and Jennifer Shirk. 2009. “Citizen Science: A Developing Tool for Expanding Science Knowledge and Scientific Literacy.” *Bioscience* 59 (11): 977–84.

Boyle, Elizabeth, Thomas M. Connolly, and Thomas Hainey. 2011. “The Role of Psychology in Understanding the Impact of Computer Games.” *Entertainment Computing* 2 (2): 69–74.

Brossard, Dominique, Bruce Lewenstein, and Rick Bonney. 2005. “Scientific Knowledge and Attitude Change: The Impact of a Citizen Science Project.” *International Journal of Science Education* 27 (9): 1099–1121.

Bruckermann, Till, Hannah Greving, Anke Schumann, Milena Stillfried, Konstantin Börner, Sophia E. Kimmig, Robert Hagen, Miriam Brandt, and Ute Harms. 2021. “To Know about Science Is to Love It? Unraveling Cause–effect Relationships between Knowledge and Attitudes toward Science in Citizen Science on Urban Wildlife Ecology.” *Journal of Research in Science Teaching* 58 (8): 1179–1202.

Cantrell, Kalen, Marcus W. Fedarko, Gibraan Rahman, Daniel McDonald, Yimeng Yang, Thant Zaw, Antonio Gonzalez, et al. 2021. “EMPress Enables Tree-Guided, Interactive, and Exploratory Analyses of Multi-Omic Data Sets.” *mSystems* 6 (2). https://doi.org/10.1128/mSystems.01216-20.

Caporaso, J. Gregory, Kyle Bittinger, Frederic D. Bushman, Todd Z. DeSantis, Gary L. Andersen, and Rob Knight. 2010. “PyNAST: A Flexible Tool for Aligning Sequences to a Template Alignment.” *Bioinformatics* 26 (2): 266–67.

Ceccaroni, Luigi, and Jaume Piera. 2016. *Analyzing the Role of Citizen Science in Modern Research*. IGI Global.

Cooper, Seth, Firas Khatib, Adrien Treuille, Janos Barbero, Jeehyung Lee, Michael Beenen, Andrew Leaver-Fay, David Baker, Zoran Popović, and Foldit Players. 2010. “Predicting Protein Structures with a Multiplayer Online Game.” *Nature* 466 (7307): 756–60.

Cronje, Ruth, Spencer Rohlinger, Alycia Crall, and Greg Newman. 2011. “Does Participation in Citizen Science Improve Scientific Literacy? A Study to Compare Assessment Methods.” *Applied Environmental Education and Communication* 10 (3): 135–45.

Curtis, Vickie. 2015. “Motivation to Participate in an Online Citizen Science Game: A

Study of Foldit." *Science Communication* 37 (6): 723–46.
———. n.d. *Online Citizen Science and the Widening of Academia*. Springer International Publishing. Accessed June 11, 2023.
Díaz, Carlos, Marisa Ponti, Pinja Haikka, Rajiv Basaiawmoit, and Jacob Sherson. 2020. "More than Data Gatherers: Exploring Player Experience in a Citizen Science Game." *Quality and User Experience* 5 (1). https://doi.org/10.1007/s41233-019-0030-8.
Diniz-Filho, José Alexandre F., Thannya N. Soares, Jacqueline S. Lima, Ricardo Dobrovolski, Victor Lemes Landeiro, Mariana Pires de Campos Telles, Thiago F. Rangel, and Luis Mauricio Bini. 2013. "Mantel Test in Population Genetics." *Genetics and Molecular Biology* 36 (4): 475–85.
Doyle, Cathal, Rodreck David, Yevgeniya Li, Markus Luczak-Roesch, Dayle Anderson, and Cameron M. Pierson. 2019. "Using the Web for Science in the Classroom: Online Citizen Science Participation in Teaching and Learning." In *Proceedings of the 10th ACM Conference on Web Science*, 71–80. WebSci '19. New York, NY, USA: Association for Computing Machinery.
Edgar, Robert C. 2004. "MUSCLE: Multiple Sequence Alignment with High Accuracy and High Throughput." *Nucleic Acids Research* 32 (5): 1792–97.
Eveleigh, Alexandra, Charlene Jennett, Ann Blandford, Philip Brohan, and Anna L. Cox. 2014. "Designing for Dabblers and Deterring Drop-Outs in Citizen Science." In *Proceedings of the SIGCHI Conference on Human Factors in Computing Systems*, 2985–94. CHI '14. New York, NY, USA: Association for Computing Machinery.
Fitzpatrick, John W., Richard Louv, Richard E. Bonney Jr, and Janis L. Dickinson. 2012. *Citizen Science: Public Participation in Environmental Research*. Cornell University Press.
Fu, Limin, Beifang Niu, Zhengwei Zhu, Sitao Wu, and Weizhong Li. 2012. "CD-HIT: Accelerated for Clustering the next-Generation Sequencing Data." *Bioinformatics* 28 (23): 3150–52.
Gilbert, Jack A., Janet K. Jansson, and Rob Knight. 2014. "The Earth Microbiome Project: Successes and Aspirations." *BMC Biology* 12 (August): 69.
Gutell, Robin R., Jung C. Lee, and Jamie J. Cannone. 2002. "The Accuracy of Ribosomal RNA Comparative Structure Models." *Current Opinion in Structural Biology* 12 (3): 301–10.
Hall, Michael, and Robert G. Beiko. 2018. "16S rRNA Gene Analysis with QIIME2." *Methods in Molecular Biology* 1849: 113–29.
Harmon, Luke J., and Richard E. Glor. 2010. "Poor Statistical Performance of the Mantel Test in Phylogenetic Comparative Analyses." *Evolution; International Journal of Organic Evolution* 64 (7): 2173–78.
Haywood, Benjamin K. 2014. "A 'sense of Place' in Public Participation in Scientific Research." *Science Education* 98 (1): 64–83.
Hess, Taryn, and Glenda Gunter. 2013. "Serious Game-Based and Nongame-Based Online Courses: Learning Experiences and Outcomes." *British Journal of Educational Technology: Journal of the Council for Educational Technology* 44 (3): 372–85.
Holzinger, Andreas. 2016. "Interactive Machine Learning for Health Informatics: When Do We Need the Human-in-the-Loop?" *Brain Informatics* 3 (2): 119–31.

Huber, T., K. Keller, G. L. Andersen, P. Hugenholtz, N. Larsen, M. Rojas, T. Z. DeSantis, E. L. Brodie, and D. Dalevi. 2006. *Greengenes: Chimera-Checked 16S RRNA Gene Database and Workbenchcompatible in ARB*.

Hyde, Matthew, Maxwell Scott-Slade, Hugo Scott-Slade, Michael Hornberger, Hugo Spiers, Ruth Dalton, Christoph Hoelscher, Jan Wiener, and Veronique Bohbot. 2016. “Sea Hero Quest: The World’s First Mobile Game Where Anyone Can Help Scientists Fight Dementia.” https://eprints.lancs.ac.uk/id/eprint/138622/.

Iacovides, Ioanna, Charlene Jennett, Cassandra Cornish-Trestrail, and Anna L. Cox. 2013. “Do Games Attract or Sustain Engagement in Citizen Science? A Study of Volunteer Motivations.” In *CHI ’13 Extended Abstracts on Human Factors in Computing Systems*, 1101–6. CHI EA ’13. New York, NY, USA: Association for Computing Machinery.

Janssen, Stefan, Daniel McDonald, Antonio Gonzalez, Jose A. Navas-Molina, Lingjing Jiang, Zhenjiang Zech Xu, Kevin Winker, et al. 2018. “Phylogenetic Placement of Exact Amplicon Sequences Improves Associations with Clinical Information.” *mSystems* 3 (3). https://doi.org/10.1128/mSystems.00021-18.

Jennett, Charlene, Laure Kloetzer, Daniel Schneider, Ioanna Iacovides, Anna Cox, Margaret Gold, Brian Fuchs, et al. 2016. “Motivations, Learning and Creativity in Online Citizen Science.” *Journal of Science Communication* 15 (3). http://oro.open.ac.uk/47008/.

Kalvari, Ioanna, Eric P. Nawrocki, Nancy Ontiveros-Palacios, Joanna Argasinska, Kevin Lamkiewicz, Manja Marz, Sam Griffiths-Jones, et al. 2021. “Rfam 14: Expanded Coverage of Metagenomic, Viral and microRNA Families.” *Nucleic Acids Research* 49 (D1): D192–200.

Katoh, K. 2002. “MAFFT: A Novel Method for Rapid Multiple Sequence Alignment Based on Fast Fourier Transform.” *Nucleic Acids Research*. https://doi.org/10.1093/nar/gkf436.

Kawrykow, Alexander, Gary Roumanis, Alfred Kam, Daniel Kwak, Clarence Leung, Chu Wu, Eleyine Zarour, et al. 2012. “Phylo: A Citizen Science Approach for Improving Multiple Sequence Alignment.” *PloS One* 7 (3): e31362.

Ke, Fengfeng. 2008. “A Case Study of Computer Gaming for Math: Engaged Learning from Gameplay?” *Computers & Education* 51 (4): 1609–20.

Kendall, Michelle, and Caroline Colijn. 2016. “Mapping Phylogenetic Trees to Reveal Distinct Patterns of Evolution.” *Molecular Biology and Evolution* 33 (10): 2735–43.

Kim, Jinseop S., Matthew J. Greene, Aleksandar Zlateski, Kisuk Lee, Mark Richardson, Srinivas C. Turaga, Michael Purcaro, et al. 2014. “Space-Time Wiring Specificity Supports Direction Selectivity in the Retina.” *Nature* 509 (7500): 331–36.

King, Abby C., Sandra J. Winter, Jylana L. Sheats, Lisa G. Rosas, Matthew P. Buman, Deborah Salvo, Nicole M. Rodriguez, et al. 2016. “Leveraging Citizen Science and Information Technology for Population Physical Activity Promotion.” *Translational Journal of the American College of Sports Medicine* 1 (4): 30–44.

Lee, Jeehyung, Wipapat Kladwang, Minjae Lee, Daniel Cantu, Martin Azizyan, Hanjoo Kim, Alex Limpaecher, et al. 2014. “RNA Design Rules from a Massive Open Laboratory.” *Proceedings of the National Academy of Sciences* 111 (6): 2122–27.

Leifsson, Hjalti, and Jóhann Örn Bjarkason. 2015. “Project Discovery-Advancing Scientific Research by Implementing Citizen Science in EVE Online.”

Lewontin, R. C., and D. Cohen. 1969. “On Population Growth in a Randomly Varying Environment.” *Proceedings of the National Academy of Sciences of the United States of America* 62 (4): 1056–60.
Li, Wen, Buyong Ma, and Bruce A. Shapiro. 2003. “Binding Interactions between the Core Central Domain of 16S rRNA and the Ribosomal Protein S15 Determined by Molecular Dynamics Simulations.” *Nucleic Acids Research* 31 (2): 629–38.
Lozupone, Catherine, and Rob Knight. 2005. “UniFrac: A New Phylogenetic Method for Comparing Microbial Communities.” *Applied and Environmental Microbiology* 71 (12): 8228–35.
Mantel, N. 1967. “The Detection of Disease Clustering and a Generalized Regression Approach.” *Cancer Research* 27 (2): 209–20.
McDonald, Daniel, Embriette Hyde, Justine W. Debelius, James T. Morton, Antonio Gonzalez, Gail Ackermann, Alexander A. Aksenov, et al. 2018. “American Gut: An Open Platform for Citizen Science Microbiome Research.” *mSystems* 3 (3). https://doi.org/10.1128/mSystems.00031-18.
McDonald, Daniel, Benjamin Kaehler, Antonio Gonzalez, Jeff DeReus, Gail Ackermann, Clarisse Marotz, Gavin Huttley, and Rob Knight. 2019. “Redbiom: A Rapid Sample Discovery and Feature Characterization System.” *mSystems* 4 (4). https://doi.org/10.1128/mSystems.00215-19.
Miller, J. A., K. Gandhi, and A. Gander. n.d. “A Survey of Citizen Science Gaming Experiences.” *Citizen*. https://doi.org/10.5334/cstp.500/print.
Mirarab, Siavash, Nam Nguyen, Sheng Guo, Li-San Wang, Junhyong Kim, and Tandy Warnow. 2015. “PASTA: Ultra-Large Multiple Sequence Alignment for Nucleotide and Amino-Acid Sequences.” *Journal of Computational Biology: A Journal of Computational Molecular Cell Biology* 22 (5): 377–86.
Mirarab, S., N. Nguyen, and T. Warnow. 2012. “SEPP: SATé-Enabled Phylogenetic Placement.” *Pacific Symposium on Biocomputing. Pacific Symposium on Biocomputing*, 247–58.
Mutalova, Renata, Roman Sarrazin-Gendron, Eddie Cai, Gabriel Richard, Parham Ghasemloo Gheidari, Sébastien Caisse, Rob Knight, Mathieu Blanchette, Attila Szantner, and Jérôme Waldispühl. 2023. “Playing the System: Can Puzzle Players Teach Us How to Solve Hard Problems?” In *Proceedings of the 2023 CHI Conference on Human Factors in Computing Systems*, 1–15. CHI ’23 679. New York, NY, USA: Association for Computing Machinery.
Nadkarni, Nalini M., and Jeremy S. Morris. 2018. “Baseline Attitudes and Impacts of Informal Science Education Lectures on Content Knowledge and Value of Science Among Incarcerated Populations.” *Science Communication* 40 (6): 718–48.
Nawrocki, Eric Paul. 2009. “Structural RNA Homology Search and Alignment Using Covariance Models.” Ann Arbor, United States: search.proquest.com. https://search.proquest.com/openview/5227539f9b37c2509352f9c4fe44e034/1?pq-origsite=gscholar&cbl=18750.
Nawrocki, Eric P., and Sean R. Eddy. 2013. “Infernal 1.1: 100-Fold Faster RNA Homology Searches.” *Bioinformatics* 29 (22): 2933–35.
Needleman, Saul B., and Christian D. Wunsch. 1989. “A General Method Applicable to the Search for Similarities in the Amino Acid Sequence of Two Proteins.” *Molecular Biology*. https://doi.org/10.1016/b978-0-12-131200-8.50031-9.

Nikulin, A., A. Serganov, E. Ennifar, S. Tishchenko, N. Nevskaya, W. Shepard, C. Portier, et al. 2000. “Crystal Structure of the S15-rRNA Complex.” *Nature Structural Biology* 7 (4): 273–77.
Nishikawa, Katsuo A., and Joseph Jaeger. 2011. “A Computer Simulation Comparing the Incentive Structures of Dictatorships and Democracies.” *Journal of Political Science Education* 7 (2): 135–42.
Nuyens, Filip M., Daria J. Kuss, Olatz Lopez-Fernandez, and Mark D. Griffiths. 2019. “The Empirical Analysis of Non-Problematic Video Gaming and Cognitive Skills: A Systematic Review.” *International Journal of Mental Health and Addiction* 17 (2): 389–414.
Peng, Wei. 2009. “Design and Evaluation of a Computer Game to Promote a Healthy Diet for Young Adults.” *Health Communication* 24 (2): 115–27.
Perelló, Josep, Núria Ferran-Ferrer, Salvador Ferré, Toni Pou, and Isabelle Bonhoure. 2017. “High Motivation and Relevant Scientific Competencies through the Introduction of Citizen Science at Secondary Schools: An Assessment Using a Rubric Model.” *Citizen Inquiry*, 150–75.
Pilles, Kiona Natasha. 2018. *Unweighted Unifrac Is a Theoretically Better Measure for Dietary and Cardiometabolic Data*.
Price, C. Aaron, and Hee-Sun Lee. 2013. “Changes in Participants’ Scientific Attitudes and Epistemological Beliefs during an Astronomical Citizen Science Project.” *Journal of Research in Science Teaching* 50 (7): 773–801.
Price, Morgan N., Paramvir S. Dehal, and Adam P. Arkin. 2010. “FastTree 2--Approximately Maximum-Likelihood Trees for Large Alignments.” *PloS One* 5 (3): e9490.
Rahman, Gibraan, Daniel McDonald, Antonio Gonzalez, Yoshiki Vázquez-Baeza, Lingjing Jiang, Climent Casals-Pascual, Shyamal Peddada, et al. n.d. “Scalable Power Analysis and Effect Size Exploration of Microbiome Community Differences with Evident.” https://doi.org/10.1101/2022.05.19.492684.
Ranwez, Vincent, and Olivier Gascuel. 2002. “Improvement of Distance-Based Phylogenetic Methods by a Local Maximum Likelihood Approach Using Triplets.” *Molecular Biology and Evolution* 19 (11): 1952–63.
Raposo, Kevin. 2015. “EyeWire Is Making Neuroscience Research Cool Again.” *TechCrunch*, May 1, 2015. https://techcrunch.com/2015/05/01/eyewire-is-making-neuroscience-research-cool-again/.
Roberts, Mary Roduta, Grace Reid, Meadow Schroeder, and Stephen P. Norris. 2013. “Causal or Spurious? The Relationship of Knowledge and Attitudes to Trust in Science and Technology.” *Public Understanding of Science* 22 (5): 624–41.
Robinson, D. F., and L. R. Foulds. 1981. “Comparison of Phylogenetic Trees.” *Mathematical Biosciences* 53 (1-2): 131–47.
Rofieq, A., and A. Fauzi. 2022. “Students’ Knowledge and Attitudes toward Science: Its Correlation on Students’ Disbelief in Non-Scientific Misinformation.” *Jurnal Pendidikan IPA Indonesia* 11 (2): 195–207.
Santori, Claudia, Ryan J. Keith, Camilla M. Whittington, Michael B. Thompson, James U. Van Dyke, and Ricky-john Spencer. 2021. “Changes in Participant Behaviour and Attitudes Are Associated with Knowledge and Skills Gained by Using a Turtle Conservation Citizen Science App.” *People and Nature (Hoboken, N.J.)* 3 (1): 66–

76.
Sasson, Irit. 2014. “The Role of Informal Science Centers in Science Education: Attitudes, Skills, and Self-Efficacy.” *JOTSE: Journal of Technology and Science Education* 4 (3): 167–79.
Serganov, A. A., B. Masquida, E. Westhof, C. Cachia, C. Portier, M. Garber, B. Ehresmann, and C. Ehresmann. 1996. “The 16S rRNA Binding Site of Thermus Thermophilus Ribosomal Protein S15: Comparison with Escherichia Coli S15, Minimum Site and Structure.” *RNA* 2 (11): 1124–38.
Suh, S., S. W. Kim, and N. J. Kim. 2010. “Effectiveness of MMORPG-Based Instruction in Elementary English Education in Korea.” *Journal of Computer Assisted Learning* 26 (5): 370–78.
Tang, Jian, and Nathan R. Prestopnik. 2023. “Science Gamers, Citizen Scientists, and Dabblers: Characterizing Player Engagement in Two Citizen Science Games.” *International Journal of Human–Computer Interaction* 39 (8): 1690–1711.
Toomey, Anne H., and Margret C. Domroese. 2013. “Can Citizen Science Lead to Positive Conservation Attitudes and Behaviors?” *Human Ecology Review* 20 (1): 50–62.
Trumbull, Deborah J., Rick Bonney, Derek Bascom, and Anna Cabral. 2000. “Thinking Scientifically during Participation in a Citizen-Science Project.” *Science Education* 84 (2): 265–75.
Van Der Pol, William J., Ranjit Kumar, Casey D. Morrow, Eugene E. Blanchard, Christopher M. Taylor, David H. Martin, Elliot J. Lefkowitz, and Christina A. Muzny. 2019. “In Silico and Experimental Evaluation of Primer Sets for Species-Level Resolution of the Vaginal Microbiota Using 16S Ribosomal RNA Gene Sequencing.” *The Journal of Infectious Diseases* 219 (2): 305–14.
Vohland, Katrin, Anne Land-Zandstra, Luigi Ceccaroni, Rob Lemmens, Josep Perelló, Marisa Ponti, Roeland Samson, and Katherin Wagenknecht. 2021. *The Science of Citizen Science*. Springer Nature.
Waldispuhl, Jerome, and Mathieu Blanchette. 2014. “Phylo and Open-Phylo: A Human-Computing Platform for Comparative Genomics.” *Proceedings of the AAAI Conference on Human Computation and Crowdsourcing* 2 (September): 73–74.
Waldispühl, Jérôme, Attila Szantner, Rob Knight, Sébastien Caisse, and Randy Pitchford. 2020. “Leveling up Citizen Science.” *Nature Biotechnology* 38 (10): 1124–26.
Walker, David W., Magdalena Smigaj, and Masakazu Tani. 2021. “The Benefits and Negative Impacts of Citizen Science Applications to Water as Experienced by Participants and Communities.” *WIREs. Water* 8 (1). https://doi.org/10.1002/wat2.1488.
Washburne, Alex D., Justin D. Silverman, James T. Morton, Daniel J. Becker, Daniel Crowley, Sayan Mukherjee, Lawrence A. David, and Raina K. Plowright. 2019. “Phylofactorization: A Graph Partitioning Algorithm to Identify Phylogenetic Scales of Ecological Data.” *Ecological Monographs* 89 (2): e01353.
Westcott, Kevin, and Jana Arbanas. 2021. “Gen Z Gamers Are Poised to Shake up the Media and Entertainment Industries for Good.” Fortune. April 19, 2021. https://fortune.com/2021/04/19/gen-z-gamers-video-games-media-entertainment-outlook-changes-future/.

Wiggins, Andrea, and Kevin Crowston. 2011. “From Conservation to Crowdsourcing: A Typology of Citizen Science.” In *2011 44th Hawaii International Conference on System Sciences*, 1–10.

Wintterlin, Florian, Friederike Hendriks, Niels G. Mede, Rainer Bromme, Julia Metag, and Mike S. Schäfer. 2022. “Predicting Public Trust in Science: The Role of Basic Orientations toward Science, Perceived Trustworthiness of Scientists, and Experiences with Science.” *Frontiers in Communication* 6 (January). https://doi.org/10.3389/fcomm.2021.822757.

Woese, C. R., L. J. Magrum, R. Gupta, R. B. Siegel, D. A. Stahl, J. Kop, N. Crawford, et al. 1980. “Secondary Structure Model for Bacterial 16S Ribosomal RNA: Phylogenetic, Enzymatic and Chemical Evidence.” *Nucleic Acids Research* 8 (10): 2275–93.

Wulf, Rosemary, Laurel M. Mayhew, Noah D. Finkelstein, Chandralekha Singh, Mel Sabella, and Sanjay Rebello. 2010. “Impact of Informal Science Education on Children’s Attitudes about Science.” In *AIP Conference Proceedings*. AIP. https://doi.org/10.1063/1.3515238.

Yang, Bo, Yong Wang, and Pei-Yuan Qian. 2016. “Sensitivity and Correlation of Hypervariable Regions in 16S rRNA Genes in Phylogenetic Analysis.” *BMC Bioinformatics* 17 (March): 135.

Yee, Nick. 2006. “Motivations for Play in Online Games.” *Cyberpsychology & Behavior: The Impact of the Internet, Multimedia and Virtual Reality on Behavior and Society* 9 (6): 772–75.

Young, Brian, Tom Faris, and Luigi Armogida. 2021. “Levenshtein Distance as a Measure of Accuracy and Precision in Forensic PCR-MPS Methods.” *Forensic Science International. Genetics* 55 (November): 102594.
