# Supplementary material for: Improving microbial phylogeny with citizen science within a mass-market video game
Source: Nat Biotechnol. 2024 Apr 15;43(1):76–84. doi: 10.1038/s41587-024-02175-6 (PMC11738981; doi:10.1038/s41587-024-02175-6)
Supplement: Supplementary file 2 — Reporting Summary [file 41587_2024_2175_MOESM2_ESM.pdf]

nature portfolio

Corresponding author(s): Jerome Waldispuhl

Last updated by author(s): Jan 23, 2024

Reporting Summary

Nature Portfolio wishes to improve the reproducibility of the work that we publish. This form provides structure for consistency and transparency in reporting. For further information on Nature Portfolio policies, see our Editorial Policies and the Editorial Policy Checklist.

Statistics

For all statistical analyses, confirm that the following items are present in the figure legend, table legend, main text, or Methods section.

n/a	Confirmed	
☐	☒	The exact sample size (*n*) for each experimental group/condition, given as a discrete number and unit of measurement
☒	☐	A statement on whether measurements were taken from distinct samples or whether the same sample was measured repeatedly
☐	☒	The statistical test(s) used AND whether they are one- or two-sided<br>*Only common tests should be described solely by name; describe more complex techniques in the Methods section.*
☒	☐	A description of all covariates tested
☐	☒	A description of any assumptions or corrections, such as tests of normality and adjustment for multiple comparisons
☐	☒	A full description of the statistical parameters including central tendency (e.g. means) or other basic estimates (e.g. regression coefficient) AND variation (e.g. standard deviation) or associated estimates of uncertainty (e.g. confidence intervals)
☐	☒	For null hypothesis testing, the test statistic (e.g. *F*, *t*, *r*) with confidence intervals, effect sizes, degrees of freedom and *P* value noted<br>*Give P values as exact values whenever suitable.*
☒	☐	For Bayesian analysis, information on the choice of priors and Markov chain Monte Carlo settings
☒	☐	For hierarchical and complex designs, identification of the appropriate level for tests and full reporting of outcomes
☐	☒	Estimates of effect sizes (e.g. Cohen's *d*, Pearson's *r*), indicating how they were calculated

Our web collection on statistics for biologists contains articles on many of the points above.

Software and code

Policy information about availability of computer code

Data collection	The data was collected within the commercial video game Borderlands 3, via exchanges with the Massively Multiplayer Online Science (MMOS) server. The exchange with servers and construction of local databases are performed with custom code in python. As this is a citizen science game project, the data collection aspect is not directly reproducible without the game, so we made the data and processing scripts fully open source. The data organization code is available at https://games.cs.mcgill.ca/bls/<br>The Greengenes data featured in our analysis comes from the Greengenes 13.5 FTP repository: https://greengenes.secondgenome.com/?prefix=downloads/greengenes_database/gg_13_5/ . The pyNAST and ssu-align alignments mentioned in the paper come from this repository.<br>Rfam data comes from the bacterial small ribosomal subunit family on Rfam 14: https://rfam.org/family/SSU_rRNA_bacteria<br>We also feature data from the Comparative RNA website: https://crw-site.chemistry.gatech.edu/CAR/1A/#rrna
Data analysis	Every single line of analysis code is open source and already available. It is coded in Python 3.8 and Bash, and makes use of many previously published python libraries, most notably QIIME version 2, FastTree version 2.1, scikit-bio version 0.5, evident version 0.4. A full list of the libraries used is available on FigShare: https://www.doi.org/10.6084/m9.figshare.24962349. All data and code is also available at https://games.cs.mcgill.ca/bls/<br>Benchmarks were computed with MAFFT v7.490, MUSCLE v5.1.0, PASTA 1.9.0, FastTree 2.1.10

For manuscripts utilizing custom algorithms or software that are central to the research but not yet described in published literature, software must be made available to editors and reviewers. We strongly encourage code deposition in a community repository (e.g. GitHub). See the Nature Portfolio guidelines for submitting code & software for further information.

Data

Policy information about availability of data

All manuscripts must include a data availability statement. This statement should provide the following information, where applicable:

- Accession codes, unique identifiers, or web links for publicly available datasets
- A description of any restrictions on data availability
- For clinical datasets or third party data, please ensure that the statement adheres to our policy

All the data used in this manuscript can be publicly accessed at https://games.cs.mcgill.ca/bls/ and on FigShare: https://www.doi.org/10.6084/m9.figshare.24962349, as well as at https://gitlab.com/borderlands-science/BLS1. This includes all the puzzles players solved, all the solutions submitted by the players, and related data such as the order in which they made their moves to solve the puzzle. The players are identified by unique alphanumeric strings as it was not possible to obtain their informed consent to share their personal information as this data was collected through a videogame.

Human research participants

Policy information about studies involving human research participants and Sex and Gender in Research.

Reporting on sex and gender	We do not know the sex or gender of the participants, thus there is no reporting on that.
Population characteristics	We do not have any personal information about the participants and are thus not reporting characteristics.
Recruitment	Participants were not recruited; the data was collected from a mini-game within a commercial videogame. The mini-game was represented as a virtual arcade machine within the game and. Upon approaching the machine, the player was presented with a video explaining the project and that the solutions to the mini-game would be used for a scientific project on the human gut microbiome. The player could then choose to solve tasks in the scientific game, or just continue with regular gameplay in the commercial game
Ethics oversight	As no one was recruited for this project and data collection was entirely opt-in with no personal information connected, the study was not considered to require ethics oversight at McGill University.

Note that full information on the approval of the study protocol must also be provided in the manuscript.

Field-specific reporting

Please select the one below that is the best fit for your research. If you are not sure, read the appropriate sections before making your selection.

☒ Life sciences ☐ Behavioural & social sciences ☐ Ecological, evolutionary & environmental sciences

For a reference copy of the document with all sections, see nature.com/documents/nr-reporting-summary-flat.pdf

Life sciences study design

All studies must disclose on these points even when the disclosure is negative.

Sample size	There were no sample size calculations as every point of data at the time of starting analysis was used. Indeed, we started performing this analysis in July 2021, and used every single datapoint collected from the release of the game on April 7, 2020 to early July 2021. This represents the majority of the total data collected over the lifetime of the game, and later tests were performed with different methodologies that are not involved in the paper. In other words, this paper uses 100% of the data that matches the described methodology.
Data exclusions	Each puzzle was solved an average of 43 times. Filtering was applied to identify the highest quality solutions to each puzzle based on Pareto-optimality and proximity to the player consensus. The parameters for this exclusion were determined experimentally, but with the same parameters for every single element of a very heterogeneous dataset of over 70 million puzzle solutions.
Replication	The ensure the reproducibility of this study, we took measures to prevent overfitting to subsets of our data by optimizing our pipeline against phylogeny rather than phenotype, relying on prior research showing a correlation between the two. This gives us higher confidence that this study's results are reproducible on other datasets/samples of microbiome data.
Randomization	The allocation of participants/players to individual puzzles was done entirely randomly. Outside of that, there was no grouping done for the study.
Blinding	The investigators were de facto blinded due to the fact they had no information on the participants. Indeed, the research team only had unique alphanumeric strings to identify participants since they could not consent to their information being shared by Gearbox Entertainment, the editor of the game.

Reporting for specific materials, systems and methods

We require information from authors about some types of materials, experimental systems and methods used in many studies. Here, indicate whether each material, system or method listed is relevant to your study. If you are not sure if a list item applies to your research, read the appropriate section before selecting a response.

Materials & experimental systems

n/a	Involved in the study
☒	☐ Antibodies
☒	☐ Eukaryotic cell lines
☒	☐ Palaeontology and archaeology
☒	☐ Animals and other organisms
☒	☐ Clinical data
☒	☐ Dual use research of concern

Methods

n/a	Involved in the study
☒	☐ ChIP-seq
☒	☐ Flow cytometry
☒	☐ MRI-based neuroimaging
